# CD9^+^ B Cells Induce T Follicular Helper Cell Apoptosis to Regulate Germinal Center Regression

**DOI:** 10.1002/advs.202502144

**Published:** 2026-08-30

**Authors:** Wenjing Liao, Lijuan Song, Meiqian Xu, Tianhao Liang, Yixuan Chen, Gui Chen, Zaihuan Lin, Huakang Du, Guofei Feng, Zhiming Feng, Qi Zhang, Fan Pan, Saixuan Yang, Xinyin Zhang, Wenxu Peng, Tianlong Li, Zhuoshen Lin, Yabang Chen, Jiayi Liu, Baoxin Peng, Yan Liu, Xing Niu, Ting Li, Chengyu Huang, Ke Mo, Jianlei Xie, Yu Zou, Xiaowen Zhang

**Affiliations:** ^1^ State Key Laboratory of Respiratory Disease Department of Otolaryngology‐Head and Neck Surgery Loudi Central Hospital The First Affiliated Hospital of Guangzhou Medical University Loudi Hunan China; ^2^ State Key Laboratory of Respiratory Disease Department of Otolaryngology‐Head and Neck Surgery The First Affiliated Hospital of Guangzhou Medical University Guangzhou Guangdong China; ^3^ Department of Otolaryngology‐Head and Neck Surgery Guangdong Women and Children Hospital Guangzhou Guangdong China; ^4^ Department of Basic Science YuanDong International Academy of Life Sciences Hong Kong China; ^5^ Department of Otolaryngology‐Head and Neck Surgery The People's Hospital of Kaizhou District Chongqing China

**Keywords:** adenoid hypertrophy, CD9, FOXP1, germinal center B cell, Tfh cells

## Abstract

Adenoid hypertrophy (AH) is associated with excessive proliferation of germinal center B (GC‐B) cells, yet the mechanisms underlying GC regression and termination remain poorly understood. This study used single‐cell RNA sequencing (scRNA‐seq) to profile the cellular composition of GC‐B cells in AH. A unique subset of GC‐B cells expressing CD9 was identified through comprehensive scRNA‐seq, flow cytometry, and Mass cytometry (CyToF) analyses, with subsequent studies assessing the roles of CD9 and FOXP1 in GC‐B cell differentiation and apoptosis pathways. CD9^+^ B cells were detected throughout GC‐B cell development and were regulated by FOXP1. These cells also exhibited activation of cell death‐related pathways, particularly during adenoid development. AH samples showed an increase in T follicular helper (Tfh) cells, accompanied by a reduction in CD9^+^ B cells. In Cd9 knockout mice, CD9 deficiency led to a significant decrease in serum IgG levels. Further analysis revealed that CD9^+^ B cells promoted Tfh cell apoptosis via pathways such as ALCAM‐CD6. CD9^+^ B cells may regulate GC‐B cell regression by inducing Tfh cell apoptosis, and FOXP1 may play a role in their differentiation. These findings clarify mechanisms of GC degeneration and termination, offering potential targets for AH treatment.

## Graphical abstract

A unique subset of CD9+ B cells regulates germinal center B (GC‐B) cell regression by inducing T follicular helper (Tfh) cell apoptosis through multiple signaling pathways, such as ALCAM‐CD6. FOXP1 plays a key role in GC‐B cell differentiation. These findings clarify mechanisms of GC degeneration and termination and offer potential targets for adenoid hypertrophy treatment.

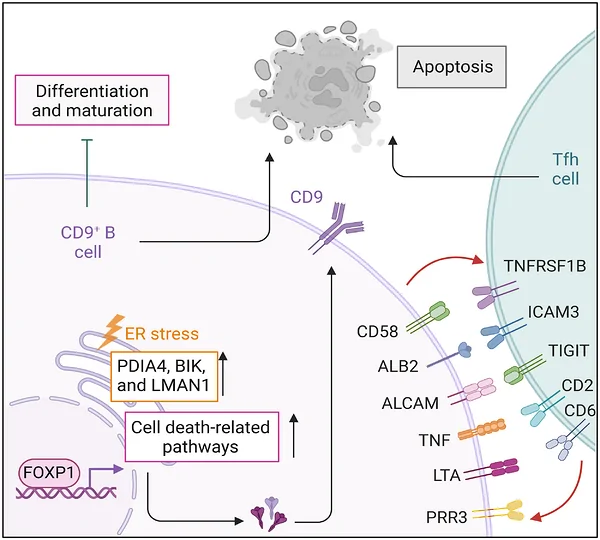

## 1 Introduction

Adenoids, or pharyngeal tonsils, are part of Waldeyer's ring and play a crucial role in nasopharyngeal immunity, particularly in children [1]. Adenoid hypertrophy (AH) is a common pediatric respiratory condition, with a prevalence ranging from 9.9% to 29.9% and an increasing incidence annually [2]. Clinical symptoms, such as nasal congestion, coughing, snoring, and mouth breathing, are frequently associated with AH and can lead to complications like obstructive sleep apnea and craniofacial abnormalities. While adenoidectomy is an effective treatment for AH, it carries risks including anesthesia‐related complications, postoperative bleeding, and potential asphyxiation [3, 4, 5]. Pharmacological options, such as nasal glucocorticoids and leukotriene receptor antagonists, are also used to manage AH, though their effectiveness is often limited [6, 7, 8].

In adenoid tissue, lymphocytes are the predominant cell type, with B cells constituting 60–70% of the total cell population [9]. When activated by specific antigens and supported by T cells, these B lymphocytes form germinal centers (GCs), where they differentiate into plasma cells and memory B cells, contributing to immune regulation through antibody and cytokine production [10]. Although much is known about the formation of GCs, the mechanisms driving their regression and eventual termination remain poorly understood. Interactions between T follicular helper (Tfh) cells and B cells are thought to be pivotal in these processes [11, 12], as Tfh‐B cell interactions are crucial for GC formation and maintenance. Therefore, understanding the interactions between these cells is crucial for uncovering the mechanisms underlying GC degradation.

Human adenoid development and atrophy are age‐dependent, with complete atrophy generally occurring in adulthood [13, 14]. Similar to the thymus, an age‐related lymphoid organ, factors influencing adenoid growth and atrophy are being explored [15]. However, rodent models commonly used in immunology research lack adenoids, and there are significant differences between the mouse and human immune systems, particularly in B‐cell biology [16], which makes adenoid research difficult using mouse models. While adenoid tissue can be obtained from routine surgeries and contains a large proportion of GC B cells [1, 17], explant cultures typically maintain cell composition for only 3–4 days and fail to capture all aspects of the adaptive immune response.

Advances in single‐cell RNA sequencing (scRNA‐seq) offer a high‐resolution approach to studying gene expression in complex cellular systems [18, 19, 20]. Although a previous study has generated a single‐cell atlas of the human tonsil and provided an important reference for characterizing the cellular complexity of the organ [21], there are significant differences in histological structure, immune defense mechanisms, and clinical biology between the tonsil and adenoid tissues. In particular, the adenoids undergo a typical natural atrophy process from childhood to adolescence, whereas the tonsils remain intact after puberty. Furthermore, the immune defense capability of the tonsils gradually declines until they are lost [22, 23]. The different developmental trajectories of the adenoids and tonsils suggest that they may exhibit different immune responses when facing the same pathogen. Therefore, the tonsil data cannot be extrapolated to pediatric adenoid pathological research. To better understand the regulatory mechanisms involved in GC formation and regression, we performed scRNA‐seq on clinical adenoid samples and generated a single‐cell atlas of GC‐B cells in adenoid tissue. Our study identified key cell subpopulations and molecular regulators involved in adenoid development and atrophy. Notably, we discovered a distinct subset of CD9^+^ B cells that plays a critical role in GC‐B cell differentiation. We also uncovered the regulatory role of FOXP1 in the differentiation of CD9^+^ B cells. Our findings suggest that CD9^+^ B cells promote Tfh cell apoptosis via multiple signaling pathways, including ALCAM‐CD6, contributing to adenoid atrophy. These insights further elucidate the molecular interactions between Tfh and B cells in regulating GC regression and termination.

## 2 Results

### 2.1 Single‐Cell Landscape of Adenoid and Tonsil Tissues

**FIGURE 1:**
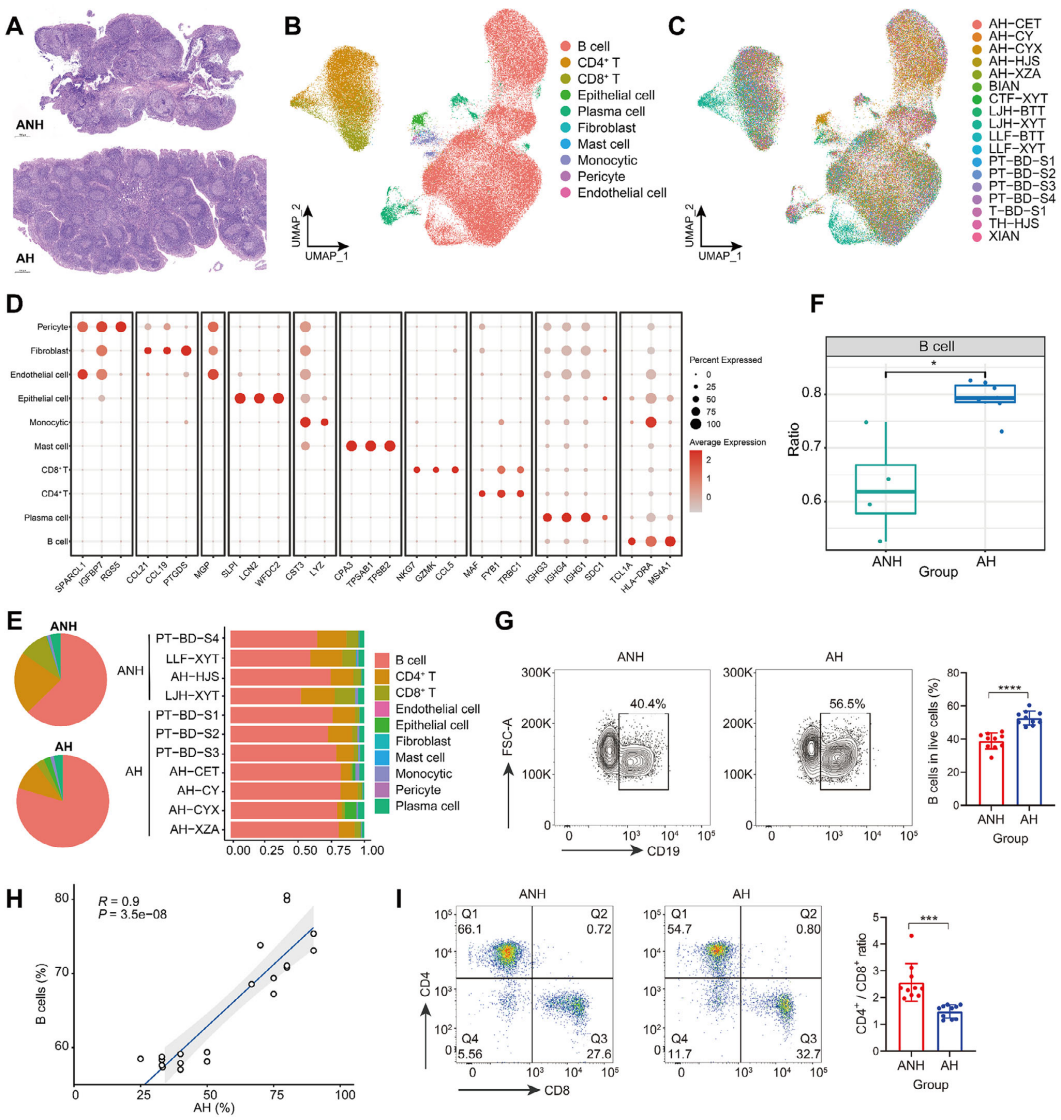
Single‐cell landscapes of adenoidal tissue during adenoid development and atrophy. (A) H&E staining of adenoidal non‐hypertrophy (ANH) and adenoidal hypertrophy (AH) tissues (scale bar, 500 µm). (B) Single‐cell atlas mapping the distribution of different cell types in tonsil and adenoid tissues. (C) Single‐cell atlas mapping the distribution of different cell types in samples. (D) Specific expression of cell type marker genes in each cell type. (E) Abundance ratios of each cell type in ANH and AH groups and samples. (F) The abundance ratio of B cells in ANH and AH groups (*p < 0.05). (G) Flow cytometry showing that the abundance of B cells in the AH group was increased compared with that in the ANH group (****p < 0.0001). (H) Correlation plot showing that the abundance of B cells was significantly positively correlated with adenoidal hypertrophy (AH) (%). AH (%) = adenoid obstruction area / nasopharyngeal airway area × 100%. (I) Flow cytometry showing that the ratio of CD4^+^ / CD8^+^ T cells in the AH group was reduced compared with that in the ANH group (***p < 0.001).

To characterize the molecular features of each cell type in Waldeyer's ring lymphoid tissues, we analyzed the transcriptomes of each cell population using scRNA‐seq and explored the potential role of GC‐B cells in adenoid development (Figure S1). This study utilized scRNA‐seq to analyze 13 adenoid and 5 tonsil samples. Compared with ANH, AH tissues exhibited significant pathological differences (Figure 1A). The scRNA‐seq data were generated using 10x Genomics and BD Rhapsody platforms and were integrated using the Harmony algorithm. After quality control (QC) and integration, a total of 91,741 cells from 18 samples were visualized via UMAP (19,094 from BD Rhapsody and 72,647 from 10x Genomics) (Figure 1B,C). Cell clusters were identified based on specific marker genes: B cells (TCL1A, MS4A1), plasma cells (IGHG3, IGHG4, IGHG1, SDC1), CD4^+^ T cells (MAF, TRBC1), CD8^+^ T cells (NKG7, GZMK), mast cells (CPA3, TPSAB1), monocytes (HLA‐DRA, CST3), epithelial cells (SLPI, LCN2), endothelial cells (MGP, SPARCL1), fibroblasts (CCL21, CCL19), and pericytes (IGFBP7, RGS5) (Figure 1D).

Among the 13 adenoid samples, 11 samples were clearly labeled as either adenoid hypertrophy (AH) or adenoid non‐hypertrophy (ANH) (Table S1). Two samples, aged 31 and 36 years old, were from “adult adenoid residual tissues” and did not meet the study requirements for consistency and biological background. Therefore, we excluded them before subsequent analysis. Compared with ANH, AH tissues showed a significant increase in B cell abundance and a marked decrease in CD4^+^ and CD8^+^ T cells (Figure 1E,F). To further validate this finding, we collected adenoid tissues from 10 ANH patients and 10 AH patients and used flow cytometry to assess the proportion of B cells in these tissues. The results showed that B cell proportion in AH samples was significantly higher than that in ANH samples (Figure 1G, Figure S3A). Additionally, there was a positive correlation between B cell proportion and adenoid size (Figure 1H; R = 0.9 and p = 3.5e‐08). Flow cytometry also confirmed a significant reduction in CD4^+^ / CD8^+^ T cell ratio in AH compared with ANH (Figure 1I, Figure S3B).

### 2.2 GC‐B Cell Ecology of Adenoid Tissue During Adenoid Development

**FIGURE 2:**
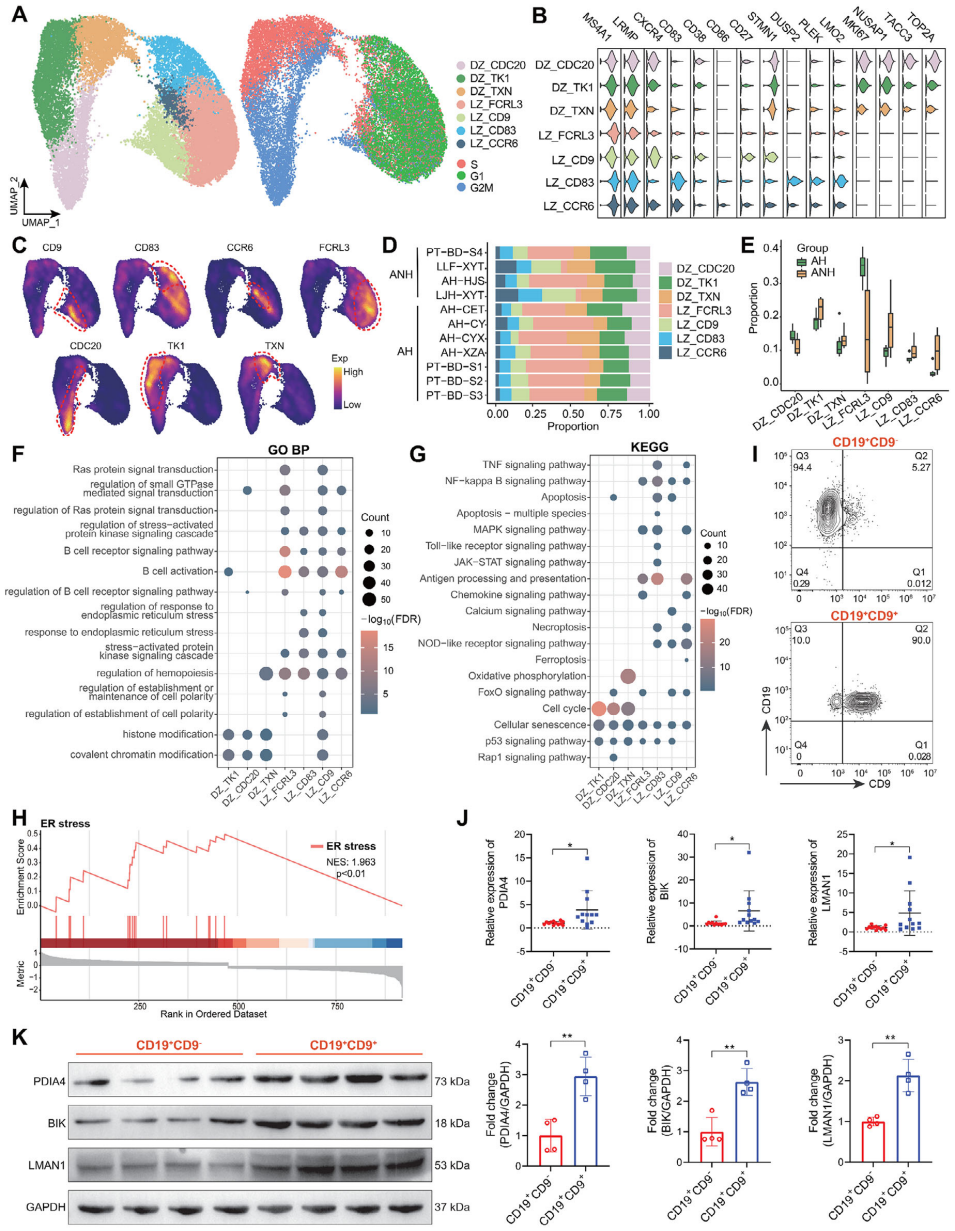
Germinal center B (GC‐B) cell ecology of adenoid tissue during adenoid development and atrophy. (A) Single‐cell atlas mapping GC‐B cell subsets in adenoidal tissues. (B) Violin plots showing the expression of light zone (LZ) and dark zone (DZ) markers in GC‐B cells. (C) Density plots showing the expression of specific markers for GC‐B cell subsets. (D) Proportions of GC‐B cell subsets in different samples. (E) Proportions of GC‐B cell subsets in AH and ANH groups. (F) Gene Ontology (GO) biological processes (BPs) that were significantly enriched in the GC‐B subset. (G) Kyoto Encyclopedia of Genes and Genomes (KEGG) pathways that were significantly enriched in GC‐B cell subsets. (H) Gene set enrichment analysis (GSEA) showing that the endoplasmic reticulum (ER) stress gene set was significantly activated in the CD9^+^ GC‐B subset. (I) Flow cytometry for validating the sorted CD19^+^CD9^−^ B cells and CD19^+^CD9^+^ B cells. (J) RT‐qPCR experiments showing that ER stress‐related genes were upregulated in CD9^+^ B cells compared with CD9^−^ B cells (*p < 0.05). (K) Western blot experiments showing that ER stress‐related genes were upregulated in CD9^+^ B cells compared with CD9^−^ B cells (**p < 0.01).

To investigate the ecological role of GC‐B cells during adenoid development, we performed clustering analysis on GC‐B cells. GC cells can be divided into two regions: the dark zone (DZ) and the light zone (LZ). DZ cells are characterized by high expression of MKI67 and STMN1, while LZ cells show elevated expression of CD83 [24]. Based on the markers and histological characteristics of GC‐B cells, we classified them into DZ and LZ categories. A total of seven GC‐B cell subpopulations were identified in this study (Figure 2A,B), each expressing distinct specific markers (Figure 2C). Three DZ‐related clusters (DZ_CDC20, DZ_TK1, and DZ_TXN) were significantly enriched in the S/G2M phase and exhibited high expression of classic proliferation‐ or spindle assembly‐related genes such as MKI67, NUSAP1, TACC3, and TOP2A (Figure 2A,B). The CD9^+^ B cells were classified as LZ_CD9 because they were mainly enriched in the G1 phase and exhibited low expression of proliferation‐related genes and high expression of classic LZ marker genes, such as CD83, CD86, and CD27, despite expressing STMN1 and CXCR4. We also explored the differences in the abundance of GC‐B subpopulations across different samples and found that the ecological composition of GC‐B cells was consistent across samples (Figure 2D). However, compared with the ANH group, the abundance of CD9^+^ B cells was significantly reduced in the AH group (Figure 2E). CD9 is a tetraspanin protein involved in various biological processes such as cell motility, proliferation, differentiation, fusion, and adhesion [25]. CD9 has previously been identified as a marker of regulatory B (Breg) cells [26]. We observed that CD9^+^ B cells expressed several classic Breg markers, including CD24, CD38, and CD27 (Table S2), indicating that there may be a certain degree of overlap between CD9^+^ B cells and Breg cells. However, the role of CD9^+^ B cells in adenoid development remains unclear.

Enrichment analysis revealed significant differences in the biological processes (BPs) and pathways enriched in LZ‐ and DZ‐B cells (Figure 2F,G). Processes such as histone modification and covalent chromatin modification were significantly activated in DZ‐B cells, whereas B cell activation and B cell receptor signaling pathways were notably enriched in LZ‐B cells (p < 0.05). Among them, B cell activation and endoplasmic reticulum (ER) stress‐related BPs were significantly activated in CD9^+^ B cells (p < 0.05), which was further validated through gene set enrichment analysis (GSEA) (Figure 2H). We then sorted CD9^+^ B cells for further validation (Figure 2I) and found that, compared with CD9^−^ B cells, CD9^+^ B cells showed significant upregulation of ER stress‐related genes, including PDIA4, BIK, and LMAN1 (Figure 2J). Moreover, Western blot analysis confirmed that ER stress markers were more highly expressed in CD9^+^ B cells than in CD9^−^ B cells (Figure 2K, Figure S4A). In summary, this study preliminarily identified the major GC‐B cell subpopulations during adenoid development and found that the abundance of CD9^+^ B cells was significantly increased in the ANH group, suggesting their potential involvement in regulating adenoid atrophy.

### 2.3 FOXP1 May be a Potential Regulon of CD9+ B Cell Differentiation in Adenoid Tissue

**FIGURE 3:**
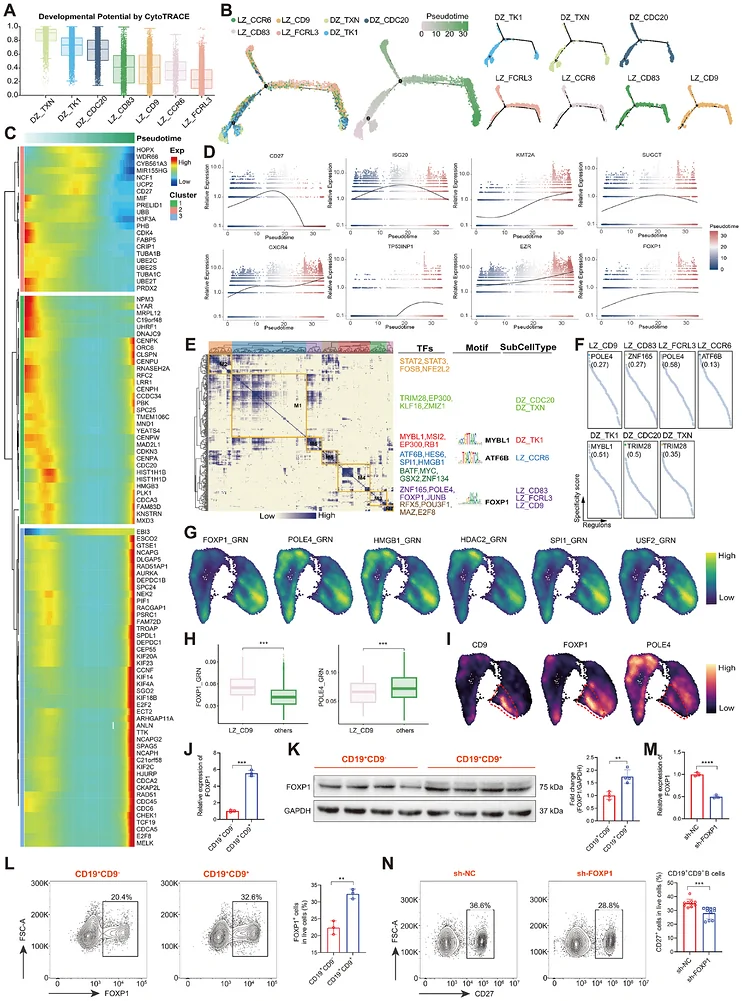
Pseudotime developmental trajectories and gene regulatory network analysis of GC‐B cell subsets. (A) Differentiation potential of GC‐B cell subsets assessed by cytoTRACE. (B) Differentiation trajectories of GC‐B cell subsets inferred by Monocle2. (C) Heatmap of gene expression during lineage development of GC‐B cell subsets. (D) Dynamic gene expression during lineage development of GC‐B cell subsets. (E) Gene regulatory network (GRN) of GC‐B cell subsets based on pySCENIC method. The heatmap of the regulon co‐expression module, the main transcription factors (TFs) of the regulatory module, the motifs of the specified TFs, and the regulated GC‐B cell subsets are shown, respectively. (F) The regulon with the highest regulon specificity score (RSS) in each GC‐B cell subset. (G) Density plots mapping the activity scores of TFs in GC‐B cell subsets. (H) Box plots showing the difference in the activity scores of FOXP1 and POLE4 between CD9^+^ B cells and other GC‐B cell subsets (***p < 0.001). (I) Density plots mapping the transcriptome expression of TFs. (J) RT‐qPCR experiments showing the expression of FOXP1 in CD19^+^CD9^−^ B cells and CD19^+^CD9^+^ B cells (***p < 0.001). (K) Western blot experiments showing FOXP1 expression in CD19^+^CD9^−^ B cells and CD19^+^CD9^+^ B cells (**p < 0.01). (L) Flow cytometry showing the intracellular expression of FOXP1 in CD19^+^CD9^−^ B cells and CD19^+^CD9^+^ B cells (**p < 0.01). (M) RT‐qPCR experiments showing the expression of FOXP1 after FOXP1 shRNA and sh‐NC transfection into CD19^+^CD9^+^ B cells (****p < 0.0001). (N) Flow cytometry showing the levels of CD27 in CD19^+^CD9^+^ B cells after FOXP1 shRNA and sh‐NC transfection (***p < 0.001).

To further investigate the role of CD9^+^ B cells in adenoids, we performed pseudotime analysis on GC‐B cell subpopulations. Based on CytoTRACE assessment of the differentiation potential of GC‐B cells, DZ_TXN was identified as the starting point of cell differentiation (Figure 3A). Throughout the differentiation trajectory of GC‐B cell subpopulations (Figure 3B), CD9^+^ B cells were consistently distributed, suggesting that they span the entire differentiation trajectory of GC‐B cell subpopulations and may be essential for regulating adenoid development and atrophy. As the GC‐B cell subpopulations differentiated, genes with similar expression patterns were clustered into three groups. Genes in the intermediate state (Cluster 2) were primarily enriched in biological processes (BPs) and other pathways related to protein ubiquitination and apoptosis (Figure 3C). Furthermore, the expression of genes such as KMTA2, CXCR4, EZR, and FOXP1 gradually increased along the differentiation trajectory, suggesting that these genes may regulate the differentiation of GC‐B cell subpopulations (Figure 3D).

We conducted gene regulatory network (GRN) analysis to further explore potential regulators controlling CD9^+^ B cell differentiation. Based on the connection specificity index (CSI), 287 regulators of GC‐B cell subpopulations were clustered into seven modules (Table S3, Figure 3E). The largest module, M1, containing 116 regulators, primarily regulated the DZ_CDC20 and DZ_TXN subpopulations. Meanwhile, the regulators of CD9^+^ B cells were mostly derived from the M3 module, which included 26 regulators, such as POLE4, HMGB1, and FOXP1. The regulator specificity score (RSS) ranking enabled us to identify the most specific regulators across different GC‐B cell subpopulations. In CD9^+^ B cells, POLE4 had a high RSS (Figure 3F), but FOXP1 showed a more specific activity distribution, with higher expression of FOXP1 and lower expression of POLE4 in CD9^+^ B cells compared with other GC‐B cell subpopulations (Figure 3G,H). Notably, the expression pattern of FOXP1 more closely resembled that of CD9 compared with POLE4 (Figure 3I). Further experiments revealed that FOXP1 RNA and protein expression were elevated in CD9^+^ B cells compared with CD9^−^ cells (Figure 3J,K, Figure S4B). Flow cytometry also confirmed the increased intracellular FOXP1 expression in CD9^+^ B cells compared with CD9^−^ cells (Figure 3L, Figure S3C). Moreover, the proportion of CD19^+^CD9^+^CD27^+^ B cells was significantly reduced in the FOXP1 knockdown group (sh‐FOXP1) compared with that in the control group (sh‐NC) (Figure 3M,N, Figure S3C), suggesting that FOXP1 inhibition impairs the differentiation of CD9^+^ B cells.

In conclusion, our findings suggest that CD9^+^ B cells may be essential regulators of adenoid development and atrophy, and FOXP1 could be a potential regulator of CD9^+^ B cell differentiation in adenoid tissues.

### 2.4 CD9^+^ B Cells Are a Critical Cell Subset Regulating Adenoid Development and Atrophy

**FIGURE 4:**
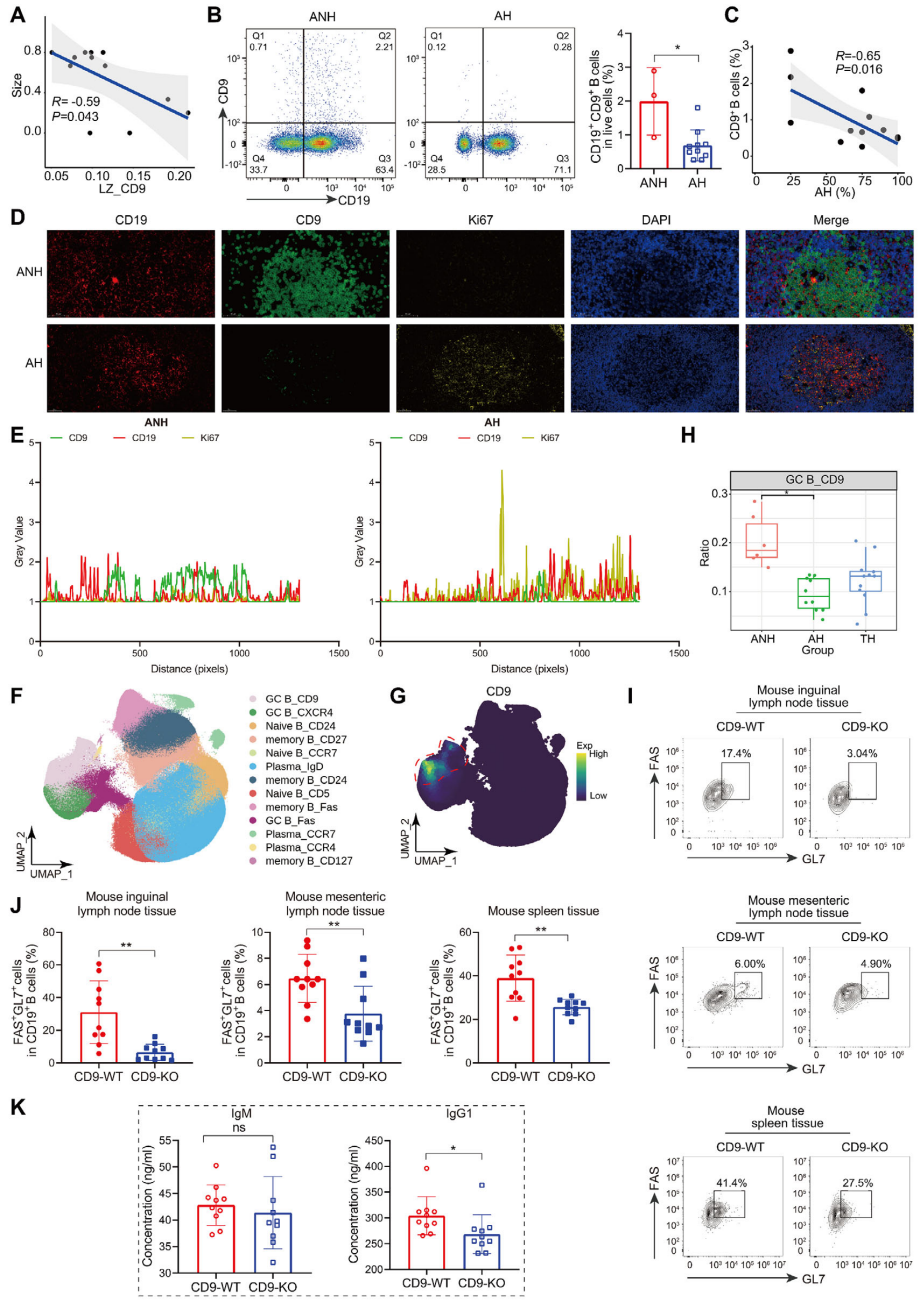
The relationship between CD9^+^ B cells abundance and adenoidal hypertrophy (AH). (A) Correlation plot showing that CD9^+^ B cell abundance ratio was significantly negatively correlated with adenoid size. (B) Flow cytometry showing that the abundance of CD9^+^ B cells was decreased in AH tissue (*p < 0.05). (C) Correlation diagram showing the negative correlation between the proportion of CD9^+^ B cells and AH (%) in flow cytometry experiments. (D, E) Multiple immunofluorescence showing the expression and distribution of CD9^+^ B cells in adenoid tissues of ANH and AH groups (scale bar, 50 µm). (F) Single‐cell atlas showing B cell subsets by CyToF mass spectrometry in AH, ANH, and tonsil (TH) tissues. (G) Density plot showing CD9 expression in B cell subsets. (H) Box plot showing the abundance ratio of CD9^+^ B cell subsets in ANH, AH, and TH tissues measured by CyToF mass spectrometry (*p < 0.05). (I, J) Flow cytometry showing the proportion of GC‐B cells in the inguinal lymph nodes, mesenteric lymph nodes, and spleen of Cd9 wild‐type (WT) and knockout (CD9‐KO) mice (**p < 0.01). (K) Detection of the concentrations of serum IgM and IgG1 in CD9‐WT and CD9‐KO mice using ELISA (ns: not significant, *p < 0.05).

The abundance of CD9^+^ B cells in adenoid tissues was significantly lower in the AH group, though their specific role in adenoids remains unclear. To explore this, we analyzed the relationship between CD9^+^ B cell abundance and adenoid size. We found a significant negative correlation between CD9^+^ B cell abundance and adenoid size (Figure 4A; R = ‐0.59 and p = 0.043). Flow cytometry further confirmed that CD9^+^ B cells were more abundant in the ANH group than in the AH group (Figure 4B, Figure S3A). There was also a notable negative correlation between the proportion of CD9^+^ B cells and the incidence of AH (Figure 4C; R = ‐0.65 and p = 0.016), with immunofluorescence corroborating the higher presence of CD9^+^ B cells in ANH tissues (Figure 4D, E). Mass cytometry (CyToF) analysis revealed that B cell subpopulations, including CD9^+^ B cells, were present in ANH, AN, and TH tissues (Figure 4F,G). Consistent with the single‐cell sequencing results, CD9^+^ B cells were significantly more abundant in the ANH group compared with the AH group, suggesting that these cells may be a key subpopulation regulating adenoid development and atrophy (Table S4, Figure 4H).

Next, we observed the functional mechanism of CD9 in GC‐B cells by comparing Cd9 knockout (CD9‐KO) mice with wild‐type (CD9‐WT) mice. Because mice lack adenoids, we observed the abundance of GC‐B cells in other peripheral lymphoid organs, including inguinal lymph nodes, mesenteric lymph nodes, and spleen. We found that the abundance of GC‐B cells in inguinal lymph nodes, mesenteric lymph nodes, and spleen of CD9‐KO mice was significantly reduced compared with that in CD9‐WT mice (Figure 4I,J, Figure S3D). Additionally, IgG1 expression was markedly lower in CD9‐KO mice (Figure 4K), indicating that CD9 expression may be closely linked to B cell immune function. Taken together, these findings suggest that CD9^+^ B cells may play a crucial role in regulating adenoid development.

### 2.5 CD9^+^ B Cells Significantly Activate Cell Death‐related Signaling During Adenoid Development Stages

**FIGURE 5:**
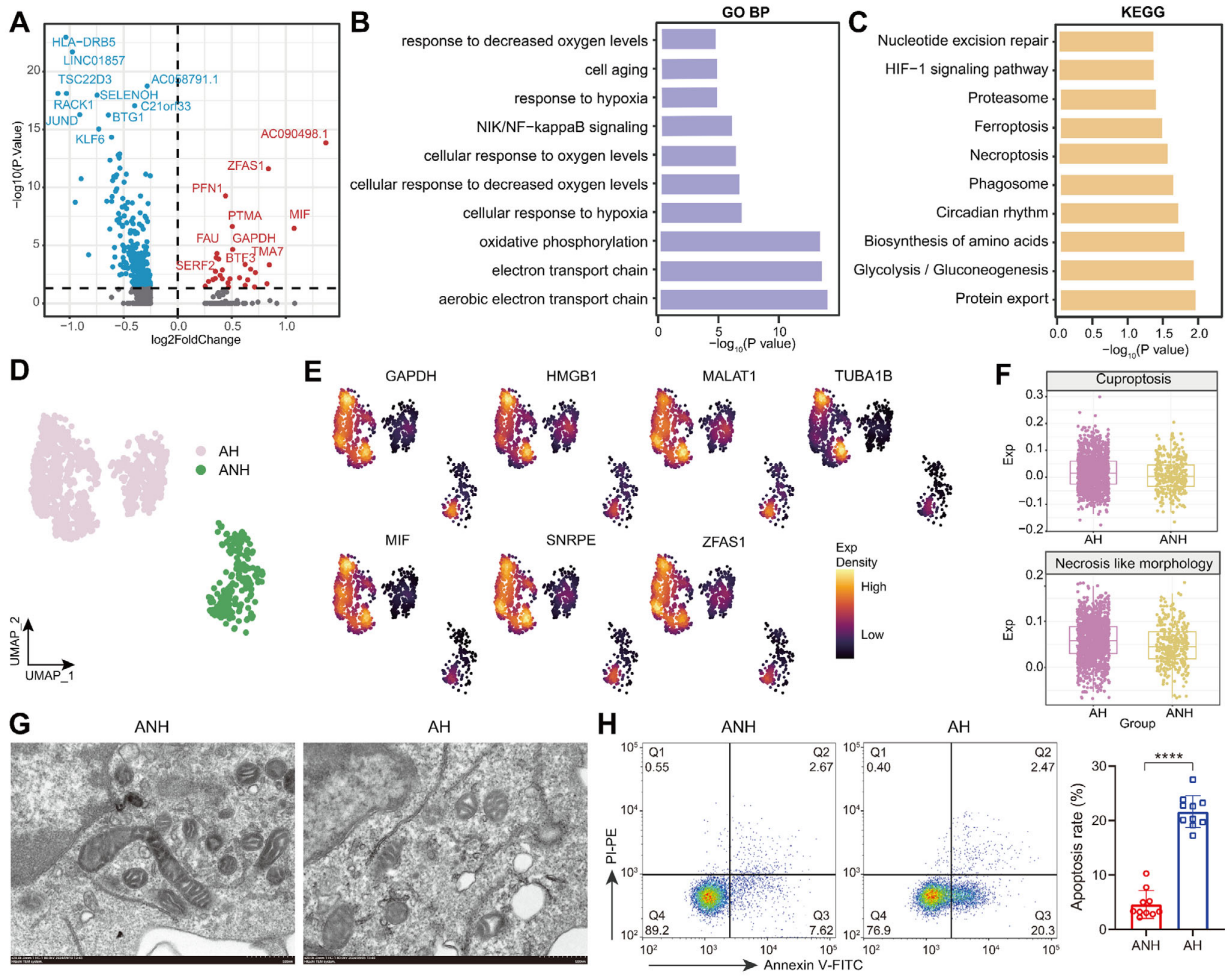
The gene expression pattern of CD9^+^ B cells in adenoid development. (A) The differentially expressed genes (DEGs) of CD9^+^ B cells in the AH and ANH groups. (B) Biological processes that were significantly enriched by DEGs of CD9^+^ B cells. (C) Signaling pathways that were significantly enriched by DEGs of CD9^+^ B cells. (D) Single‐cell atlas mapping the distribution of CD9^+^ B cells in AH and ANH tissues. (E) Density plot mapping the expression of PANoptosis‐related genes. (F) The boxplot showing that CD9^+^ B cells exhibited higher cell death‐related scores in the AH group compared with the ANH group. (G) Transmission electron microscope images showing the morphology of CD19^+^CD9^+^ B cells in the ANH and AH groups (scale bar, 500 nm). (H) Flow cytometry showing that the apoptotic proportion of CD19^+^CD9^+^ B cells in the AH group was higher than that in the ANH group (****p < 0.0001).

To understand the differences in cell abundance at various stages, we examined the gene expression patterns of CD9^+^ B cells in AH and ANH adenoid tissues. Compared with the ANH group, we found that ZFAS1, PFN1, and MIF were significantly upregulated in the AH group (Table S5, Figure 5A). Enrichment analysis revealed that the differentially expressed genes (DEGs) were significantly enriched in biological processes (BPs) related to cellular senescence, hypoxia, oxidative stress, and pathways associated with cell death (Figure 5B,C). Next, we visualized the dimensional reduction of CD9^+^ B cells (Figure 5D) and found that genes associated with cell death were predominantly expressed in the AH group (Figure 5E). Additionally, CD9^+^ B cells in the AH group exhibited morphological characteristics resembling necrosis and plaque formation, unlike those in the ANH group (Figure 5F). This was further confirmed by transmission electron microscope, which showed that CD19^+^CD9^+^ B cells in the AH group had more necrotic‐like features (such as rupture of the plasma membrane, swelling of the cytoplasm and organelles, and leakage of cellular contents) than those in the ANH group (Figure 5G). Flow cytometry also demonstrated a higher apoptotic proportion in CD19^+^CD9^+^ B cells in the AH group than that in the ANH group (Figure 5H).

These findings suggest that cell death may contribute to the reduced number of CD9^+^ B cells during adenoid development, although the precise regulatory mechanisms remain unclear.

### 2.6 Increased Abundance of Tfh Cells Promotes Adenoid Development and Is Associated With Decreased Abundance of CD9^+^ B Cells

**FIGURE 6:**
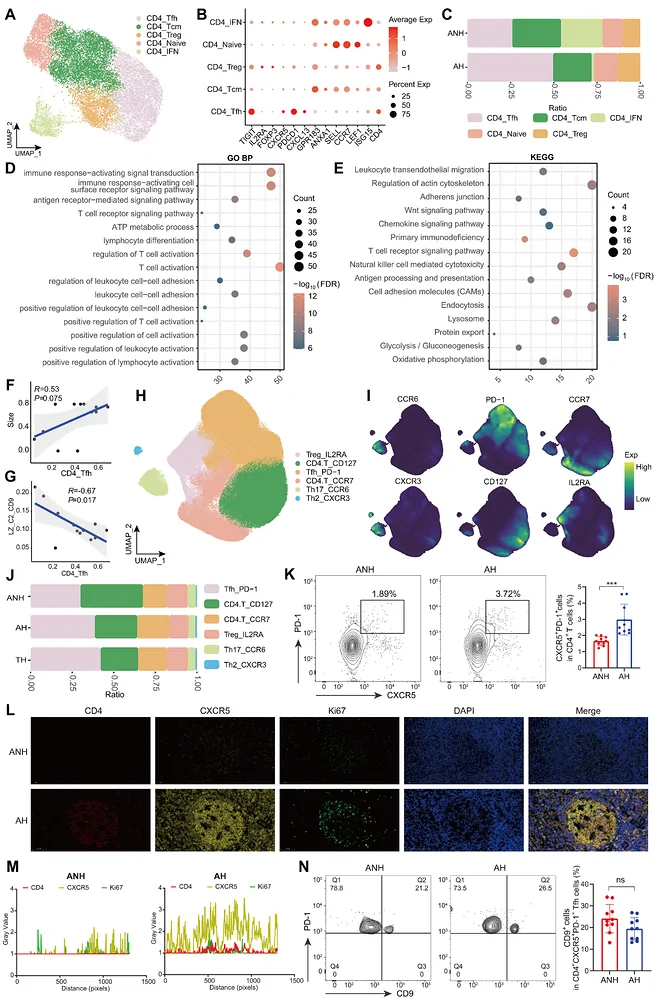
The role of increased abundance of Tfh cells in adenoid development. (A) Single‐cell atlas mapping CD4^+^ T subsets in adenoidal tissue. (B) Bubble plot showing marker expression of CD4^+^ T subsets. (C) Abundance ratio of CD4^+^ T cell subsets in AH and ANH groups. (D) Gene Ontology (GO) biological processes (BPs) that were significantly enriched in the Tfh cell subset. (E) Kyoto Encyclopedia of Genes and Genomes (KEGG) pathways that were significantly enriched in the Tfh cell subset. (F) The correlation diagram showing the relationship between the proportion of Tfh cells and adenoid size. (G) The correlation diagram showing that the abundance ratios of CD9^+^ B cells and Tfh cells were negatively correlated. (H) Single cell atlas showing CD4^+^ T cell subsets by CyToF mass spectrometry in AH, ANH, and tonsil (TH) tissues. (I) Density plot showing markers of CD4^+^ T cell subsets in ANH, AH, and TH tissues using CyToF. (J) Bar chart showing the abundance ratio of CD4^+^ T cell subsets in ANH, AH, and TH tissues measured by CyToF. (K) Flow cytometry showing that the abundance of CD4^+^CXCR5^+^PD‐1^+^ Tfh cells in the AH group was higher than that in the ANH group (***p < 0.001). (L, M) Multiple immunofluorescence showing the expression and distribution of Tfh cells in AH and ANH tissues (scale bar, 50 µm). (N) Flow cytometry showing the proportion of CD9^+^ Tfh cells in the AH and ANH groups (ns: not significant).

Since the interaction between Tfh and B cells is crucial for the formation and maintenance of the GC, understanding these cell interactions is key to uncovering the mechanisms behind GC regression. To this end, we identified CD4^+^ T cell subsets based on specific markers (Figure 6A) and distinguished Tfh cells (Figure 6B). In adenoid hypertrophy (AH), the abundance of Tfh cells was significantly higher compared with that in non‐hypertrophic adenoids (ANH) (Figure 6C). Enrichment analysis revealed that Tfh cells significantly impacted biological processes (BPs) related to T cell activation and immune responses, as well as T cell receptor signaling pathways (Figure 6D,E), indicating their essential role in immune regulation.

Correlation analysis showed a positive relationship between Tfh cell abundance and adenoid size, but it was not statistically significant (Figure 6F; R = 0.53 and p = 0.075). The positive correlation between Tfh cell abundance and adenoid size indicates that the development of adenoids might be related to the activity of Tfh cells. Tfh cell abundance had a significant negative relationship with CD9^+^ B cell abundance (Figure 6G; R = ‐0.67 and p = 0.017). The negative correlation between Tfh and CD9^+^ B cell abundance suggests that Tfh cells may influence the immune balance in adenoids through their interactions with CD9^+^ B cells.

Subsequently, we used CyToF to identify CD4^+^ T cell subsets in ANH, AN, and tonsil (TH) tissues (Figure 6H,I) and confirmed the single‐cell sequencing results, showing that Tfh cell abundance in AH was higher than in ANH (Table S6, Figure 6J). Flow cytometry further validated that the proportion of CD4^+^CXCR5^+^PD‐1^+^ T cells (Tfh cells) was significantly increased in AH compared with that in ANH (Figure 6K, Figure S3E). Multiplex immunofluorescence confirmed the increase of Tfh cells in AH tissues (Figure 6L,M), further supporting the interaction between Tfh and CD9^+^ B cells. This interaction may play a key role in regulating the immune microenvironment of the adenoids and promoting adenoid involution. Given that CD9^+^ Tfh cells have been attributed with the expansion of GCs [27], we detected the proportion of CD9^+^ Tfh cells in ANH and AN tissues using flow cytometry. Our results showed that the proportion of CD9^+^ Tfh cells was lower in AH than in ANH, but the difference was not statistically significant (Figure 6N, Figure S3E). In summary, these findings suggest that Tfh cells may be involved in adenoid development and could interact with CD9^+^ B cells, potentially influencing adenoid regression.

### 2.7 CD9^+^ B Cells May Regulate GC Regression by Inducing Tfh Cell Apoptosis

**FIGURE 7:**
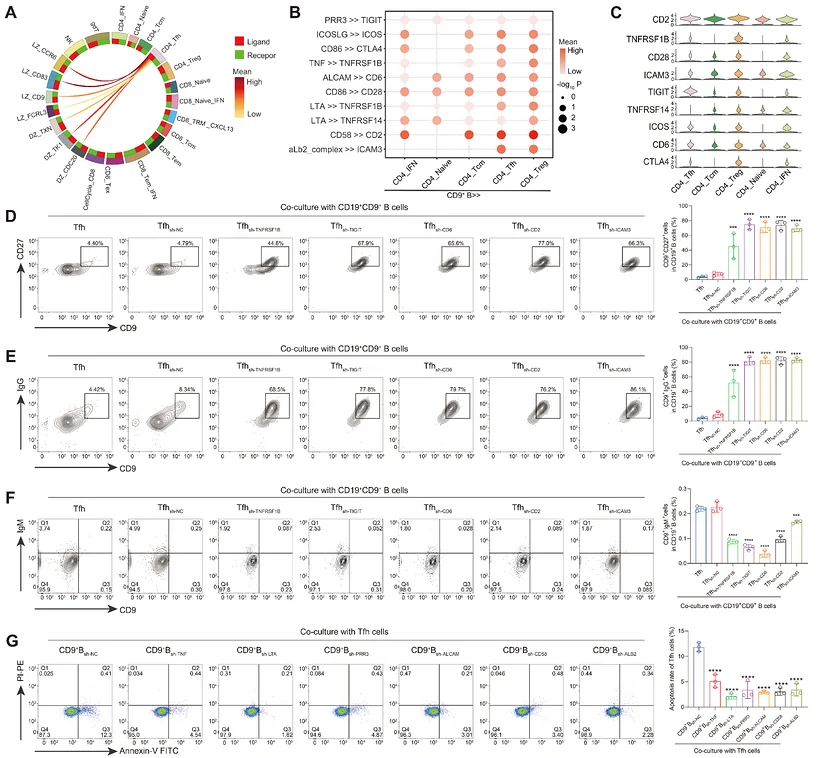
Exploration and validation of the interaction between CD9^+^ B cells and Tfh cells. (A) Circle diagram showing that there was significant intercellular communication interaction between Tfh cells and GC‐B cell subsets. (B) Bubble plot showing ligand‐receptor interactions between CD9^+^ B cells and CD4^+^ T cell subsets. (C) Violin plot showing the expression of corresponding receptor genes in CD4^+^ T cell subsets. (D) Flow cytometry showing that Tfh cells promoted the production of CD27 in CD9^+^ B cells after silencing ligand gene expression (***p < 0.001, ****p < 0.0001). (E) Flow cytometry showing that Tfh cells promoted the production of IgG in CD9^+^ B cells after silencing ligand gene expression (****p < 0.0001). (F) Flow cytometry showing that Tfh cells reduced the production of IgM in CD9^+^ B cells after silencing ligand gene expression (***p < 0.001, ****p < 0.0001). (G) Flow cytometry showing that CD9^+^ B cells suppressed Tfh cell apoptosis after silencing receptor gene expression (****p < 0.0001).

To further investigate the interaction between Tfh cells and CD9^+^ B cells, we conducted a cell communication analysis between T cell subsets and GC‐B cell subpopulations. We identified significant intercellular communication between Tfh cells and CD9^+^ B cells (Figure 7A). This analysis revealed several ligand‐receptor interactions between the two cell types, including LTA‐TNFRSF1B, ALCAM‐CD6, and CD58‐CD2 (Figure 7B). The expression of these receptor genes in the CD4^+^ T cell subset was validated (Figure 7C), further supporting the close connection between Tfh cells and CD9^+^ B cells.

To explore the relationship between CD9 expression and Tfh cell abundance, we compared Tfh cell populations in spleen, mesenteric lymph nodes, and inguinal lymph nodes of 6‐week‐old Cd9 knockout (CD9‐KO) and wild‐type (CD9‐WT) mice. No significant differences in Tfh cell abundance were observed between the two groups (Figure S2A–C). Subsequently, we knocked out the CD9 gene under immune conditions and monitored Tfh cell abundance at 0, 14, and 21 days; however, no significant differences were observed, suggesting that CD9 expression may not directly influence Tfh cell abundance (Figure S2D–F). Next, we silenced the expression of relevant ligands (such as TNFRSF1B, TIGIT, CD6, CD2, and ICAM3) in Tfh cells and co‐cultured them with CD9^+^ B cells. This significantly increased CD27 and IgG production and reduced IgM production in CD9^+^ B cells (Figure 7D–F, Figure S3F), indicating enhanced differentiation and maturation of CD9^+^ B cells. When we silenced the expression of corresponding receptors (TNF, LTA, PRR3, ALCAM, CD58, and ALB2) in CD9^+^ B cells and co‐cultured them with Tfh cells, we observed a marked inhibition of Tfh cell apoptosis (Figure 7G). These findings strongly suggest that CD9^+^ B cells may regulate GC degradation by inducing Tfh cell apoptosis, thereby promoting the natural involution of adenoids.

**FIGURE 8:**
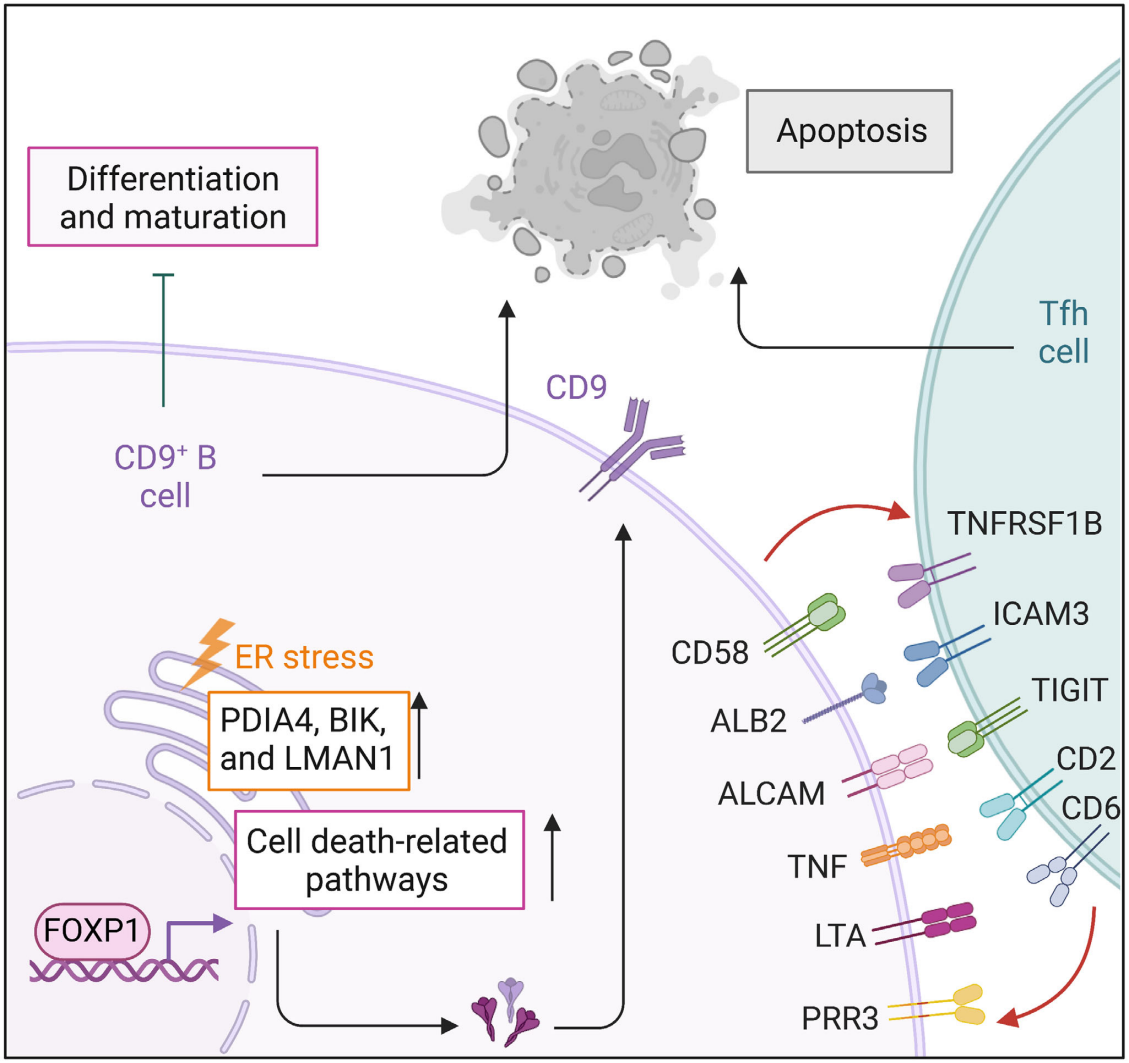
Graphical abstract showing the interaction between CD9^+^ B cells and Tfh cells.

In conclusion, these studies reveal the complex cellular communication between Tfh cells and CD9^+^ B cells (Figure 8). CD9^+^ B cells may influence the immune balance and natural regression of adenoids by regulating the survival and function of Tfh cells, offering new insights into the mechanisms of adenoid involution.

## 3 Discussion

B cells, particularly the number of GC‐B cells, are key factors determining the size of the adenoid. Through a comprehensive analysis of scRNA‐seq, flow cytometry, and CyToF, this study identified a unique CD9^+^ B cell subpopulation in the adenoid. The abundance of CD9^+^ B cells was negatively correlated with adenoid size and may be involved in regulating GC regression. During the development of GC, the differentiation of the CD9^+^ B cell subpopulation may be regulated by FOXP1. Additionally, ER stress in CD9^+^ B cells was significantly activated, and death‐related pathways were significantly enriched in CD9^+^ B cells in the AH group, suggesting that ER stress and necrosis may be among the reasons for the low abundance of CD9^+^ B cells. Cell communication analysis revealed significant ligand‐receptor interactions between Tfh cells and CD9^+^ B cells, indicating their close relationship in the processes of adenoid development and atrophy. Although the expression of CD9 does not directly affect the abundance of Tfh cells, CD9^+^ B cells may regulate GC regression by inducing Tfh cell apoptosis, thereby promoting the natural atrophy of the adenoid. These findings reveal the complex interactions between Tfh cells and CD9^+^ B cells, providing new insights into the regulatory mechanisms of the adenoid.

CD9 has often been considered one of the markers for Bregs [26, 28, 29]; however, recent studies indicate that CD9 is not a stable marker for Bregs [30]. In our study, the identified CD9^+^ B cell subpopulation expressed several markers associated with Bregs (such as CD24, CD38, and CD27), but its overall gene expression profile and function differ from previously reported CD9^+^ B cells. We found that the increased abundance of this subpopulation was negatively correlated with adenoid size, suggesting that it may play an important role in regulating GC regression. Notably, this study found that while CD9 expression may not directly affect Tfh cell abundance, the knockout of the Cd9 gene significantly inhibited the production of IgG1 antibodies in mice. This indicates that CD9 may play an important role in the interaction between B cells and Tfh cells and may also influence the immune function of B cells themselves, thereby affecting antibody production and the overall immune response. This finding provides new insights into the complex role of CD9 in immune regulation, highlighting its critical role in B cell function. Furthermore, we identified that FOXP1 may regulate the differentiation of the CD9^+^ B cell subpopulation. FOXP1 is a transcriptional repressor that binds to various promoters and enhancers through its forkhead DNA‐binding domain. It has been reported to play a coordinating role in the transition between cell proliferation and differentiation in various biological contexts, including myocytes [31], neurons [32], monocytes and macrophages [33], T cells [34], and stem cells [35]. In B cells, FOXP1 is critical for the survival, maintenance, and quiescence of peripheral mature B cells and is essential for the development of specific B cell subpopulations [36]. Previous studies have shown that FOXP1 negatively affects the development of GCs, and prolonged expression of FOXP1 may hinder the effective differentiation program necessary for GC formation [37]. Therefore, FOXP1 may regulate GC regression by influencing the differentiation of CD9^+^ B cells, but its specific mechanisms still require further investigation. To further distinguish the functional divergence between CD9^+^ B cells and Bregs, our future research will focus on precisely mapping the single‐cell multi‐omic profiles of these two populations; identifying the specific markers for each population to eliminate phenotypic overlap; and dissecting key transcription factors and signaling pathways that govern the distinct functional differentiation fates of the two populations.

Our research also revealed that CD9^+^ B cells exhibited significant activation of ER stress, with notable differences in gene expression between the ANH and AH groups, and the upregulated genes significantly activated pathways related to cell death. One of the primary causes of ER stress is hypoxia [38]. This stress can lead to two distinct outcomes: under acute conditions, it activates pro‐apoptotic programs that result in cell death, while under chronic and mild conditions, it may trigger pro‐survival responses [39]. Hypoxia plays a critical role in regulating the development, metabolism, and function of B cells [40]. Acute or severe hypoxia can lead to B cell dysfunction and cell death [41, 42, 43]. Therefore, hypoxia may be one of the factors causing CD9^+^ B cells to experience ER stress. Additionally, we observed that CD9^+^ B cells in the ANH group exhibited necrotic morphology compared with those in the AH group. This suggests that hypoxia and ER stress may work together to induce necrosis or apoptosis in CD9^+^ B cells, thereby affecting their function and regulatory role in the adenoids. These findings provide new insights into the role of CD9^+^ B cells in the adenoid immune microenvironment, particularly how they influence natural atrophy or dysfunction under stress conditions. However, the specific mechanisms warrant further investigation.

We also found that the abundance of Tfh cells was closely related to the abundance of CD9^+^ B cells and adenoid size. Tfh cells, located within B cell follicles, are crucial for the formation of GCs and the generation of high‐affinity B cells [44, 45]. Unlike previous studies [46], we found that the abundance of Tfh cells was positively correlated with adenoid size, suggesting that Tfh cells may play a more complex role in the immune regulation and volume changes of the adenoid. Through ligand‐receptor interaction analysis, we identified six pairs of potential ligand‐receptor pairs, with the ALCAM‐CD6 axis being particularly significant in the interactions between Tfh cells and CD9^+^ B cells. ALCAM is present in various cell types, CD6 is primarily expressed in T cells, particularly CD4^+^ T cells [47]. ALCAM's immunological function involves binding to CD6 and regulating immune processes like T cell activation, differentiation, and migration [48], and its interaction with CD6 is crucial for immune response regulation. In our study, the ALCAM‐CD6 axis may be one of the key pathways in the interactions between Tfh cells and CD9^+^ B cells, further indicating that Tfh cells and CD9^+^ B cells jointly regulate immune balance during the processes of adenoid development and atrophy.

This study reveals the complex pathways of interaction between B cells and Tfh cells, particularly their regulatory roles in the development and regression of the adenoid. However, there are some limitations to this research. First, the depth and coverage of single‐cell sequencing may be insufficient to fully capture rare subpopulations and dynamic changes. Second, the spatial heterogeneity within adenoid tissues and the dynamic changes in immune cells were not fully considered. Additionally, the complexity of the immune microenvironment was simplified, and results from in vitro experiments may differ from the in vivo environment. The small sample size may affect statistical significance and the assessment of individual differences. There is a lack of clinical validation, and the mechanisms discovered in this study have yet to be further validated in different patients or disease states. The inherent absence of adenoid tissue in rodents prevents direct in situ observation of the functional properties of CD9^+^ B cells in AH. Nevertheless, adoptive transfer assays of CD9^+^ B cells into suitable animal models provide a potential strategy to dissect the function of CD9^+^ B cells, generating preclinical evidence that supports the translational development of novel immune‐targeted intervention strategies for AH. Further optimization of humanized mouse models, particularly those engrafted with viable human adenoid tissue, will narrow the physiological divergence between experimental systems and human pathological conditions, accelerating clinical application.

## 4 Conclusions

In summary, our results demonstrate that during adenoid development, high abundance of Tfh cells inhibits the maturation and differentiation of CD9^+^ B cells; as adenoid development gradually improves, the number of CD9^+^ B cells increases significantly, thereby regulating GC regression by promoting Tfh cell apoptosis. These findings help fill in the gaps in the mechanisms regulating GC degeneration and eventual termination, and may help provide new insights into the treatment of diseases such as AH.

## 5 Experimental Section

### 5.1 Patients and Samples

A total of 18 samples, including 13 adenoid tissues and 5 tonsil samples from 13 patients, were involved in this study for scRNA‐seq. Adenoids were diagnosed by physical examination and fiber optic nasopharyngoscopy (using a 2.2‐mm, 0‐degree telescope, Olympus, Japan). Samples were grouped as previously described [49, 50]. AH (%) was quantified as the proportion of the adenoid obstruction area within the nasopharyngeal airway area as measured by nasoendoscopy, which represents the degree of adenoid hypertrophy. The calculation formula of AH (%) was as follows: AH (%) = adenoid obstruction area / nasopharyngeal airway area × 100%. Patients were divided into the ANH group (adenoid hypertrophy accounting for <50% of the nasopharyngeal space, n = 4) and the AH group (adenoid hypertrophy accounting for >50% of the nasopharyngeal space, n = 7). All samples were obtained with the consent from patients and appropriate approval from ethical committees (2022‐145) at The First Affiliated Hospital of Guangzhou Medical University.

### 5.2 Single‐Cell Dissociation

Adenoid tissues were surgically removed and kept in MACS Tissue Storage Solution (Miltenyi Biotec, Bergisch Gladbach, Germany) until processing. Briefly, tissue samples were first washed with sterile phosphate‐buffered saline (PBS) (Gibco, Waltham, MA, USA) and then minced into small pieces (approximately 1 mm^3^) on ice and homogenized in 1x PBS containing 2% fetal bovine serum (FBS) (Gibco). Samples were sieved through a 70‐µm cell strainer (BD Falcon, Franklin Lakes, NJ, USA), and centrifuged at 300 x g for 5 min. After washing with PBS containing 0.04% bovine serum albumin (BSA) (Sigma‐Aldrich, St. Louis, MO, USA), the cell pellets were re‐suspended in PBS containing 0.04% BSA and re‐filtered through a 40 µm cell strainer (BD Falcon). Dissociated single cells were then stained for viability assessment using Calcein‐AM cell‐permeant dye (Thermo Fisher Scientific, Waltham, MA, USA) and Draq7 (BD Biosciences, Franklin Lakes, NJ, USA).

#### 5.2.1 scRNA‐seq (10x Genomics)

scRNA‐seq was performed by NovelBio Co., Ltd (Jiangxi, China). scRNA‐Seq libraries were generated using the 10x Genomics Chromium Controller Instrument and Chromium Single Cell 3’ V2 Reagent Kits (10x Genomics, Pleasanton, CA, USA). Briefly, cells were concentrated to 1000 cells/µL, and approximately 10,000 cells were loaded into each channel to generate single‐cell Gel Bead‐In‐Emulsions (GEMs). After the reverse transcriptase (RT) step, GEMs were broken and barcoded cDNA was purified and amplified. The amplified barcoded cDNA was fragmented, A‐tailed, ligated with adaptors, and index PCR amplified. The final libraries were quantified using the Qubit High Sensitivity DNA assay (Thermo Fisher Scientific) and the size distribution for each library was determined using a High Sensitivity DNA chip on a Bioanalyzer 2200 (Agilent Technologies, Santa Clara, CA, USA). All libraries were sequenced as 150 bp paired‐end reads on the Illumina NovaSeq6000 sequencer (Illumina, San Diego, CA, USA).

#### 5.2.2 scRNA‐seq (BD)

Sequencing was performed with a BD Rhapsody system. Single‐cell capture was achieved by random distribution of a single‐cell suspension across >200,000 microwells via limited dilution. Beads with oligonucleotide barcodes were added to saturation, ensuring that each microwell contained a bead paired with a cell. Cell lysis buffer was added so that poly‐adenylated RNA molecules could hybridize to the beads. Beads were collected into a single tube for reverse transcription. Upon cDNA synthesis, each cDNA molecule was tagged on the 5′ end (that is, the 3′ end of an mRNA transcript) with a unique molecular identifier (UMI) and cell label indicating its cell of origin. Whole transcriptome libraries were prepared using the BD Rhapsody single‐cell whole‐transcriptome amplification workflow. In brief, second‐strand cDNA was synthesized, followed by ligation of the whole transcriptome amplification (WTA) adaptor for universal amplification. Eighteen cycles of PCR were used to amplify the adaptor‐ligated cDNA products. Sequencing libraries were prepared using random priming PCR of the whole‐transcriptome amplification products to enrich the 3′ end of the transcripts linked with the cell label and UMI. Sequencing libraries were quantified using a High Sensitivity DNA chip (Agilent) on a Bioanalyzer 2200 system and a Qubit High Sensitivity DNA assay (Thermo Fisher Scientific). The library for each sample was sequenced on a Novaseq6000 (Illumina) using a 150‐bp paired‐end run.

#### 5.2.3 scRNA‐seq Sample Inclusion and Exclusion Criteria

Only pediatric adenoid tissues were included for scRNA‐seq analysis, as adenoid development and physiological involution occur primarily during childhood and early adolescence. Adult adenoids were typically residual and highly involuted, lacking normal lymphoid follicles and GC structures; therefore, they were not suitable for studying adenoid immune ecology. For this reason, two adult samples (31 and 36 years old; Table S1) were excluded prior to analysis. All included samples exhibited intact lymphoid architecture and met standard scRNA‐seq quality requirements.

### 5.3 Differential Gene Expression Analysis

To identify differentially expressed genes (markers) for CD9^+^ B cells, the functions FindAllMarkers (multiple condition comparisons) and FindMarkers (two conditions) from the Seurat package were used with default parameters. Significant differentially expressed genes (markers) were selected as those with adjusted *p* values less than 0.05, average fold‐change larger than 1.5, and the percentage of cells with expression higher than 0.1.

### 5.4 Gene Set Enrichment Analysis

For gene enrichment analysis, Fisher's exact test was applied to calculate the p‐value for each gene set. Raw p‐values were adjusted for multiple hypothesis tests using the Benjamini & Hochberg method. Such enrichment analysis was applied to annotations including Gene Ontology (GO), Kyoto Encyclopedia of Genes and Genomes (KEGG), and customized gene sets. The Gene Ontology (GO) annotations were downloaded from the NCBI (http://www.ncbi.nlm.nih.gov/), the UniProt (http://www.uniprot.org/), and the Gene Ontology (http://www.geneontology.org/). KEGG annotation was purchased from the KEGG database (http://www.genome.ad.jp/kegg/). QuSAGE (v2.16.1) [51] analysis was conducted to characterize enriched pathways among the differentially expressed genes identified by the FindAllMarkers function as described above.

### 5.5 Pseudotime Analysis

Pseudotime analysis can reshape the change process of cells over time through the change trajectories between cells, and arrange each cell on the corresponding trajectory according to pseudotime, thereby predicting the differentiation and development trajectories of cells. In this study, the Monocle 2 [52] method was used to conduct a pseudotime analysis of the cell population of interest, reconstruct the developmental trajectory of the cell subpopulation, and visualize the expression changes of the gene of interest over time.

### 5.6 Gene Regulatory Network Analysis

The gene regulatory network (GRN) determines and maintains cell‐type‐specific transcriptional states, which were the foundation of cell morphology and function. In this study, GRN analysis was performed based on the pySCENIC method. pySCENIC [53] is a lightweight and fast python library based on SCENIC. SCENIC can reconstruct regulons (such as transcription factors and their target genes), evaluate the activity of these regulons in individual cells, and use these cellular activity patterns to find meaningful clusters of cells. The activity of these regulons was quantified via an enrichment score for the regulon's target genes (AUCell).

### 5.7 Cell Communication Analysis

CellPhoneDB 2 was a Python‐based computational analysis tool developed by Roser Vento‐Tormo et al. [54], which enables analysis of cell‐cell communication at the molecular level. A website version was also provided for analysis of a relatively small dataset (http://www.cellphonedb.org/). This procedure was followed by the annotation of membrane, secreted, and peripheral protein clusters at various time points. Mean and cell communication significance (*p* < 0.05) were determined based on the interaction and a normalized cell matrix obtained using Seurat normalization.

### 5.8 Single‐Cell RNA Statistical Analysis

We applied fastp (https://github.com/OpenGene/fastp) with default parameters to remove adaptor sequences and low‐quality reads. For the 10x Genomics scRNA‐seq data, the feature‐barcode matrices were obtained by aligning reads to the human genome (Ensemble version 91) using CellRanger (version 3.0.0). For the BD Rhapsody scRNA‐seq data, the clean data were mapped to the human genome (Ensemble version 91) utilizing STAR (version 2.5.2b) mapping with customized parameters from the UMI‐tools standard pipeline to obtain UMI counts for each sample. Cells containing over 200 expressed genes and with a mitochondrial UMI rate below 20% passed quality filtering, and mitochondrial genes were removed. To integrate cells from different platforms for unsupervised clustering, canonical correlation analysis (CCA) was used in the Seurat alignment workflow (version 3.1.4, https://satijalab.org/seurat/) [55]. Principal component analysis (PCA) was conducted using the 2000 genes with the highest variability, and the top 10 principal components were used for Uniform Manifold Approximation and Projection (UMAP) [56] visualization using an unsupervised graph‐based clustering method. Marker genes were calculated with the FindAllMarkers function from the Seurat R package (version 3.1.1) using the Wilcoxon Rank‐Sum Test algorithm and default parameters.

### 5.9 Multicolor Immunofluorescence Staining

Formalin‐fixed adenoid tissues were dehydrated, paraffin embedded and cut into 4 µm sections. After xylene deparaffinization and rehydration, sections were blocked with 10% rabbit serum or 3% BSA. Then, the sections were incubated overnight at 4°C with primary antibodies (anti‐CD19 antibody: 1:100, 14‐0194‐82, eBioscience, San Diego, CA, USA; Coralite Plus 488‐conjugated anti‐CD9 antibody: 1:50, CL488‐60232, Proteintech, Wuhan, China; anti‐Ki67 antibody: 1:50, ab15580, Abcam, Cambridge, MA, USA), followed with incubation of fluorescently labeled secondary antibodies (Alexa647 labeled anti‐Rat IgG H&L: 1:200, ab150167, Abcam; Alexa568 labeled anti‐Rabbit IgG H&L: 1:200, ab175470, Abcam) for 1 h at room temperature. Finally, nuclei were stained with DAPI staining solution (ab2285549, Abcam). Images were captured with a Zeiss LSM 510 inverted fluorescence microscope (Zeiss, Oberkochen, Germany).

### 5.10 Animals

Healthy female C57BL/6 mice (6 to 8 weeks) were purchased from the Guangdong Medical Laboratory Animal Center (Guangdong, China). Cd9 knockout (CD9‐KO) mice used in this study were designed and developed by Shanghai Model Organisms Center, Inc (Shanghai, China). The animals were housed in a temperature‐controlled environment under a 12/12‐h light/dark cycle, with access to food and water ad libitum, and under specific pathogen‐free conditions at the Animal Center of Guangzhou Medical University. All the mice were immunized subcutaneously with 50 µg of NP‐KLH (N5060‐5, Biosearch Technologies) in Imject Alum. Spleen, inguinal lymph nodes, and intestinal lymph nodes were harvested and processed for analysis at days 0, 14, and 21. This study was approved by the Ethics Committee of Guangzhou Medical University (20230322), and all procedures were conducted in accordance with the experimental animal guidelines of Guangzhou Medical University.

### 5.11 Flow Cytometry

Tissues were processed immediately after being obtained from patients. Briefly, the collected adenoid tissues were washed twice with sterile PBS and then cut into small pieces (<1 mm in diameter). Samples were then digested with 2 mL trypsin (Gibco, Cat: R001100) and 1 mL collagenase type I (0.25%) (07902, StemCell Technologies, Vancouver, BC, Canada) for 15 min on a shaker at 37°C. Digestion was neutralized by adding 8 mL sterile PBS, which was then filtered with 70 µm cell strainers. After centrifugation, cells were resuspended in PBS supplemented with 0.5% BSA and 0.1% sodium azide and stained with anti‐CD19‐FITC antibody (11‐0199‐42, eBioscience) and anti‐CD9‐PE antibody (12‐0098‐42, eBioscience) for 1 h at 4°C.

Single‐cell suspensions of mouse spleen, inguinal lymph nodes, and intestinal lymph nodes were prepared as described above. For murine GC‐B cells, cells were stained with anti‐CD19‐APC antibody (17‐0191‐82, eBioscience), anti‐FAS‐PE antibody (12‐0951‐81, eBioscience), and anti‐GL7‐Alexa488 antibody (53‐5902‐82, eBioscience). For murine Tfh cells, cells were incubated with anti‐CD4‐FITC antibody (11‐0049‐42, eBioscience), anti‐CXCR5‐PE antibody (145504, Biolegend, San Diego, CA, USA), and anti‐PD‐1‐APC (17‐9985‐82, eBioscience).

Flow cytometry was performed with CytoFLEX S flow cytometer (Beckman Coulter, Miami, FL, USA), and analyzed by FlowJo software (version 10, TreeStar, Ashland, OR, USA).

#### 5.11.1 Enzyme‐Linked Immunosorbent Assay (ELISA)

The whole blood was drawn from orbit before mice were sacrificed. After serum isolation, the concentrations of serum IgM and IgG1 were measured at 450 nm by using mouse IgM ELISA kit (E‐EL‐M3036, Elabscience, Wuhan, China) and IgG1 ELISA kit (E‐EL‐M3035, Elabscience). All the experimental steps were performed according to the kit instructions.

### 5.12 CD19^+^CD9^+^, CD19^+^CD9^−^, And Tfh Cell Sorting

To further determine the regulatory roles of CD19^+^CD9^+^ B cells, we separated CD19^+^CD9^+^ B cells, CD19^+^CD9^−^ B cells, and CD4^+^CXCR5^+^PD‐1^+^ T cells (Tfh cells) from adenoid cells using microbeads following the manufacturer's procedures. To enrich CD19^+^CD9^+^ B cells or CD19^+^CD9^−^ B cells, the single cell suspensions of adenoid tissues were processed with both human REAlease CD19 microbead kit (130‐117‐034, Miltenyi Biotech) and FITC‐labeled anti‐human CD9 antibody (312104, Biolegend) and anti‐FITC microbeads (130‐048‐701, Miltenyi Biotech). In addition, the cell suspensions were processed with human REAlease CD4 microbead kit (130‐117‐037, Miltenyi Biotech), PE‐labeled anti‐human CXCR5 antibody (356904, Biolegend) and anti‐PE microbeads (130‐048‐801, Miltenyi Biotech), and APC‐labeled anti‐CD279 (PD‐1) antibody (379207, Biolegend) and anti‐APC microbeads (130‐090‐855, Miltenyi Biotech) to enrich CD4^+^CXCR5^+^PD‐1^+^ Tfh cells. Cellular purity was detected with flow cytometer (CytoFLEX S, Beckman Coulter), only sorting purity more than 95% can be used for subsequent experiments.

#### 5.12.1 Reverse Transcription‐Quantitative PCR (RT‐qPCR)

Total RNA from CD19^+^CD9^+^ cells and CD19^+^CD9^−^ cells was extracted using the Invitrogen PureLink RNA kit (Invitrogen, Carlsbad, CA, USA) according to the manufacturer's protocol. cDNA was generated using the TransScript One‐Step gDNA Removal and cDNA Synthesis SuperMix Kit (TransGen Biotech, Beijing, China). Subsequently, qPCR with SYBR green was performed using the Agilent Mx3005P real‐time PCR system. Relative gene expression was calculated using the 2^−ΔΔCt^ method. The primer sequences of PDIA4, BIK, LMAN1, and GAPDH (internal reference) were shown in Table S7.

### 5.13 Western Blot

Protein was extracted using RIPA buffer, and protein concentration was detected with a BCA protein assay kit (Beyotime, Shanghai, China). The extracted protein was separated via SDS‐polyacrylamide gel electrophoresis and then transferred to PVDF membrane (Bio‐Rad, Hercules, CA, USA). The PVDF membrane was blocked and then incubated with primary antibodies, followed by peroxidase‐conjugated secondary antibodies. An enhanced chemiluminescence (ECL) detection system (Thermo Fisher Scientific) was used to visualize the protein bands. The antibodies used for Western blot were as follows: anti‐BIK (1:1000, AF6428, Affinity, Jiangsu, China), anti‐PDIA4 (1:1000, E‐AB‐52766, Elabscience), anti‐LMAN1 (1:500, Fnab04803, FineTest, Wuhan, China), anti‐FOXP1 (1:500, Fnab03206, FineTest), anti‐GAPDH (1:2500, ab9485, Abcam), and goat anti‐rabbit IgG H&L (HRP) (1:5000, ab6721, Abcam).

### 5.14 Cell Activation, Cell Transfection, and Apoptosis Assay

The obtained CD19^+^CD9^+^ B cells were cultivated with recombinant human CD40L (50 ng/mL, 310‐02, Peprotech, Rocky Hill, NJ, USA), CpG ODN 10101 (2.5 mg/mL, HY‐150217, MCE), and IL‐21 (50 ng/mL, 200–21, Peprotech). CD4^+^CXCR5^+^PD‐1^+^ T cells were induced and cultured by adding recombinant human IL‐6 (10 ng/mL, 200–06, Peprotech), ‌IL‐23 (10 ng/mL, PKSH033874, Elabscience), and ‌TGF‐β (5 ng/mL, 100–36E, Peprotech). For shRNA transfection, a mixture of 2 µg shRNA targeting FOXP1, TIGIT, LTA, CD6, PRR3, CD2, ICAM3, ALCAM, CD58, TNF, ABL2, TNFRSF1B, or negative control (sh‐NC) was separately added to 250 µL of serum‐free medium containing 5 µL Lipofectamine 3000 (L3000150, Invitrogen). The shRNA sequence information was shown in Table S8. After the solutions were mixed to form a shRNA/Lipofectamine 3000 complex, the complex was added to CD19^+^CD9^+^ B or CD4^+^CXCR5^+^PD‐1^+^ Tfh cells and incubated for 4–8 h. Then, the transfection solution was removed, and the cells were co‐cultured at a ratio of 1:1 by direct contact. After 48 h of co‐culture, CD19^+^CD9^+^ B cell subsets were analyzed for CD27, IgM, and IgG by flow cytometry. Tfh cell death was quantified by flow cytometry using a FITC Annexin V Apoptosis Detection Kit (556547, BD Pharmingen, San Diego, CA, USA). All the specific shRNA targeting different genes were designed and acquired from Synbio Technologies (Suzhou, China).

### 5.15 Mass Cytometry (CyTOF) and Data Analysis

Data from each sample were debarcoded from raw data using a doublet‐filtering scheme [57] with unique mass‐tagged barcodes. Each. fcs file generated from different batches was normalized through the bead normalization method [58]. Manually gated data were analyzed using FlowJo software to exclude debris, dead cells, and doublets, and to leave live and single immune cells. The UMAP algorithm was used to visualize the high‐dimensional data in two dimensions and show the distribution of each cluster, marker expression, and differences among each group or different sample types.

### 5.16 Preparation of Cell Suspensions

Adenoids and tonsil tissues were cut into small pieces and digested for 1 h at 37°C in RPMI containing 10% FBS and Collagenase type IV (2 mg/mL, Sigma Aldrich, St. Louis, MO, USA) + Hyaluronidase (250 µg/mL, Sigma Aldrich) + Deoxyribonuclease (20 µg/mL, Sigma Aldrich). Dissociated tissue soup was filtered through 70 µm cell strainer and lysed with ACK lysis buffer.

### 5.17 Antibodies

For mass cytometry analysis, purified antibodies were obtained from BioLegend, eBioscience, R&D systems (Minneapolis, MN, USA), and BD Biosciences using clones. Antibody labeling with the indicated metal tag was performed using the MaxPAR antibody Labeling kit (Fluidigm, Shanghai, China). Conjugated antibodies were titrated for optimal concentration before use.

### 5.18 Mass Cytometry Staining and Data Acquisition

Cells were washed once with 1xPBS and then stained with 100 µL of 250 nM cisplatin (Fluidigm) for 5 min on ice to exclude dead cells, and then incubated in Fc receptor blocking solution before being stained with surface antibodies cocktail for 30 min on ice. Cells were washed twice with FACS buffer (1xPBS + 0.5%BSA) and fixed in 200 µL of intercalation solution (Maxpar Fix and Perm Buffer containing 250 nM 191/193Ir, Fluidigm) overnight. After fixation, cells were washed once with FACS buffer and then perm buffer (eBioscience), stained with an intracellular antibody cocktail for 30 min on ice. Cells were washed and resuspended with deionized water, adding 20% EQ beads (Fluidigm), acquired on a mass cytometer (Helios, Fluidigm).

### 5.19 Statistical Analysis

Experimental data were expressed as the mean ± SE if appropriate. Unpaired Student's t‐test was applied for comparisons between the two groups, while one‐way analysis of variance (ANOVA) with Tukey's post hoc test was applied for multiple comparisons. The GraphPad Prism software (version 9.0.0; GraphPad, La Jolla, CA, USA) was used for data analysis. P < 0.05 was considered statistically significant.

## Author Contributions

**X.W.Z**., **Y.Z**. and **W.J.L**. designed the study; **W.J.L**., **L.J.S**., **M.Q.X**., **T.H.L**., **Y.X.C**., **G.C**., **Z.H.L**., **H.K.D**., **G.F.F**., **Z.M.F**., **Q.Z**., **F.P**., **S.X.Y**., **X.Y.Z**., **W.X.P**., **T.L.L**. and **Z.S.L**. performed the experiments; **Y.B.C**., **J.Y.L**., **B.X.P**., **Y.L**., **X.N**., **T.L**., **C.Y.H**., **K.M**. and **J.L.X**. analyzed the data; **W.J.L**., **L.J.S**., **X.N**., and **X.W.Z**. wrote the paper; **X.W.Z**., **Y.Z**. and **W.J.L**. revised the manuscript; all authors gave final approval of the version to be published.

## Funding

The study was supported by National Clinical Key Specialty Construction Project (2021‐2024), Guangzhou Medical Key Discipline Construction Project (2021‐2023), Autonomous Project of State Key Laboratory of Respiratory Disease (SKLRD‐Z‐202205), China Postdoctoral Science Foundation (2021M700947).

## Ethical Declaration

This study was approved by the Ethics Committee of The First Affiliated Hospital of Guangzhou Medical University (2022‐145), and complied with the ethical standards set out in the Declaration of Helsinki. Written informed consent was obtained from patients or their relatives for this study. All animal experiments were conducted in accordance with the guidelines of the Animal Review Committee of Guangzhou Medical University (20230322).

## Conflicts of Interest

The authors declare no conflicts of interest.

## Supporting information




**Supporting File**: advs77507‐sup‐0001‐SuppMat.docx.

## Data Availability

All data are available in the main text or the supplementary materials. scRNAseq data were submitted to the National Center for Biotechnology Information Gene Expression Omnibus database under accession numbers GSE262822.
