# Supplementary material for: CD9+ B Cells Induce T Follicular Helper Cell Apoptosis to Regulate Germinal Center Regression
Source: Adv Sci (Weinh). 2026 Aug 30:e02144. Online ahead of print. doi: 10.1002/advs.202502144 (PMC13526695; doi:10.1002/advs.202502144)
Supplement: Supplementary file 1 [file ADVS-9999-e02144-s001.docx]

**Supplementary information:**

Figure S1. Single-cell transcriptome analysis pipeline of this study. Tonsil and adenoid samples of adenoidal hypertrophy (AH) and adenoidal non-hypertrophy (ANH) were collected and used for 10x and BD single-cell sequencing.

Figure S2. Exploration of the correlation between CD9 expression and Tfh cell abundance. (A-C) Flow cytometry showing the effect of Cd9 knockout on the abundance ratio of Tfh cells in spleen (A), mesenteric lymph nodes (B), and inguinal lymph nodes (C) of mice. (D-F) Flow cytometry showing the effect of Cd9 knockout on the abundance ratio of Tfh cells in spleen (D), mesenteric lymph nodes (E), and inguinal lymph nodes (F) of mice under immune conditions (*P < 0.05).

Figure S3. The gate strategy of flow cytometry. (A) The gate strategy for CD19^+^ B cells and CD19^+^CD9^+^ B cells in human adenoid tissues. (B) The gate strategy for CD4^+^ / CD8^+^ T cells in human adenoid tissues. (C) The gate strategy for FOXP1^+^ cells and CD27^+^ cells in CD19^+^CD9^-^ B cells or CD19^+^CD9^+^ B cells. (D) The gate strategy for CD19^+^GL7^+^FAS^+^ GC-B cells in mouse inguinal and mesenteric lymph node tissues and spleen tissues. (E) The gate strategy for CD4^+^CXCR5^+^PD-1^+^ Tfh cells and CD9^+^ Tfh cells in human and mouse tissues. (F) The gate strategy for CD19^+^CD9^+^CD27^+^ B cells, CD19^+^CD9^+^IgG^+^ B cells, and CD19^+^CD9^+^IgM^+^ B cells in the co-culture system of CD19^+^CD9^+^ B cells with Tfh cells.

Figure S4. Raw western blot images for this study. (A) Raw images of PDIA4, BIK, LMAN1, and GAPDH expression corresponding to Figure 2K. (B) Raw images of FOXP1 and GAPDH expression corresponding to Figure 3K.

Table S1. Sample information included in single-cell RNA sequencing in this study.

| Patient | Sample ID | Tissue | Sex | Age | Group | Platform | Cells |
| --- | --- | --- | --- | --- | --- | --- | --- |
| 1 | PT-BD-S1 | Adenoid | Female | 4 | AH | BD | 3148 |
| 2 | T-BD-S1 | Tonsil | Male | 3 | / | BD | 4710 |
|  | PT-BD-S2 | Adenoid | Male | 3 | AH | BD | 3331 |
| 3 | PT-BD-S3 | Adenoid | Male | 2 | AH | BD | 3321 |
| 4 | PT-BD-S4 | Adenoid | Female | 11 | ANH | BD | 4584 |
| 5 | AH-CET | Adenoid | Female | 8 | AH | 10x Genomics | 6613 |
| 6 | AH-CY | Adenoid | Male | 4 | AH | 10x Genomics | 5046 |
| 7 | AH-CYX | Adenoid | Male | 3 | AH | 10x Genomics | 7269 |
| 8 | AH-XZA | Adenoid | Male | 8 | AH | 10x Genomics | 6690 |
| 9 | CTF-XYT | Adenoid | Male | 36 | / | 10x Genomics | 4413 |
| 10 | LLF-BTT | Tonsil | Male | 13 | / | 10x Genomics | 5281 |
|  | LLF-XYT | Adenoid | Male | 13 | ANH | 10x Genomics | 5308 |
| 11 | AH-HJS | Adenoid | Male | 5 | ANH | 10x Genomics | 4478 |
|  | TH-HJS | Tonsil | Male | 5 | / | 10x Genomics | 5173 |
| 12 | XIAN | Adenoid | Female | 31 | / | 10x Genomics | 5218 |
|  | BIAN | Tonsil | Female | 31 | / | 10x Genomics | 6085 |
| 13 | LJH-XYT | Adenoid | Male | 15 | ANH | 10x Genomics | 6997 |
|  | LJH-BTT | Tonsil | Male | 15 | / | 10x Genomics | 4076 |

Abbreviations: AH, adenoid hypertrophy; ANH, adenoid non-hypertrophy.

Table S2. Markers of CD9^+^ B cell subsets.

| p_val | avg_log2FC | pct.1 | pct.2 | p_val_adj | cluster | gene |
| --- | --- | --- | --- | --- | --- | --- |
| 0 | 1.265968329 | 0.667 | 0.326 | 0 | LZ_CD9 | SUGCT |
| 5.09E-157 | 1.149871164 | 0.59 | 0.377 | 9.17E-153 | LZ_CD9 | KMT2A |
| 1.72E-279 | 1.096314812 | 0.85 | 0.644 | 3.11E-275 | LZ_CD9 | ISG20 |
| 0 | 1.088339369 | 0.933 | 0.801 | 0 | LZ_CD9 | EZR |
| 1.06E-186 | 1.074533374 | 0.546 | 0.295 | 1.90E-182 | LZ_CD9 | FOXP1 |
| 3.29E-260 | 1.054213708 | 0.243 | 0.054 | 5.92E-256 | LZ_CD9 | COL9A3 |
| 4.54E-202 | 1.005707241 | 0.278 | 0.082 | 8.19E-198 | LZ_CD9 | TP53INP1 |
| 1.21E-227 | 1.004543582 | 0.812 | 0.568 | 2.18E-223 | LZ_CD9 | CXCR4 |
| 1.31E-121 | 0.999737914 | 0.504 | 0.323 | 2.37E-117 | LZ_CD9 | CD27 |
| 1.30E-50 | 0.978235162 | 0.457 | 0.351 | 2.34E-46 | LZ_CD9 | JUND |
| 3.94E-156 | 0.959712193 | 0.287 | 0.104 | 7.10E-152 | LZ_CD9 | RASSF6 |
| 2.12E-120 | 0.931288819 | 0.218 | 0.077 | 3.83E-116 | LZ_CD9 | PEG10 |
| 3.17E-159 | 0.924869666 | 0.413 | 0.193 | 5.71E-155 | LZ_CD9 | MME |
| 2.01E-151 | 0.919700228 | 0.3 | 0.116 | 3.63E-147 | LZ_CD9 | SH3TC1 |
| 5.34E-181 | 0.917808597 | 0.783 | 0.624 | 9.61E-177 | LZ_CD9 | C4orf3 |
| 8.04E-122 | 0.884516449 | 0.562 | 0.376 | 1.45E-117 | LZ_CD9 | ORAI2 |
| 3.81E-133 | 0.866780009 | 0.351 | 0.159 | 6.86E-129 | LZ_CD9 | SLC2A5 |
| 5.13E-176 | 0.859297262 | 0.798 | 0.59 | 9.25E-172 | LZ_CD9 | JCHAIN |
| 9.69E-151 | 0.847425766 | 0.631 | 0.387 | 1.75E-146 | LZ_CD9 | AICDA |
| 6.32E-63 | 0.830408217 | 0.199 | 0.094 | 1.14E-58 | LZ_CD9 | LGALS1 |
| 9.65E-169 | 0.813826908 | 0.251 | 0.077 | 1.74E-164 | LZ_CD9 | HRK |
| 5.80E-111 | 0.810357949 | 0.59 | 0.404 | 1.04E-106 | LZ_CD9 | SEC14L1 |
| 3.88E-104 | 0.805514666 | 0.435 | 0.251 | 6.99E-100 | LZ_CD9 | WDR66 |
| 7.50E-91 | 0.799460092 | 0.268 | 0.122 | 1.35E-86 | LZ_CD9 | RUBCNL |
| 4.72E-83 | 0.784918953 | 0.252 | 0.119 | 8.50E-79 | LZ_CD9 | RGCC |
| 1.20E-113 | 0.783361519 | 0.19 | 0.063 | 2.17E-109 | LZ_CD9 | KANK2 |
| 9.68E-120 | 0.781876364 | 0.595 | 0.409 | 1.75E-115 | LZ_CD9 | SUSD3 |
| 1.30E-103 | 0.781542168 | 0.55 | 0.352 | 2.35E-99 | LZ_CD9 | BACH2 |
| 7.25E-170 | 0.774300981 | 0.916 | 0.804 | 1.31E-165 | LZ_CD9 | LTB |
| 8.86E-131 | 0.768850106 | 0.423 | 0.214 | 1.60E-126 | LZ_CD9 | TAGAP |
| 1.72E-166 | 0.756965426 | 0.135 | 0.025 | 3.10E-162 | LZ_CD9 | BMP7 |
| 1.36E-75 | 0.744043946 | 0.399 | 0.258 | 2.45E-71 | LZ_CD9 | IFT57 |
| 1.44E-105 | 0.728024498 | 0.67 | 0.503 | 2.59E-101 | LZ_CD9 | DAAM1 |
| 6.85E-101 | 0.727890312 | 0.605 | 0.422 | 1.23E-96 | LZ_CD9 | IL4R |
| 1.31E-92 | 0.726031035 | 0.333 | 0.173 | 2.36E-88 | LZ_CD9 | CDK13 |
| 2.56E-67 | 0.718734458 | 0.464 | 0.314 | 4.61E-63 | LZ_CD9 | TSC22D3 |
| 2.35E-87 | 0.718528412 | 0.543 | 0.387 | 4.24E-83 | LZ_CD9 | BLOC1S6 |
| 1.31E-72 | 0.707686112 | 0.351 | 0.207 | 2.35E-68 | LZ_CD9 | MFHAS1 |
| 1.39E-102 | 0.705710788 | 0.604 | 0.448 | 2.51E-98 | LZ_CD9 | LSM10 |
| 2.72E-80 | 0.69001258 | 0.203 | 0.086 | 4.90E-76 | LZ_CD9 | RRBP1 |
| 1.15E-61 | 0.688780383 | 0.518 | 0.379 | 2.08E-57 | LZ_CD9 | RACK1 |
| 5.16E-75 | 0.686519125 | 0.354 | 0.208 | 9.30E-71 | LZ_CD9 | CHMP7 |
| 5.08E-34 | 0.679980279 | 0.789 | 0.727 | 9.15E-30 | LZ_CD9 | IGLC2 |
| 3.57E-108 | 0.67301045 | 0.234 | 0.091 | 6.43E-104 | LZ_CD9 | SYBU |
| 1.74E-86 | 0.67035536 | 0.604 | 0.464 | 3.14E-82 | LZ_CD9 | PPP2R5C |
| 7.21E-78 | 0.66804151 | 0.576 | 0.428 | 1.30E-73 | LZ_CD9 | NCOA3 |
| 5.59E-138 | 0.662331757 | 0.165 | 0.043 | 1.01E-133 | LZ_CD9 | USP53 |
| 7.52E-178 | 0.656589656 | 0.962 | 0.915 | 1.35E-173 | LZ_CD9 | ATP5L |
| 7.14E-91 | 0.650565996 | 0.664 | 0.514 | 1.29E-86 | LZ_CD9 | BCL7A |
| 8.23E-66 | 0.649455254 | 0.465 | 0.317 | 1.48E-61 | LZ_CD9 | DTX1 |
| 8.40E-86 | 0.644044198 | 0.659 | 0.535 | 1.51E-81 | LZ_CD9 | TPD52 |
| 3.81E-75 | 0.643946798 | 0.472 | 0.317 | 6.87E-71 | LZ_CD9 | ATM |
| 1.77E-86 | 0.643831713 | 0.276 | 0.133 | 3.19E-82 | LZ_CD9 | ST14 |
| 3.59E-65 | 0.641236397 | 0.336 | 0.196 | 6.47E-61 | LZ_CD9 | PIM2 |
| 8.91E-83 | 0.64035167 | 0.488 | 0.323 | 1.61E-78 | LZ_CD9 | TMEM156 |
| 8.72E-70 | 0.635836979 | 0.754 | 0.676 | 1.57E-65 | LZ_CD9 | DYNLL1 |
| 6.30E-74 | 0.628276377 | 0.238 | 0.112 | 1.14E-69 | LZ_CD9 | PRDM15 |
| 8.22E-48 | 0.624543438 | 0.389 | 0.274 | 1.48E-43 | LZ_CD9 | MSI2 |
| 3.71E-59 | 0.621291861 | 0.269 | 0.15 | 6.69E-55 | LZ_CD9 | RNF144B |
| 2.32E-120 | 0.617305718 | 0.237 | 0.086 | 4.18E-116 | LZ_CD9 | SLC30A4 |
| 2.24E-42 | 0.617205882 | 0.326 | 0.223 | 4.04E-38 | LZ_CD9 | ARID1B |
| 5.76E-55 | 0.615379199 | 0.402 | 0.272 | 1.04E-50 | LZ_CD9 | RNGTT |
| 2.62E-53 | 0.613301215 | 0.576 | 0.455 | 4.73E-49 | LZ_CD9 | PLEKHA2 |
| 2.67E-63 | 0.611280577 | 0.58 | 0.457 | 4.81E-59 | LZ_CD9 | SYNE2 |
| 2.88E-50 | 0.61077286 | 0.323 | 0.213 | 5.19E-46 | LZ_CD9 | USP34 |
| 7.84E-94 | 0.597192783 | 0.693 | 0.566 | 1.41E-89 | LZ_CD9 | RHOH |
| 4.53E-27 | 0.591359844 | 0.168 | 0.101 | 8.16E-23 | LZ_CD9 | NOL9 |
| 2.17E-56 | 0.590706858 | 0.315 | 0.192 | 3.92E-52 | LZ_CD9 | STK40 |
| 1.03E-31 | 0.584864765 | 0.437 | 0.354 | 1.85E-27 | LZ_CD9 | KLF6 |
| 3.69E-120 | 0.583492142 | 0.201 | 0.065 | 6.64E-116 | LZ_CD9 | ANK1 |
| 9.06E-132 | 0.583231575 | 0.169 | 0.046 | 1.63E-127 | LZ_CD9 | LINC02397 |
| 5.27E-136 | 0.582832155 | 0.936 | 0.881 | 9.49E-132 | LZ_CD9 | CD79B |
| 9.91E-60 | 0.582488676 | 0.515 | 0.397 | 1.79E-55 | LZ_CD9 | MAP1LC3B |
| 3.90E-58 | 0.58050527 | 0.258 | 0.141 | 7.02E-54 | LZ_CD9 | DNAJB2 |
| 1.77E-50 | 0.579416634 | 0.566 | 0.454 | 3.20E-46 | LZ_CD9 | RCSD1 |
| 2.77E-47 | 0.577777839 | 0.415 | 0.296 | 5.00E-43 | LZ_CD9 | DSTN |
| 7.44E-67 | 0.569785624 | 0.292 | 0.156 | 1.34E-62 | LZ_CD9 | BCL2L11 |
| 1.55E-63 | 0.564818547 | 0.514 | 0.377 | 2.80E-59 | LZ_CD9 | BLK |
| 5.31E-28 | 0.564499555 | 0.328 | 0.246 | 9.56E-24 | LZ_CD9 | PIM1 |
| 5.86E-54 | 0.559573987 | 0.479 | 0.364 | 1.06E-49 | LZ_CD9 | AES |
| 1.00E-45 | 0.55921422 | 0.241 | 0.14 | 1.80E-41 | LZ_CD9 | MGAT1 |
| 1.51E-44 | 0.557984653 | 0.294 | 0.183 | 2.72E-40 | LZ_CD9 | ELOB |
| 2.50E-44 | 0.55451963 | 0.354 | 0.247 | 4.50E-40 | LZ_CD9 | EHMT1 |
| 1.05E-40 | 0.551027839 | 0.136 | 0.065 | 1.89E-36 | LZ_CD9 | CD9 |
| 4.24E-125 | 0.550213761 | 0.916 | 0.866 | 7.65E-121 | LZ_CD9 | LIMD2 |
| 2.37E-50 | 0.543711141 | 0.492 | 0.375 | 4.27E-46 | LZ_CD9 | RBM38 |
| 1.15E-75 | 0.543306687 | 0.477 | 0.303 | 2.07E-71 | LZ_CD9 | 1-Mar |
| 2.03E-57 | 0.542673115 | 0.327 | 0.198 | 3.65E-53 | LZ_CD9 | DNASE1 |
| 9.82E-76 | 0.541372665 | 0.654 | 0.515 | 1.77E-71 | LZ_CD9 | NEIL1 |
| 7.66E-55 | 0.540818286 | 0.445 | 0.311 | 1.38E-50 | LZ_CD9 | ST6GAL1 |
| 1.66E-52 | 0.53804521 | 0.319 | 0.197 | 3.00E-48 | LZ_CD9 | AFF3 |
| 1.71E-17 | 0.537116758 | 0.171 | 0.118 | 3.08E-13 | LZ_CD9 | PTMS |
| 1.45E-227 | 0.533849467 | 0.985 | 0.964 | 2.62E-223 | LZ_CD9 | OAZ1 |
| 1.77E-22 | 0.533253908 | 0.383 | 0.301 | 3.19E-18 | LZ_CD9 | TXNIP |
| 2.32E-106 | 0.533141023 | 0.84 | 0.727 | 4.17E-102 | LZ_CD9 | IRF8 |
| 1.47E-29 | 0.532625106 | 0.187 | 0.113 | 2.66E-25 | LZ_CD9 | ALDH5A1 |
| 2.05E-59 | 0.532011667 | 0.245 | 0.127 | 3.69E-55 | LZ_CD9 | NOP53 |
| 2.66E-37 | 0.531415658 | 0.391 | 0.289 | 4.80E-33 | LZ_CD9 | APLP2 |
| 8.37E-24 | 0.530987827 | 0.316 | 0.242 | 1.51E-19 | LZ_CD9 | TBC1D1 |
| 2.27E-31 | 0.530342405 | 0.509 | 0.443 | 4.09E-27 | LZ_CD9 | NEAT1 |
| 5.32E-46 | 0.530252972 | 0.477 | 0.366 | 9.59E-42 | LZ_CD9 | CD38 |
| 4.94E-49 | 0.528388653 | 0.24 | 0.135 | 8.90E-45 | LZ_CD9 | SLC35E3 |
| 1.47E-42 | 0.527460276 | 0.541 | 0.437 | 2.66E-38 | LZ_CD9 | SMAP2 |
| 5.54E-133 | 0.527201799 | 0.94 | 0.912 | 9.98E-129 | LZ_CD9 | CD37 |
| 2.75E-18 | 0.526081127 | 0.286 | 0.223 | 4.95E-14 | LZ_CD9 | RGS2 |
| 4.73E-46 | 0.524958151 | 0.39 | 0.275 | 8.52E-42 | LZ_CD9 | HSH2D |
| 5.52E-174 | 0.521338683 | 0.987 | 0.972 | 9.96E-170 | LZ_CD9 | SERF2 |
| 5.88E-40 | 0.521031066 | 0.324 | 0.223 | 1.06E-35 | LZ_CD9 | GABPB1 |
| 9.96E-44 | 0.519378728 | 0.283 | 0.174 | 1.79E-39 | LZ_CD9 | RAPGEF1 |
| 1.78E-18 | 0.517963449 | 0.192 | 0.132 | 3.21E-14 | LZ_CD9 | JUN |
| 7.63E-50 | 0.517131549 | 0.254 | 0.146 | 1.38E-45 | LZ_CD9 | COBLL1 |
| 2.16E-29 | 0.516164473 | 0.336 | 0.255 | 3.89E-25 | LZ_CD9 | PHAX |
| 1.33E-50 | 0.515524788 | 0.454 | 0.335 | 2.39E-46 | LZ_CD9 | JMJD1C |
| 8.64E-51 | 0.512915159 | 0.191 | 0.097 | 1.56E-46 | LZ_CD9 | KIF3A |
| 1.35E-42 | 0.511292667 | 0.451 | 0.346 | 2.44E-38 | LZ_CD9 | CYTIP |
| 3.19E-50 | 0.508576481 | 0.184 | 0.089 | 5.74E-46 | LZ_CD9 | LINC01857 |
| 6.07E-36 | 0.501035947 | 0.214 | 0.128 | 1.09E-31 | LZ_CD9 | ZNF101 |
| 4.74E-25 | 0.500383921 | 0.408 | 0.329 | 8.54E-21 | LZ_CD9 | PRKCB |
| 2.28E-36 | 0.499822861 | 0.189 | 0.107 | 4.12E-32 | LZ_CD9 | CSNK1E |
| 6.24E-83 | 0.499157556 | 0.785 | 0.699 | 1.13E-78 | LZ_CD9 | TCEA1 |
| 2.57E-60 | 0.498599934 | 0.222 | 0.108 | 4.63E-56 | LZ_CD9 | C16orf74 |
| 3.40E-35 | 0.495313959 | 0.298 | 0.204 | 6.12E-31 | LZ_CD9 | C12orf49 |
| 1.83E-97 | 0.492614299 | 0.928 | 0.841 | 3.30E-93 | LZ_CD9 | TCL1A |
| 1.02E-58 | 0.48958774 | 0.223 | 0.109 | 1.84E-54 | LZ_CD9 | SELENOH |
| 1.00E-41 | 0.488672914 | 0.466 | 0.355 | 1.81E-37 | LZ_CD9 | GADD45B |
| 5.47E-20 | 0.487292649 | 0.406 | 0.346 | 9.85E-16 | LZ_CD9 | ZFP36L2 |
| 4.58E-66 | 0.483575522 | 0.786 | 0.684 | 8.25E-62 | LZ_CD9 | POU2AF1 |
| 4.61E-33 | 0.480492904 | 0.36 | 0.265 | 8.31E-29 | LZ_CD9 | AIM2 |
| 1.65E-46 | 0.478730869 | 0.291 | 0.176 | 2.97E-42 | LZ_CD9 | RTRAF |
| 2.52E-38 | 0.473244076 | 0.381 | 0.274 | 4.53E-34 | LZ_CD9 | HDAC7 |
| 1.55E-28 | 0.472960499 | 0.424 | 0.333 | 2.79E-24 | LZ_CD9 | TCF3 |
| 8.80E-33 | 0.471697508 | 0.315 | 0.222 | 1.59E-28 | LZ_CD9 | PHF3 |
| 4.97E-46 | 0.471459747 | 0.401 | 0.278 | 8.95E-42 | LZ_CD9 | YPEL3 |
| 2.65E-54 | 0.470987291 | 0.271 | 0.151 | 4.77E-50 | LZ_CD9 | SELENOF |
| 3.90E-19 | 0.469750511 | 0.954 | 0.965 | 7.04E-15 | LZ_CD9 | IGKC |
| 4.20E-60 | 0.469663207 | 0.658 | 0.566 | 7.57E-56 | LZ_CD9 | STK17A |
| 2.78E-32 | 0.468114946 | 0.351 | 0.263 | 5.02E-28 | LZ_CD9 | DEGS1 |
| 1.82E-52 | 0.468046435 | 0.668 | 0.591 | 3.29E-48 | LZ_CD9 | LAMTOR5 |
| 5.92E-40 | 0.466433106 | 0.629 | 0.56 | 1.07E-35 | LZ_CD9 | FNBP1 |
| 3.81E-38 | 0.464998625 | 0.472 | 0.366 | 6.86E-34 | LZ_CD9 | DCAF12 |
| 2.41E-35 | 0.464666196 | 0.377 | 0.279 | 4.34E-31 | LZ_CD9 | BOD1L1 |
| 3.78E-40 | 0.463723411 | 0.523 | 0.415 | 6.82E-36 | LZ_CD9 | MGEA5 |
| 2.83E-22 | 0.463540852 | 0.444 | 0.373 | 5.10E-18 | LZ_CD9 | STAG3 |
| 6.74E-35 | 0.462403769 | 0.514 | 0.422 | 1.21E-30 | LZ_CD9 | MAP4K4 |
| 1.78E-37 | 0.461611689 | 0.264 | 0.169 | 3.20E-33 | LZ_CD9 | SNTA1 |
| 3.14E-34 | 0.461227173 | 0.529 | 0.432 | 5.66E-30 | LZ_CD9 | ETS1 |
| 1.20E-37 | 0.461134202 | 0.588 | 0.504 | 2.16E-33 | LZ_CD9 | BPTF |
| 1.68E-36 | 0.460474335 | 0.573 | 0.478 | 3.03E-32 | LZ_CD9 | S100A10 |
| 6.72E-50 | 0.459763985 | 0.67 | 0.57 | 1.21E-45 | LZ_CD9 | SEL1L3 |
| 6.33E-44 | 0.458861591 | 0.495 | 0.386 | 1.14E-39 | LZ_CD9 | SNX2 |
| 1.82E-39 | 0.457439389 | 0.605 | 0.526 | 3.28E-35 | LZ_CD9 | EIF3A |
| 1.73E-36 | 0.455530142 | 0.425 | 0.32 | 3.12E-32 | LZ_CD9 | PPM1K |
| 7.64E-27 | 0.452238836 | 0.129 | 0.068 | 1.38E-22 | LZ_CD9 | IGHA2 |
| 3.16E-24 | 0.447975603 | 0.218 | 0.145 | 5.69E-20 | LZ_CD9 | BICD2 |
| 1.99E-34 | 0.446164957 | 0.36 | 0.263 | 3.59E-30 | LZ_CD9 | TMEM134 |
| 5.25E-26 | 0.444155086 | 0.216 | 0.142 | 9.46E-22 | LZ_CD9 | MLXIP |
| 2.27E-47 | 0.444045876 | 0.213 | 0.113 | 4.09E-43 | LZ_CD9 | SINHCAF |
| 2.02E-41 | 0.443015683 | 0.188 | 0.1 | 3.64E-37 | LZ_CD9 | KCNN3 |
| 5.35E-30 | 0.442843033 | 0.375 | 0.292 | 9.64E-26 | LZ_CD9 | CUL3 |
| 2.28E-27 | 0.440587793 | 0.339 | 0.249 | 4.11E-23 | LZ_CD9 | SLC15A4 |
| 8.89E-64 | 0.439419671 | 0.738 | 0.645 | 1.60E-59 | LZ_CD9 | BCAS4 |
| 8.85E-30 | 0.437966042 | 0.29 | 0.2 | 1.60E-25 | LZ_CD9 | RRM2B |
| 3.99E-52 | 0.436740238 | 0.122 | 0.048 | 7.19E-48 | LZ_CD9 | ELOA |
| 1.61E-168 | 0.43639847 | 0.997 | 0.995 | 2.89E-164 | LZ_CD9 | ACTG1 |
| 5.57E-40 | 0.435828186 | 0.133 | 0.063 | 1.00E-35 | LZ_CD9 | GADD45A |
| 3.79E-22 | 0.43400962 | 0.239 | 0.17 | 6.82E-18 | LZ_CD9 | PEX5 |
| 8.13E-45 | 0.432770118 | 0.393 | 0.268 | 1.46E-40 | LZ_CD9 | YPEL5 |
| 2.28E-51 | 0.431987511 | 0.21 | 0.106 | 4.12E-47 | LZ_CD9 | PLEKHG1 |
| 3.62E-44 | 0.431433461 | 0.217 | 0.119 | 6.52E-40 | LZ_CD9 | MCUB |
| 5.35E-39 | 0.430993674 | 0.286 | 0.179 | 9.65E-35 | LZ_CD9 | SEM1 |
| 7.36E-43 | 0.430869553 | 0.625 | 0.533 | 1.33E-38 | LZ_CD9 | GMFG |
| 2.15E-29 | 0.429623125 | 0.631 | 0.566 | 3.88E-25 | LZ_CD9 | SRSF9 |
| 1.93E-24 | 0.428005288 | 0.385 | 0.314 | 3.48E-20 | LZ_CD9 | ZCCHC10 |
| 9.54E-28 | 0.427963896 | 0.545 | 0.481 | 1.72E-23 | LZ_CD9 | MTPN |
| 5.01E-32 | 0.425625718 | 0.306 | 0.213 | 9.02E-28 | LZ_CD9 | BLCAP |
| 2.70E-31 | 0.423608933 | 0.431 | 0.346 | 4.87E-27 | LZ_CD9 | SMIM20 |
| 8.15E-26 | 0.422048888 | 0.392 | 0.306 | 1.47E-21 | LZ_CD9 | STIM2 |
| 1.36E-91 | 0.421726572 | 0.114 | 0.03 | 2.44E-87 | LZ_CD9 | ABCA1 |
| 3.30E-28 | 0.4211847 | 0.237 | 0.156 | 5.94E-24 | LZ_CD9 | BAZ2A |
| 4.06E-38 | 0.421174507 | 0.153 | 0.078 | 7.32E-34 | LZ_CD9 | METRNL |
| 1.04E-32 | 0.420209414 | 0.184 | 0.105 | 1.88E-28 | LZ_CD9 | SCRN1 |
| 2.36E-43 | 0.419926911 | 0.577 | 0.48 | 4.26E-39 | LZ_CD9 | REL |
| 3.30E-28 | 0.418952037 | 0.221 | 0.141 | 5.94E-24 | LZ_CD9 | MBP |
| 1.27E-35 | 0.416317192 | 0.242 | 0.151 | 2.28E-31 | LZ_CD9 | FAM102A |
| 4.41E-16 | 0.416261996 | 0.376 | 0.309 | 7.95E-12 | LZ_CD9 | RGS1 |
| 5.32E-33 | 0.415613943 | 0.162 | 0.088 | 9.59E-29 | LZ_CD9 | CCDC144A |
| 4.57E-43 | 0.412660983 | 0.182 | 0.093 | 8.24E-39 | LZ_CD9 | OAS1 |
| 6.43E-19 | 0.410732362 | 0.508 | 0.462 | 1.16E-14 | LZ_CD9 | SAT1 |
| 1.45E-23 | 0.410628359 | 0.24 | 0.165 | 2.62E-19 | LZ_CD9 | RABGAP1 |
| 6.02E-16 | 0.405515387 | 0.135 | 0.086 | 1.08E-11 | LZ_CD9 | IGHG2 |
| 6.76E-30 | 0.405213017 | 0.554 | 0.486 | 1.22E-25 | LZ_CD9 | SMARCB1 |
| 4.74E-25 | 0.404952251 | 0.57 | 0.499 | 8.54E-21 | LZ_CD9 | SYK |
| 6.26E-61 | 0.404330086 | 0.12 | 0.043 | 1.13E-56 | LZ_CD9 | STXBP6 |
| 2.10E-18 | 0.404305675 | 0.226 | 0.165 | 3.78E-14 | LZ_CD9 | DYNC1H1 |
| 8.67E-23 | 0.404089112 | 0.473 | 0.398 | 1.56E-18 | LZ_CD9 | GCSAM |
| 4.18E-28 | 0.403620417 | 0.227 | 0.15 | 7.53E-24 | LZ_CD9 | RAB30-AS1 |
| 5.13E-33 | 0.402775887 | 0.145 | 0.077 | 9.25E-29 | LZ_CD9 | SLC41A1 |
| 3.03E-52 | 0.402705019 | 0.743 | 0.656 | 5.47E-48 | LZ_CD9 | LSP1 |
| 3.17E-65 | 0.398361678 | 0.132 | 0.048 | 5.71E-61 | LZ_CD9 | KANK1 |
| 6.32E-33 | 0.397294598 | 0.243 | 0.152 | 1.14E-28 | LZ_CD9 | SUSD6 |
| 2.16E-15 | 0.396669769 | 0.139 | 0.094 | 3.89E-11 | LZ_CD9 | ZBED5-AS1 |
| 8.24E-31 | 0.394889139 | 0.36 | 0.258 | 1.48E-26 | LZ_CD9 | SP110 |
| 1.05E-09 | 0.394592597 | 0.227 | 0.19 | 1.89E-05 | LZ_CD9 | GABPB1-AS1 |
| 8.82E-30 | 0.393650414 | 0.157 | 0.089 | 1.59E-25 | LZ_CD9 | FUCA1 |
| 2.43E-23 | 0.393562909 | 0.473 | 0.405 | 4.38E-19 | LZ_CD9 | ZNF106 |
| 8.37E-40 | 0.392778223 | 0.227 | 0.13 | 1.51E-35 | LZ_CD9 | COPS9 |
| 3.53E-27 | 0.392188829 | 0.532 | 0.452 | 6.36E-23 | LZ_CD9 | BTG2 |
| 9.64E-22 | 0.391411756 | 0.27 | 0.192 | 1.74E-17 | LZ_CD9 | CD24 |
| 4.70E-39 | 0.390835671 | 0.243 | 0.142 | 8.48E-35 | LZ_CD9 | WASHC4 |
| 3.46E-27 | 0.39030212 | 0.25 | 0.17 | 6.24E-23 | LZ_CD9 | OSBPL10 |
| 2.85E-34 | 0.389281493 | 0.366 | 0.25 | 5.14E-30 | LZ_CD9 | TRIR |
| 4.32E-21 | 0.389264342 | 0.216 | 0.151 | 7.79E-17 | LZ_CD9 | MDM2 |
| 1.81E-29 | 0.388910569 | 0.163 | 0.092 | 3.26E-25 | LZ_CD9 | GPT2 |
| 2.32E-28 | 0.387282322 | 0.278 | 0.19 | 4.18E-24 | LZ_CD9 | FAM214A |
| 2.56E-40 | 0.386727357 | 0.775 | 0.703 | 4.61E-36 | LZ_CD9 | PTP4A2 |
| 7.03E-79 | 0.386479288 | 0.107 | 0.03 | 1.27E-74 | LZ_CD9 | BCR |
| 8.91E-43 | 0.386354596 | 0.135 | 0.062 | 1.61E-38 | LZ_CD9 | FAM241A |
| 5.28E-48 | 0.385717384 | 0.876 | 0.849 | 9.52E-44 | LZ_CD9 | BTG1 |
| 4.27E-22 | 0.385487916 | 0.437 | 0.369 | 7.69E-18 | LZ_CD9 | HSPA4 |
| 4.34E-40 | 0.384843531 | 0.146 | 0.071 | 7.82E-36 | LZ_CD9 | AC084033.3 |
| 6.26E-21 | 0.383231437 | 0.448 | 0.385 | 1.13E-16 | LZ_CD9 | DARS |
| 1.86E-52 | 0.378224301 | 0.105 | 0.038 | 3.36E-48 | LZ_CD9 | S100Z |
| 1.38E-30 | 0.375042869 | 0.662 | 0.618 | 2.49E-26 | LZ_CD9 | HERPUD1 |
| 2.68E-21 | 0.374824229 | 0.463 | 0.398 | 4.82E-17 | LZ_CD9 | ACADM |
| 1.02E-22 | 0.374608518 | 0.387 | 0.312 | 1.84E-18 | LZ_CD9 | SOCS1 |
| 3.76E-18 | 0.373873676 | 0.426 | 0.367 | 6.77E-14 | LZ_CD9 | CLIC4 |
| 1.03E-21 | 0.373549425 | 0.515 | 0.455 | 1.86E-17 | LZ_CD9 | UBE2J1 |
| 1.61E-22 | 0.372793524 | 0.316 | 0.237 | 2.90E-18 | LZ_CD9 | EDEM1 |
| 2.01E-24 | 0.36985979 | 0.541 | 0.475 | 3.63E-20 | LZ_CD9 | PAIP2 |
| 1.33E-42 | 0.369490448 | 0.782 | 0.706 | 2.40E-38 | LZ_CD9 | YWHAZ |
| 4.06E-34 | 0.368527568 | 0.508 | 0.397 | 7.32E-30 | LZ_CD9 | CPNE5 |
| 4.93E-42 | 0.367687695 | 0.293 | 0.177 | 8.89E-38 | LZ_CD9 | GNG7 |
| 4.24E-32 | 0.36393737 | 0.602 | 0.541 | 7.65E-28 | LZ_CD9 | RAP1B |
| 4.87E-16 | 0.363787697 | 0.252 | 0.194 | 8.77E-12 | LZ_CD9 | OS9 |
| 1.59E-25 | 0.363323972 | 0.306 | 0.223 | 2.87E-21 | LZ_CD9 | PRDM2 |
| 1.19E-27 | 0.362827711 | 0.23 | 0.149 | 2.15E-23 | LZ_CD9 | RICTOR |
| 2.38E-21 | 0.361855088 | 0.475 | 0.412 | 4.28E-17 | LZ_CD9 | EPS15 |
| 4.16E-21 | 0.361668596 | 0.353 | 0.275 | 7.50E-17 | LZ_CD9 | TRAF5 |
| 1.06E-26 | 0.360831118 | 0.148 | 0.084 | 1.90E-22 | LZ_CD9 | TMEM129 |
| 4.76E-72 | 0.360531752 | 0.991 | 0.984 | 8.57E-68 | LZ_CD9 | TPT1 |
| 1.37E-16 | 0.358613645 | 0.25 | 0.187 | 2.47E-12 | LZ_CD9 | DUSP1 |
| 3.15E-08 | 0.358371813 | 0.168 | 0.132 | 0.000568123 | LZ_CD9 | DHTKD1 |
| 1.89E-05 | 0.358283026 | 0.22 | 0.196 | 0.339746498 | LZ_CD9 | XBP1 |
| 1.13E-25 | 0.357697535 | 0.192 | 0.121 | 2.04E-21 | LZ_CD9 | SLCO3A1 |
| 1.68E-76 | 0.357189788 | 0.952 | 0.935 | 3.02E-72 | LZ_CD9 | EIF1 |
| 7.77E-24 | 0.356987938 | 0.384 | 0.307 | 1.40E-19 | LZ_CD9 | CCNDBP1 |
| 4.26E-17 | 0.356393197 | 0.27 | 0.211 | 7.67E-13 | LZ_CD9 | UBE2R2 |
| 3.23E-18 | 0.355902172 | 0.368 | 0.305 | 5.82E-14 | LZ_CD9 | TANK |
| 1.61E-28 | 0.355769236 | 0.466 | 0.386 | 2.90E-24 | LZ_CD9 | IGBP1 |
| 1.13E-13 | 0.355760146 | 0.299 | 0.243 | 2.04E-09 | LZ_CD9 | SLC44A2 |
| 6.41E-20 | 0.354442139 | 0.554 | 0.485 | 1.16E-15 | LZ_CD9 | LPP |
| 3.28E-30 | 0.353138761 | 0.138 | 0.074 | 5.90E-26 | LZ_CD9 | THAP3 |
| 3.96E-48 | 0.353000748 | 0.123 | 0.05 | 7.13E-44 | LZ_CD9 | AL133467.1 |
| 1.53E-16 | 0.350437473 | 0.202 | 0.144 | 2.76E-12 | LZ_CD9 | RNF41 |
| 1.64E-30 | 0.349871934 | 0.556 | 0.46 | 2.96E-26 | LZ_CD9 | EVI2B |
| 5.94E-11 | 0.349449668 | 0.363 | 0.324 | 1.07E-06 | LZ_CD9 | RPIA |
| 7.78E-18 | 0.349293353 | 0.282 | 0.217 | 1.40E-13 | LZ_CD9 | FAM208B |
| 1.19E-33 | 0.349269137 | 0.195 | 0.112 | 2.15E-29 | LZ_CD9 | ERBIN |
| 3.24E-27 | 0.348962052 | 0.699 | 0.657 | 5.84E-23 | LZ_CD9 | GNAS |
| 1.23E-56 | 0.348805171 | 0.123 | 0.046 | 2.21E-52 | LZ_CD9 | LINC01991 |
| 2.14E-20 | 0.348465626 | 0.465 | 0.384 | 3.86E-16 | LZ_CD9 | PTK2B |
| 9.24E-82 | 0.347099521 | 0.115 | 0.033 | 1.67E-77 | LZ_CD9 | AC058791.1 |
| 6.86E-19 | 0.346171498 | 0.147 | 0.093 | 1.24E-14 | LZ_CD9 | GFOD1 |
| 2.32E-21 | 0.344976061 | 0.219 | 0.152 | 4.18E-17 | LZ_CD9 | ARL2BP |
| 4.90E-24 | 0.344795462 | 0.62 | 0.579 | 8.83E-20 | LZ_CD9 | SEPT6 |
| 2.09E-21 | 0.344077258 | 0.207 | 0.141 | 3.76E-17 | LZ_CD9 | BFSP2 |
| 4.56E-13 | 0.343658607 | 0.268 | 0.214 | 8.22E-09 | LZ_CD9 | R3HDM4 |
| 3.78E-87 | 0.343570925 | 0.961 | 0.922 | 6.82E-83 | LZ_CD9 | LAPTM5 |
| 4.25E-20 | 0.341869608 | 0.305 | 0.233 | 7.66E-16 | LZ_CD9 | CAMK2D |
| 1.63E-15 | 0.340433129 | 0.415 | 0.356 | 2.94E-11 | LZ_CD9 | TERF2 |
| 3.39E-18 | 0.338838518 | 0.276 | 0.212 | 6.11E-14 | LZ_CD9 | KLHL5 |
| 1.33E-08 | 0.338365325 | 0.25 | 0.214 | 0.000238884 | LZ_CD9 | KIAA0040 |
| 1.21E-29 | 0.337232782 | 0.517 | 0.434 | 2.18E-25 | LZ_CD9 | RSRP1 |
| 4.67E-45 | 0.336676936 | 0.775 | 0.707 | 8.41E-41 | LZ_CD9 | EIF3H |
| 1.37E-12 | 0.33658302 | 0.216 | 0.169 | 2.46E-08 | LZ_CD9 | LINC00467 |
| 1.94E-23 | 0.335682789 | 0.579 | 0.515 | 3.49E-19 | LZ_CD9 | TOP1 |
| 1.02E-18 | 0.334855073 | 0.56 | 0.51 | 1.83E-14 | LZ_CD9 | CSDE1 |
| 7.85E-17 | 0.334374195 | 0.432 | 0.382 | 1.42E-12 | LZ_CD9 | DYNLRB1 |
| 3.06E-15 | 0.334211179 | 0.554 | 0.514 | 5.51E-11 | LZ_CD9 | UCP2 |
| 2.55E-16 | 0.333996416 | 0.155 | 0.103 | 4.60E-12 | LZ_CD9 | RNF19B |
| 2.88E-37 | 0.333714443 | 0.702 | 0.651 | 5.20E-33 | LZ_CD9 | SEC62 |
| 2.05E-11 | 0.333435094 | 0.219 | 0.173 | 3.69E-07 | LZ_CD9 | ILF3-AS1 |
| 1.17E-21 | 0.333426569 | 0.683 | 0.631 | 2.10E-17 | LZ_CD9 | EIF4G2 |
| 6.59E-22 | 0.332999259 | 0.57 | 0.515 | 1.19E-17 | LZ_CD9 | NCF1 |
| 3.61E-19 | 0.332211688 | 0.511 | 0.446 | 6.51E-15 | LZ_CD9 | POLD4 |
| 1.57E-17 | 0.332090791 | 0.431 | 0.369 | 2.84E-13 | LZ_CD9 | SP140 |
| 2.01E-16 | 0.331361092 | 0.251 | 0.191 | 3.63E-12 | LZ_CD9 | ACSF3 |
| 3.09E-68 | 0.330910589 | 0.123 | 0.042 | 5.57E-64 | LZ_CD9 | ATP2B1-AS1 |
| 6.87E-35 | 0.330454115 | 0.196 | 0.111 | 1.24E-30 | LZ_CD9 | CREBRF |
| 5.27E-29 | 0.330142535 | 0.621 | 0.55 | 9.50E-25 | LZ_CD9 | DDX17 |
| 3.88E-28 | 0.329700297 | 0.341 | 0.236 | 6.99E-24 | LZ_CD9 | RIPOR2 |
| 3.29E-25 | 0.327186937 | 0.184 | 0.113 | 5.93E-21 | LZ_CD9 | LINC01215 |
| 2.04E-13 | 0.32694514 | 0.215 | 0.164 | 3.67E-09 | LZ_CD9 | PNPLA8 |
| 1.05E-20 | 0.326688325 | 0.536 | 0.475 | 1.89E-16 | LZ_CD9 | LYN |
| 4.01E-20 | 0.326432914 | 0.283 | 0.209 | 7.23E-16 | LZ_CD9 | FOXO1 |
| 3.24E-15 | 0.326280853 | 0.417 | 0.359 | 5.84E-11 | LZ_CD9 | BRD4 |
| 2.03E-13 | 0.325263443 | 0.249 | 0.199 | 3.65E-09 | LZ_CD9 | MICU2 |
| 7.90E-30 | 0.324782176 | 0.299 | 0.202 | 1.42E-25 | LZ_CD9 | FCRL2 |
| 1.21E-24 | 0.323336346 | 0.554 | 0.489 | 2.18E-20 | LZ_CD9 | MDM4 |
| 1.85E-20 | 0.32309859 | 0.156 | 0.098 | 3.34E-16 | LZ_CD9 | IL10RA |
| 9.18E-24 | 0.322866417 | 0.567 | 0.51 | 1.65E-19 | LZ_CD9 | POLR1D |
| 6.05E-14 | 0.321818094 | 0.203 | 0.151 | 1.09E-09 | LZ_CD9 | DENND4A |
| 1.55E-21 | 0.321705555 | 0.513 | 0.446 | 2.80E-17 | LZ_CD9 | VAMP2 |
| 1.53E-07 | 0.320706835 | 0.219 | 0.187 | 0.002751512 | LZ_CD9 | PCGF5 |
| 1.37E-32 | 0.3202862 | 0.714 | 0.669 | 2.46E-28 | LZ_CD9 | SMDT1 |
| 2.83E-24 | 0.319950344 | 0.544 | 0.479 | 5.10E-20 | LZ_CD9 | ITSN2 |
| 1.26E-27 | 0.31825149 | 0.706 | 0.636 | 2.27E-23 | LZ_CD9 | MTDH |
| 1.67E-18 | 0.318038854 | 0.126 | 0.077 | 3.00E-14 | LZ_CD9 | CPTP |
| 8.20E-16 | 0.317927754 | 0.427 | 0.374 | 1.48E-11 | LZ_CD9 | ATP2B1 |
| 1.26E-29 | 0.317751698 | 0.185 | 0.108 | 2.28E-25 | LZ_CD9 | ZNF141 |
| 1.05E-27 | 0.316238057 | 0.737 | 0.678 | 1.89E-23 | LZ_CD9 | METAP2 |
| 2.48E-11 | 0.315970151 | 0.249 | 0.203 | 4.46E-07 | LZ_CD9 | KIF2A |
| 1.11E-29 | 0.315889638 | 0.178 | 0.103 | 2.00E-25 | LZ_CD9 | ITPR1 |
| 1.14E-31 | 0.314954212 | 0.138 | 0.07 | 2.06E-27 | LZ_CD9 | AL512631.1 |
| 4.27E-14 | 0.314698413 | 0.456 | 0.41 | 7.69E-10 | LZ_CD9 | CYB5R3 |
| 1.96E-12 | 0.313275939 | 0.261 | 0.211 | 3.52E-08 | LZ_CD9 | ZNF148 |
| 4.68E-29 | 0.312835873 | 0.116 | 0.058 | 8.43E-25 | LZ_CD9 | AC012236.1 |
| 4.61E-09 | 0.312775028 | 0.361 | 0.333 | 8.31E-05 | LZ_CD9 | SYAP1 |
| 2.01E-13 | 0.311660968 | 0.264 | 0.211 | 3.62E-09 | LZ_CD9 | MTA3 |
| 1.27E-24 | 0.311410195 | 0.57 | 0.499 | 2.30E-20 | LZ_CD9 | CCDC69 |
| 3.53E-05 | 0.31138573 | 0.345 | 0.333 | 0.636174523 | LZ_CD9 | PMAIP1 |
| 1.72E-14 | 0.310901088 | 0.343 | 0.284 | 3.11E-10 | LZ_CD9 | EHD1 |
| 7.40E-13 | 0.30980627 | 0.453 | 0.411 | 1.33E-08 | LZ_CD9 | CXXC5 |
| 5.32E-22 | 0.309417425 | 0.163 | 0.101 | 9.58E-18 | LZ_CD9 | ZC3H6 |
| 2.53E-10 | 0.309309527 | 0.354 | 0.316 | 4.55E-06 | LZ_CD9 | NSRP1 |
| 2.20E-20 | 0.309114883 | 0.593 | 0.521 | 3.96E-16 | LZ_CD9 | RBBP7 |
| 2.51E-13 | 0.308792513 | 0.357 | 0.303 | 4.53E-09 | LZ_CD9 | RAB30 |
| 5.78E-14 | 0.307362062 | 0.348 | 0.292 | 1.04E-09 | LZ_CD9 | KDM5A |
| 4.94E-11 | 0.305661953 | 0.237 | 0.191 | 8.90E-07 | LZ_CD9 | REPIN1 |
| 1.52E-25 | 0.303528014 | 0.341 | 0.241 | 2.73E-21 | LZ_CD9 | EBLN3P |
| 8.68E-16 | 0.303427858 | 0.304 | 0.243 | 1.57E-11 | LZ_CD9 | ITGB1 |
| 4.29E-23 | 0.303284164 | 0.246 | 0.169 | 7.74E-19 | LZ_CD9 | SYNE1 |
| 2.47E-14 | 0.30242043 | 0.496 | 0.441 | 4.46E-10 | LZ_CD9 | IKZF3 |
| 5.13E-15 | 0.302179076 | 0.192 | 0.139 | 9.25E-11 | LZ_CD9 | RNF13 |
| 3.03E-21 | 0.301917648 | 0.386 | 0.312 | 5.46E-17 | LZ_CD9 | ZNF581 |
| 0.000105433 | 0.301711544 | 0.166 | 0.146 | 1 | LZ_CD9 | REXO2 |
| 1.05E-10 | 0.301462355 | 0.213 | 0.17 | 1.89E-06 | LZ_CD9 | USP7 |
| 3.72E-22 | 0.300115504 | 0.646 | 0.603 | 6.70E-18 | LZ_CD9 | CD81 |
| 5.03E-16 | 0.299271343 | 0.192 | 0.136 | 9.06E-12 | LZ_CD9 | LRCH1 |
| 1.44E-23 | 0.299094839 | 0.192 | 0.124 | 2.59E-19 | LZ_CD9 | GRAP |
| 4.82E-10 | 0.299091416 | 0.298 | 0.255 | 8.68E-06 | LZ_CD9 | PIAS1 |
| 4.08E-14 | 0.298370348 | 0.318 | 0.259 | 7.35E-10 | LZ_CD9 | SYNRG |
| 3.09E-11 | 0.298147926 | 0.236 | 0.188 | 5.57E-07 | LZ_CD9 | RAB3GAP1 |
| 2.29E-20 | 0.297327764 | 0.198 | 0.131 | 4.13E-16 | LZ_CD9 | RAPGEF5 |
| 3.96E-18 | 0.296574738 | 0.517 | 0.467 | 7.14E-14 | LZ_CD9 | TMEM243 |
| 1.24E-15 | 0.296208126 | 0.272 | 0.211 | 2.23E-11 | LZ_CD9 | SCPEP1 |
| 5.28E-17 | 0.295751787 | 0.427 | 0.366 | 9.52E-13 | LZ_CD9 | AKAP13 |
| 1.69E-06 | 0.295128494 | 0.267 | 0.237 | 0.030494927 | LZ_CD9 | ANKRD11 |
| 3.51E-13 | 0.294998779 | 0.174 | 0.126 | 6.33E-09 | LZ_CD9 | MIR3681HG |
| 1.10E-08 | 0.294927472 | 0.134 | 0.1 | 0.000197826 | LZ_CD9 | HES6 |
| 1.33E-20 | 0.2943774 | 0.17 | 0.108 | 2.39E-16 | LZ_CD9 | SLC9A7 |
| 1.97E-12 | 0.293602116 | 0.287 | 0.235 | 3.55E-08 | LZ_CD9 | ARHGAP25 |
| 2.46E-18 | 0.291314341 | 0.475 | 0.408 | 4.43E-14 | LZ_CD9 | CTSS |
| 5.60E-13 | 0.290951728 | 0.188 | 0.137 | 1.01E-08 | LZ_CD9 | STAT2 |
| 3.10E-13 | 0.290681892 | 0.206 | 0.153 | 5.59E-09 | LZ_CD9 | SLAMF6 |
| 8.69E-15 | 0.29006725 | 0.291 | 0.234 | 1.57E-10 | LZ_CD9 | PPP1R2 |
| 2.85E-11 | 0.289209134 | 0.421 | 0.379 | 5.13E-07 | LZ_CD9 | WASF2 |
| 3.70E-17 | 0.289197686 | 0.495 | 0.438 | 6.66E-13 | LZ_CD9 | TAF7 |
| 5.97E-17 | 0.288724754 | 0.173 | 0.117 | 1.08E-12 | LZ_CD9 | CD55 |
| 2.26E-15 | 0.288511306 | 0.219 | 0.161 | 4.08E-11 | LZ_CD9 | TPST2 |
| 3.24E-19 | 0.288425244 | 0.61 | 0.559 | 5.83E-15 | LZ_CD9 | CLEC2D |
| 1.10E-32 | 0.288214241 | 0.135 | 0.069 | 1.99E-28 | LZ_CD9 | MBNL2 |
| 2.40E-25 | 0.288185731 | 0.203 | 0.126 | 4.32E-21 | LZ_CD9 | MARF1 |
| 2.95E-39 | 0.287893054 | 0.122 | 0.055 | 5.31E-35 | LZ_CD9 | ABR |
| 1.81E-14 | 0.286953344 | 0.577 | 0.531 | 3.26E-10 | LZ_CD9 | PPP2CA |
| 9.74E-22 | 0.286806308 | 0.702 | 0.641 | 1.76E-17 | LZ_CD9 | TBCA |
| 1.26E-05 | 0.285885854 | 0.167 | 0.139 | 0.226914432 | LZ_CD9 | CERK |
| 4.68E-39 | 0.285601659 | 0.15 | 0.073 | 8.43E-35 | LZ_CD9 | NBDY |
| 7.52E-10 | 0.28551981 | 0.36 | 0.317 | 1.36E-05 | LZ_CD9 | VOPP1 |
| 9.47E-13 | 0.284388502 | 0.452 | 0.412 | 1.71E-08 | LZ_CD9 | KMT2E |
| 6.07E-14 | 0.284089434 | 0.201 | 0.15 | 1.09E-09 | LZ_CD9 | PPP3CC |
| 1.05E-10 | 0.283789527 | 0.201 | 0.156 | 1.89E-06 | LZ_CD9 | GOLGB1 |
| 2.95E-11 | 0.283757568 | 0.256 | 0.207 | 5.32E-07 | LZ_CD9 | CNOT6L |
| 1.15E-44 | 0.283578304 | 0.924 | 0.902 | 2.07E-40 | LZ_CD9 | ARPC3 |
| 8.46E-13 | 0.282898259 | 0.241 | 0.187 | 1.52E-08 | LZ_CD9 | ZCCHC6 |
| 2.99E-15 | 0.282421958 | 0.228 | 0.169 | 5.39E-11 | LZ_CD9 | SETDB2 |
| 2.46E-17 | 0.282304367 | 0.538 | 0.508 | 4.43E-13 | LZ_CD9 | EIF4B |
| 2.96E-13 | 0.28229119 | 0.226 | 0.172 | 5.34E-09 | LZ_CD9 | FMNL3 |
| 3.91E-26 | 0.281942385 | 0.189 | 0.115 | 7.05E-22 | LZ_CD9 | RUFY3 |
| 1.05E-27 | 0.280828626 | 0.178 | 0.105 | 1.88E-23 | LZ_CD9 | SMIM26 |
| 2.32E-19 | 0.280492577 | 0.313 | 0.226 | 4.18E-15 | LZ_CD9 | GRK2 |
| 2.44E-08 | 0.280370352 | 0.291 | 0.256 | 0.000439192 | LZ_CD9 | MAP4K2 |
| 1.05E-11 | 0.2800329 | 0.342 | 0.292 | 1.88E-07 | LZ_CD9 | UBE2G1 |
| 1.77E-18 | 0.28002101 | 0.585 | 0.543 | 3.20E-14 | LZ_CD9 | SYF2 |
| 1.45E-16 | 0.279536996 | 0.196 | 0.138 | 2.62E-12 | LZ_CD9 | ZNF280D |
| 1.65E-09 | 0.279381681 | 0.264 | 0.222 | 2.97E-05 | LZ_CD9 | TMEM248 |
| 6.70E-18 | 0.278678122 | 0.227 | 0.162 | 1.21E-13 | LZ_CD9 | ADAM17 |
| 5.68E-11 | 0.278626985 | 0.358 | 0.319 | 1.02E-06 | LZ_CD9 | CCM2 |
| 1.06E-09 | 0.278346513 | 0.315 | 0.275 | 1.90E-05 | LZ_CD9 | UBE2B |
| 8.79E-11 | 0.278183391 | 0.352 | 0.305 | 1.58E-06 | LZ_CD9 | MYO1E |
| 3.15E-21 | 0.277422427 | 0.238 | 0.162 | 5.68E-17 | LZ_CD9 | NSD3 |
| 1.31E-13 | 0.277103017 | 0.225 | 0.171 | 2.36E-09 | LZ_CD9 | TOR1AIP2 |
| 2.14E-13 | 0.275965251 | 0.145 | 0.1 | 3.86E-09 | LZ_CD9 | CTPS2 |
| 9.90E-17 | 0.275860575 | 0.651 | 0.594 | 1.78E-12 | LZ_CD9 | LSM7 |
| 6.32E-14 | 0.275600588 | 0.204 | 0.152 | 1.14E-09 | LZ_CD9 | USP8 |
| 7.12E-11 | 0.275511698 | 0.196 | 0.15 | 1.28E-06 | LZ_CD9 | CREBBP |
| 2.17E-19 | 0.275436036 | 0.154 | 0.097 | 3.91E-15 | LZ_CD9 | SELENOK |
| 5.38E-08 | 0.275113412 | 0.393 | 0.362 | 0.000970388 | LZ_CD9 | WAC |
| 3.29E-07 | 0.275022064 | 0.261 | 0.227 | 0.005928288 | LZ_CD9 | IRF1 |
| 7.70E-12 | 0.275012875 | 0.354 | 0.299 | 1.39E-07 | LZ_CD9 | MYBL1 |
| 2.55E-11 | 0.274820064 | 0.235 | 0.184 | 4.59E-07 | LZ_CD9 | EML6 |
| 3.12E-12 | 0.273609996 | 0.316 | 0.266 | 5.62E-08 | LZ_CD9 | GCC2 |
| 4.12E-14 | 0.27355484 | 0.17 | 0.119 | 7.42E-10 | LZ_CD9 | SMAD5 |
| 8.25E-29 | 0.273261735 | 0.156 | 0.086 | 1.49E-24 | LZ_CD9 | ARHGAP45 |
| 5.21E-16 | 0.273110151 | 0.612 | 0.567 | 9.40E-12 | LZ_CD9 | TKT |
| 7.02E-16 | 0.272443652 | 0.429 | 0.376 | 1.27E-11 | LZ_CD9 | PGLS |
| 1.80E-12 | 0.272346131 | 0.254 | 0.202 | 3.24E-08 | LZ_CD9 | HELZ |
| 1.63E-15 | 0.27223793 | 0.499 | 0.438 | 2.94E-11 | LZ_CD9 | CHURC1 |
| 1.39E-10 | 0.272145565 | 0.188 | 0.144 | 2.50E-06 | LZ_CD9 | RB1CC1 |
| 2.36E-16 | 0.272112159 | 0.575 | 0.526 | 4.25E-12 | LZ_CD9 | THRAP3 |
| 2.94E-14 | 0.271989133 | 0.192 | 0.136 | 5.30E-10 | LZ_CD9 | SEPHS2 |
| 5.44E-11 | 0.27189774 | 0.162 | 0.119 | 9.81E-07 | LZ_CD9 | SLC38A9 |
| 1.37E-21 | 0.271849875 | 0.19 | 0.123 | 2.47E-17 | LZ_CD9 | RASGEF1B |
| 2.55E-09 | 0.271745907 | 0.369 | 0.332 | 4.60E-05 | LZ_CD9 | CLINT1 |
| 3.34E-09 | 0.271595879 | 0.207 | 0.164 | 6.02E-05 | LZ_CD9 | ITPKB |
| 5.73E-14 | 0.271355514 | 0.258 | 0.199 | 1.03E-09 | LZ_CD9 | ANKRD44 |
| 5.82E-19 | 0.271319592 | 0.692 | 0.637 | 1.05E-14 | LZ_CD9 | PPP1CC |
| 1.19E-10 | 0.270540639 | 0.12 | 0.083 | 2.15E-06 | LZ_CD9 | TRPV2 |
| 1.12E-12 | 0.269587068 | 0.58 | 0.55 | 2.02E-08 | LZ_CD9 | SF1 |
| 1.79E-22 | 0.269402205 | 0.208 | 0.134 | 3.23E-18 | LZ_CD9 | ELOC |
| 5.65E-12 | 0.269282444 | 0.517 | 0.481 | 1.02E-07 | LZ_CD9 | CSTB |
| 5.01E-07 | 0.269178983 | 0.168 | 0.137 | 0.009030469 | LZ_CD9 | CCDC117 |
| 7.47E-20 | 0.268822188 | 0.183 | 0.12 | 1.35E-15 | LZ_CD9 | STMP1 |
| 1.36E-13 | 0.268661047 | 0.153 | 0.106 | 2.45E-09 | LZ_CD9 | CAMP |
| 1.45E-10 | 0.26852412 | 0.193 | 0.148 | 2.61E-06 | LZ_CD9 | MED13 |
| 1.77E-11 | 0.268273996 | 0.255 | 0.206 | 3.18E-07 | LZ_CD9 | RIOK3 |
| 1.04E-11 | 0.267672982 | 0.524 | 0.501 | 1.88E-07 | LZ_CD9 | PNN |
| 2.80E-14 | 0.266634469 | 0.197 | 0.142 | 5.05E-10 | LZ_CD9 | AFF4 |
| 4.02E-12 | 0.265291863 | 0.359 | 0.312 | 7.24E-08 | LZ_CD9 | IAH1 |
| 4.45E-08 | 0.265101624 | 0.419 | 0.387 | 0.000801679 | LZ_CD9 | CELF1 |
| 3.52E-15 | 0.263754447 | 0.113 | 0.07 | 6.34E-11 | LZ_CD9 | NCK2 |
| 4.05E-13 | 0.263247534 | 0.204 | 0.152 | 7.31E-09 | LZ_CD9 | CSNK2A2 |
| 1.83E-18 | 0.262859263 | 0.188 | 0.126 | 3.29E-14 | LZ_CD9 | AFF2 |
| 2.08E-21 | 0.262265297 | 0.693 | 0.648 | 3.75E-17 | LZ_CD9 | EIF3F |
| 2.19E-15 | 0.261941988 | 0.169 | 0.115 | 3.95E-11 | LZ_CD9 | KMT5B |
| 4.74E-11 | 0.261928352 | 0.416 | 0.375 | 8.54E-07 | LZ_CD9 | LYPLAL1 |
| 1.02E-07 | 0.261701775 | 0.194 | 0.158 | 0.001832958 | LZ_CD9 | ATXN7L3B |
| 5.71E-23 | 0.260747215 | 0.733 | 0.705 | 1.03E-18 | LZ_CD9 | COMMD6 |
| 2.46E-12 | 0.260610489 | 0.136 | 0.092 | 4.44E-08 | LZ_CD9 | FAM30A |
| 1.39E-12 | 0.260512855 | 0.129 | 0.087 | 2.50E-08 | LZ_CD9 | TSPAN14 |
| 2.28E-13 | 0.260218557 | 0.109 | 0.069 | 4.12E-09 | LZ_CD9 | MAP4K5 |
| 8.96E-11 | 0.25990236 | 0.246 | 0.196 | 1.62E-06 | LZ_CD9 | KIAA1551 |
| 0.000939208 | 0.259101245 | 0.176 | 0.157 | 1 | LZ_CD9 | MEN1 |
| 2.48E-11 | 0.259089582 | 0.334 | 0.285 | 4.47E-07 | LZ_CD9 | EPC1 |
| 6.03E-14 | 0.258998526 | 0.531 | 0.487 | 1.09E-09 | LZ_CD9 | CDV3 |
| 4.76E-13 | 0.258960233 | 0.521 | 0.473 | 8.58E-09 | LZ_CD9 | ATF4 |
| 1.90E-12 | 0.258641727 | 0.252 | 0.2 | 3.42E-08 | LZ_CD9 | DCUN1D1 |
| 9.72E-16 | 0.258488707 | 0.611 | 0.567 | 1.75E-11 | LZ_CD9 | RSL24D1 |
| 3.33E-13 | 0.258363844 | 0.132 | 0.09 | 6.00E-09 | LZ_CD9 | C6orf226 |
| 2.17E-23 | 0.258289003 | 0.28 | 0.196 | 3.90E-19 | LZ_CD9 | GLIPR1 |
| 5.43E-30 | 0.258032894 | 0.702 | 0.638 | 9.78E-26 | LZ_CD9 | TMEM123 |
| 2.31E-22 | 0.257638694 | 0.771 | 0.722 | 4.15E-18 | LZ_CD9 | ACTR2 |
| 1.08E-10 | 0.257543079 | 0.294 | 0.24 | 1.94E-06 | LZ_CD9 | LBH |
| 1.42E-45 | 0.257202327 | 0.962 | 0.932 | 2.55E-41 | LZ_CD9 | ARHGDIB |
| 2.11E-13 | 0.257053618 | 0.188 | 0.137 | 3.81E-09 | LZ_CD9 | HIP1R |
| 3.73E-11 | 0.256770171 | 0.492 | 0.452 | 6.72E-07 | LZ_CD9 | ITGAE |
| 5.64E-09 | 0.256637874 | 0.526 | 0.514 | 0.000101627 | LZ_CD9 | ELF1 |
| 2.56E-15 | 0.256573036 | 0.405 | 0.34 | 4.61E-11 | LZ_CD9 | BCL6 |
| 3.92E-09 | 0.256527467 | 0.192 | 0.151 | 7.07E-05 | LZ_CD9 | KAT6A |
| 5.22E-25 | 0.256415506 | 0.126 | 0.068 | 9.41E-21 | LZ_CD9 | ARRDC3 |
| 1.16E-13 | 0.256354041 | 0.214 | 0.16 | 2.10E-09 | LZ_CD9 | ACYP2 |
| 5.46E-09 | 0.256089974 | 0.343 | 0.309 | 9.84E-05 | LZ_CD9 | MPLKIP |
| 1.06E-14 | 0.255775938 | 0.194 | 0.138 | 1.92E-10 | LZ_CD9 | KLHL24 |
| 4.03E-10 | 0.255494239 | 0.102 | 0.068 | 7.26E-06 | LZ_CD9 | RETREG3 |
| 1.20E-08 | 0.255356709 | 0.221 | 0.182 | 0.000216241 | LZ_CD9 | FANCA |
| 4.20E-13 | 0.254800617 | 0.484 | 0.439 | 7.57E-09 | LZ_CD9 | SPI1 |
| 4.12E-06 | 0.254763163 | 0.183 | 0.154 | 0.074240925 | LZ_CD9 | TRAPPC2 |
| 2.84E-23 | 0.254693394 | 0.144 | 0.085 | 5.12E-19 | LZ_CD9 | UGGT1 |
| 2.42E-10 | 0.254468143 | 0.161 | 0.119 | 4.37E-06 | LZ_CD9 | NF1 |
| 3.93E-08 | 0.254324981 | 0.29 | 0.257 | 0.000708788 | LZ_CD9 | TAF10 |
| 7.66E-16 | 0.25400973 | 0.488 | 0.432 | 1.38E-11 | LZ_CD9 | SNAP23 |
| 0.001583399 | 0.253880621 | 0.268 | 0.253 | 1 | LZ_CD9 | RBBP6 |
| 5.11E-11 | 0.252477002 | 0.236 | 0.19 | 9.20E-07 | LZ_CD9 | NSMCE3 |
| 4.17E-30 | 0.251843645 | 0.78 | 0.709 | 7.52E-26 | LZ_CD9 | SMIM14 |
| 1.13E-17 | 0.251690633 | 0.124 | 0.075 | 2.04E-13 | LZ_CD9 | SYT17 |
| 3.20E-18 | 0.250267901 | 0.228 | 0.16 | 5.77E-14 | LZ_CD9 | RHBDF2 |
| 3.18E-18 | -0.250430005 | 0.517 | 0.588 | 5.73E-14 | LZ_CD9 | BANF1 |
| 1.19E-12 | -0.250663956 | 0.299 | 0.376 | 2.14E-08 | LZ_CD9 | C9orf16 |
| 1.93E-41 | -0.250781619 | 0.052 | 0.161 | 3.48E-37 | LZ_CD9 | LMNB2 |
| 5.34E-05 | -0.251004407 | 0.484 | 0.501 | 0.962258991 | LZ_CD9 | C1QBP |
| 2.12E-32 | -0.251800176 | 0.079 | 0.179 | 3.83E-28 | LZ_CD9 | NIT2 |
| 1.16E-30 | -0.252464999 | 0.094 | 0.195 | 2.09E-26 | LZ_CD9 | MRPL17 |
| 6.35E-24 | -0.253386206 | 0.24 | 0.355 | 1.14E-19 | LZ_CD9 | PMVK |
| 1.70E-26 | -0.253696534 | 0.129 | 0.231 | 3.07E-22 | LZ_CD9 | TXNDC12 |
| 1.16E-20 | -0.253944934 | 0.157 | 0.253 | 2.09E-16 | LZ_CD9 | AC090498.1 |
| 1.30E-11 | -0.254695027 | 0.514 | 0.57 | 2.35E-07 | LZ_CD9 | DCK |
| 3.02E-18 | -0.255059572 | 0.372 | 0.468 | 5.44E-14 | LZ_CD9 | RALY |
| 4.08E-32 | -0.255206743 | 0.071 | 0.17 | 7.36E-28 | LZ_CD9 | TROAP |
| 3.03E-17 | -0.255375231 | 0.101 | 0.17 | 5.47E-13 | LZ_CD9 | BCAR3 |
| 2.16E-41 | -0.255471921 | 0.015 | 0.101 | 3.90E-37 | LZ_CD9 | KIF18A |
| 4.34E-39 | -0.255731811 | 0.028 | 0.119 | 7.82E-35 | LZ_CD9 | CEP78 |
| 3.50E-47 | -0.256790519 | 0.045 | 0.162 | 6.30E-43 | LZ_CD9 | ZWILCH |
| 3.24E-18 | -0.256906179 | 0.324 | 0.426 | 5.84E-14 | LZ_CD9 | FERMT3 |
| 1.99E-18 | -0.257401663 | 0.277 | 0.372 | 3.58E-14 | LZ_CD9 | RNASEH2C |
| 6.71E-28 | -0.257486271 | 0.138 | 0.246 | 1.21E-23 | LZ_CD9 | RHEB |
| 1.26E-13 | -0.257672281 | 0.085 | 0.14 | 2.28E-09 | LZ_CD9 | NSD2 |
| 8.47E-23 | -0.258183013 | 0.291 | 0.405 | 1.53E-18 | LZ_CD9 | PTBP1 |
| 2.54E-27 | -0.258461643 | 0.066 | 0.152 | 4.58E-23 | LZ_CD9 | S100A6 |
| 2.11E-08 | -0.258473641 | 0.327 | 0.394 | 0.000381012 | LZ_CD9 | NFKBIA |
| 1.94E-48 | -0.258527619 | 0.049 | 0.17 | 3.50E-44 | LZ_CD9 | ATAD5 |
| 7.56E-52 | -0.260402309 | 0.013 | 0.115 | 1.36E-47 | LZ_CD9 | TRIP13 |
| 5.89E-08 | -0.261954413 | 0.698 | 0.76 | 0.001060979 | LZ_CD9 | CD22 |
| 1.81E-20 | -0.262434599 | 0.192 | 0.289 | 3.26E-16 | LZ_CD9 | FKBP2 |
| 7.47E-33 | -0.26247122 | 0.159 | 0.284 | 1.35E-28 | LZ_CD9 | WDR76 |
| 6.45E-18 | -0.263073391 | 0.496 | 0.569 | 1.16E-13 | LZ_CD9 | XRCC5 |
| 1.61E-19 | -0.263278188 | 0.372 | 0.486 | 2.90E-15 | LZ_CD9 | MTF2 |
| 1.19E-18 | -0.263387016 | 0.259 | 0.358 | 2.14E-14 | LZ_CD9 | MRPL57 |
| 3.71E-22 | -0.263534874 | 0.846 | 0.843 | 6.69E-18 | LZ_CD9 | YBX1 |
| 3.73E-38 | -0.263555515 | 0.076 | 0.188 | 6.72E-34 | LZ_CD9 | FH |
| 2.76E-19 | -0.263742409 | 0.522 | 0.603 | 4.97E-15 | LZ_CD9 | HNRNPDL |
| 6.60E-26 | -0.263760956 | 0.207 | 0.32 | 1.19E-21 | LZ_CD9 | SNRNP40 |
| 1.41E-55 | -0.264098063 | 0.02 | 0.134 | 2.54E-51 | LZ_CD9 | GGH |
| 2.03E-18 | -0.265091603 | 0.612 | 0.652 | 3.66E-14 | LZ_CD9 | COX5A |
| 1.77E-24 | -0.265436151 | 0.142 | 0.242 | 3.19E-20 | LZ_CD9 | ATIC |
| 6.17E-25 | -0.266278811 | 0.15 | 0.251 | 1.11E-20 | LZ_CD9 | MRPS26 |
| 1.49E-32 | -0.266335021 | 0.065 | 0.16 | 2.68E-28 | LZ_CD9 | ZNF672 |
| 2.07E-25 | -0.266737672 | 0.24 | 0.356 | 3.72E-21 | LZ_CD9 | MTHFD2 |
| 8.99E-10 | -0.267392545 | 0.318 | 0.372 | 1.62E-05 | LZ_CD9 | NHP2 |
| 7.59E-37 | -0.267597976 | 0.051 | 0.15 | 1.37E-32 | LZ_CD9 | RANGAP1 |
| 3.49E-13 | -0.268048891 | 0.403 | 0.468 | 6.29E-09 | LZ_CD9 | PPA1 |
| 2.94E-25 | -0.269852164 | 0.22 | 0.335 | 5.30E-21 | LZ_CD9 | SUZ12 |
| 9.33E-15 | -0.269861569 | 0.34 | 0.422 | 1.68E-10 | LZ_CD9 | CYCS |
| 5.61E-14 | -0.270957207 | 0.468 | 0.56 | 1.01E-09 | LZ_CD9 | CTSH |
| 1.16E-22 | -0.271376557 | 0.192 | 0.295 | 2.09E-18 | LZ_CD9 | PDLIM1 |
| 5.78E-47 | -0.272175893 | 0.054 | 0.174 | 1.04E-42 | LZ_CD9 | DSN1 |
| 2.44E-29 | -0.272228648 | 0.251 | 0.382 | 4.39E-25 | LZ_CD9 | AK2 |
| 1.61E-15 | -0.273288596 | 0.103 | 0.168 | 2.90E-11 | LZ_CD9 | RASGRP1 |
| 9.03E-52 | -0.274847872 | 0.007 | 0.105 | 1.63E-47 | LZ_CD9 | MCM10 |
| 2.83E-33 | -0.275468032 | 0.05 | 0.141 | 5.10E-29 | LZ_CD9 | APOLD1 |
| 9.28E-20 | -0.276173044 | 0.169 | 0.257 | 1.67E-15 | LZ_CD9 | TOMM5 |
| 1.52E-27 | -0.276259795 | 0.201 | 0.316 | 2.73E-23 | LZ_CD9 | C19orf48 |
| 8.86E-39 | -0.276298315 | 0.065 | 0.172 | 1.60E-34 | LZ_CD9 | SAC3D1 |
| 7.84E-36 | -0.27663366 | 0.053 | 0.149 | 1.41E-31 | LZ_CD9 | TRAP1 |
| 8.82E-25 | -0.277169839 | 0.066 | 0.145 | 1.59E-20 | LZ_CD9 | S100A4 |
| 2.65E-17 | -0.277348837 | 0.309 | 0.409 | 4.77E-13 | LZ_CD9 | HVCN1 |
| 5.58E-22 | -0.278182491 | 0.448 | 0.531 | 1.01E-17 | LZ_CD9 | RPA3 |
| 1.45E-17 | -0.278397472 | 0.358 | 0.446 | 2.62E-13 | LZ_CD9 | CARHSP1 |
| 1.11E-20 | -0.278605298 | 0.229 | 0.325 | 2.00E-16 | LZ_CD9 | DCTPP1 |
| 1.68E-26 | -0.278690878 | 0.637 | 0.681 | 3.02E-22 | LZ_CD9 | CBX3 |
| 8.63E-12 | -0.279228037 | 0.075 | 0.122 | 1.55E-07 | LZ_CD9 | KNL1 |
| 6.51E-21 | -0.279301354 | 0.258 | 0.366 | 1.17E-16 | LZ_CD9 | PDIA4 |
| 7.82E-39 | -0.279396721 | 0.051 | 0.151 | 1.41E-34 | LZ_CD9 | SLC20A1 |
| 7.71E-20 | -0.279815911 | 0.164 | 0.249 | 1.39E-15 | LZ_CD9 | USP1 |
| 3.95E-40 | -0.28000089 | 0.077 | 0.191 | 7.12E-36 | LZ_CD9 | PGP |
| 4.18E-45 | -0.280579376 | 0.045 | 0.157 | 7.53E-41 | LZ_CD9 | EMC9 |
| 5.02E-24 | -0.280984827 | 0.257 | 0.372 | 9.05E-20 | LZ_CD9 | MTCH2 |
| 8.84E-42 | -0.28148192 | 0.064 | 0.179 | 1.59E-37 | LZ_CD9 | FANCD2 |
| 1.30E-26 | -0.281723576 | 0.177 | 0.287 | 2.35E-22 | LZ_CD9 | MRPL55 |
| 5.13E-21 | -0.283267638 | 0.6 | 0.644 | 9.24E-17 | LZ_CD9 | SNRPD1 |
| 5.10E-34 | -0.287435539 | 0.189 | 0.325 | 9.19E-30 | LZ_CD9 | CBFB |
| 1.64E-42 | -0.288546107 | 0.073 | 0.192 | 2.96E-38 | LZ_CD9 | MAGOHB |
| 2.30E-14 | -0.289524711 | 0.366 | 0.434 | 4.15E-10 | LZ_CD9 | PRDX2 |
| 1.11E-27 | -0.290033235 | 0.253 | 0.38 | 2.00E-23 | LZ_CD9 | NDUFV1 |
| 5.51E-33 | -0.2902984 | 0.129 | 0.245 | 9.93E-29 | LZ_CD9 | TECR |
| 6.00E-48 | -0.291071907 | 0.013 | 0.109 | 1.08E-43 | LZ_CD9 | FANCG |
| 2.82E-13 | -0.291835843 | 0.389 | 0.476 | 5.08E-09 | LZ_CD9 | TLR10 |
| 1.09E-42 | -0.292715111 | 0.131 | 0.272 | 1.96E-38 | LZ_CD9 | NUP37 |
| 1.68E-20 | -0.294735322 | 0.592 | 0.64 | 3.02E-16 | LZ_CD9 | HNRNPF |
| 2.78E-23 | -0.295175599 | 0.342 | 0.447 | 5.01E-19 | LZ_CD9 | VPS29 |
| 9.06E-38 | -0.295643009 | 0.109 | 0.23 | 1.63E-33 | LZ_CD9 | RAD51C |
| 1.44E-48 | -0.29709602 | 0.034 | 0.145 | 2.59E-44 | LZ_CD9 | POLD3 |
| 3.84E-14 | -0.298429046 | 0.682 | 0.733 | 6.91E-10 | LZ_CD9 | HNRNPH1 |
| 4.97E-20 | -0.298439293 | 0.444 | 0.522 | 8.96E-16 | LZ_CD9 | DDX39A |
| 3.83E-17 | -0.298792803 | 0.57 | 0.659 | 6.91E-13 | LZ_CD9 | CSK |
| 2.53E-19 | -0.298812485 | 0.096 | 0.17 | 4.56E-15 | LZ_CD9 | ADGRG5 |
| 1.60E-47 | -0.29930417 | 0.01 | 0.102 | 2.88E-43 | LZ_CD9 | FAM83D |
| 1.75E-32 | -0.299508419 | 0.146 | 0.265 | 3.15E-28 | LZ_CD9 | RFC2 |
| 2.17E-08 | -0.299993895 | 0.192 | 0.244 | 0.000391305 | LZ_CD9 | CDC42SE1 |
| 5.49E-20 | -0.301329014 | 0.349 | 0.443 | 9.90E-16 | LZ_CD9 | CCT6A |
| 9.24E-09 | -0.301675999 | 0.085 | 0.126 | 0.000166537 | LZ_CD9 | FCRL5 |
| 2.79E-42 | -0.301834308 | 0.095 | 0.222 | 5.04E-38 | LZ_CD9 | CSE1L |
| 2.53E-46 | -0.302216324 | 0.052 | 0.169 | 4.55E-42 | LZ_CD9 | NCAPH2 |
| 1.88E-39 | -0.302460582 | 0.116 | 0.245 | 3.39E-35 | LZ_CD9 | MND1 |
| 1.18E-20 | -0.303547764 | 0.263 | 0.364 | 2.12E-16 | LZ_CD9 | CCNG2 |
| 1.64E-39 | -0.303767097 | 0.053 | 0.156 | 2.95E-35 | LZ_CD9 | ARHGAP11B |
| 1.30E-47 | -0.304049694 | 0.068 | 0.195 | 2.34E-43 | LZ_CD9 | EBP |
| 1.32E-59 | -0.304371428 | 0.035 | 0.167 | 2.38E-55 | LZ_CD9 | TEX30 |
| 7.23E-21 | -0.304662865 | 0.066 | 0.136 | 1.30E-16 | LZ_CD9 | CD44 |
| 2.08E-10 | -0.304725428 | 0.31 | 0.379 | 3.75E-06 | LZ_CD9 | JUNB |
| 9.98E-21 | -0.305767257 | 0.14 | 0.228 | 1.80E-16 | LZ_CD9 | PIK3CD |
| 1.25E-25 | -0.306106855 | 0.144 | 0.243 | 2.25E-21 | LZ_CD9 | EBNA1BP2 |
| 2.09E-41 | -0.308205272 | 0.1 | 0.226 | 3.77E-37 | LZ_CD9 | MSH2 |
| 3.19E-46 | -0.309238118 | 0.043 | 0.155 | 5.75E-42 | LZ_CD9 | MTHFD1 |
| 6.95E-58 | -0.309580143 | 0.028 | 0.151 | 1.25E-53 | LZ_CD9 | ACOT7 |
| 1.22E-28 | -0.310460468 | 0.99 | 0.989 | 2.20E-24 | LZ_CD9 | B2M |
| 6.73E-23 | -0.310581589 | 0.578 | 0.652 | 1.21E-18 | LZ_CD9 | CLIC1 |
| 8.43E-22 | -0.310738913 | 0.289 | 0.387 | 1.52E-17 | LZ_CD9 | CYC1 |
| 3.62E-57 | -0.311080204 | 0.024 | 0.143 | 6.52E-53 | LZ_CD9 | DEPDC1B |
| 6.16E-35 | -0.311471337 | 0.042 | 0.132 | 1.11E-30 | LZ_CD9 | MT2A |
| 3.85E-18 | -0.311696469 | 0.234 | 0.329 | 6.95E-14 | LZ_CD9 | FXYD5 |
| 3.69E-26 | -0.312765473 | 0.489 | 0.585 | 6.64E-22 | LZ_CD9 | HNRNPA0 |
| 5.16E-52 | -0.313568724 | 0.005 | 0.1 | 9.29E-48 | LZ_CD9 | ANLN |
| 2.31E-21 | -0.314728667 | 0.455 | 0.54 | 4.17E-17 | LZ_CD9 | PTGES3 |
| 3.13E-41 | -0.314833223 | 0.07 | 0.186 | 5.64E-37 | LZ_CD9 | CMSS1 |
| 1.89E-07 | -0.31531785 | 0.237 | 0.281 | 0.003402365 | LZ_CD9 | RNASET2 |
| 1.10E-27 | -0.317185409 | 0.101 | 0.196 | 1.98E-23 | LZ_CD9 | TAP1 |
| 9.23E-24 | -0.318826916 | 0.119 | 0.21 | 1.66E-19 | LZ_CD9 | HACD2 |
| 1.15E-14 | -0.31887545 | 0.164 | 0.239 | 2.07E-10 | LZ_CD9 | ISG15 |
| 3.77E-61 | -0.319028301 | 0.009 | 0.122 | 6.79E-57 | LZ_CD9 | PKMYT1 |
| 1.21E-22 | -0.319240903 | 0.207 | 0.311 | 2.18E-18 | LZ_CD9 | BPNT1 |
| 6.72E-24 | -0.319523192 | 0.411 | 0.525 | 1.21E-19 | LZ_CD9 | ARPC5L |
| 1.38E-34 | -0.320704055 | 0.12 | 0.236 | 2.49E-30 | LZ_CD9 | BARD1 |
| 5.33E-35 | -0.320847262 | 0.169 | 0.3 | 9.60E-31 | LZ_CD9 | GPI |
| 3.42E-25 | -0.320923112 | 0.338 | 0.452 | 6.16E-21 | LZ_CD9 | CBX5 |
| 1.20E-24 | -0.320950669 | 0.154 | 0.255 | 2.16E-20 | LZ_CD9 | PIM3 |
| 4.51E-28 | -0.321208386 | 0.242 | 0.36 | 8.13E-24 | LZ_CD9 | TIMM10 |
| 2.32E-17 | -0.321447856 | 0.1 | 0.168 | 4.18E-13 | LZ_CD9 | DDIT4 |
| 3.39E-13 | -0.322139685 | 0.693 | 0.712 | 6.12E-09 | LZ_CD9 | NCL |
| 1.26E-21 | -0.323031228 | 0.184 | 0.278 | 2.26E-17 | LZ_CD9 | CCDC85B |
| 1.44E-26 | -0.323270814 | 0.145 | 0.244 | 2.60E-22 | LZ_CD9 | CDK4 |
| 3.08E-28 | -0.323310998 | 0.604 | 0.655 | 5.55E-24 | LZ_CD9 | SNRPB |
| 1.57E-13 | -0.323600516 | 0.551 | 0.582 | 2.84E-09 | LZ_CD9 | UBB |
| 1.95E-39 | -0.323642196 | 0.087 | 0.204 | 3.51E-35 | LZ_CD9 | FAM162A |
| 1.25E-20 | -0.323724455 | 0.112 | 0.193 | 2.25E-16 | LZ_CD9 | TMC8 |
| 1.50E-14 | -0.32469317 | 0.255 | 0.325 | 2.71E-10 | LZ_CD9 | PHB |
| 5.84E-17 | -0.32482084 | 0.346 | 0.44 | 1.05E-12 | LZ_CD9 | VASP |
| 5.33E-21 | -0.324971886 | 0.198 | 0.295 | 9.60E-17 | LZ_CD9 | CHI3L2 |
| 7.97E-60 | -0.325176845 | 0.026 | 0.152 | 1.44E-55 | LZ_CD9 | TCF19 |
| 1.67E-35 | -0.32544854 | 0.162 | 0.291 | 3.00E-31 | LZ_CD9 | HPRT1 |
| 6.42E-27 | -0.325906619 | 0.277 | 0.393 | 1.16E-22 | LZ_CD9 | IMPDH2 |
| 4.82E-44 | -0.327405173 | 0.173 | 0.326 | 8.69E-40 | LZ_CD9 | AKR1B1 |
| 4.12E-33 | -0.327614923 | 0.058 | 0.151 | 7.42E-29 | LZ_CD9 | INSIG1 |
| 6.32E-21 | -0.327631009 | 0.139 | 0.228 | 1.14E-16 | LZ_CD9 | LPCAT1 |
| 2.14E-53 | -0.328810418 | 0.007 | 0.107 | 3.86E-49 | LZ_CD9 | KIF15 |
| 7.56E-41 | -0.329050696 | 0.017 | 0.103 | 1.36E-36 | LZ_CD9 | KIF14 |
| 2.98E-28 | -0.329104876 | 0.812 | 0.796 | 5.38E-24 | LZ_CD9 | RAN |
| 8.83E-50 | -0.329184346 | 0.127 | 0.283 | 1.59E-45 | LZ_CD9 | HMGB3 |
| 3.11E-32 | -0.329517323 | 0.104 | 0.214 | 5.61E-28 | LZ_CD9 | CDC25B |
| 5.80E-52 | -0.330387933 | 0.073 | 0.211 | 1.05E-47 | LZ_CD9 | CHAF1A |
| 4.40E-32 | -0.330476829 | 0.246 | 0.379 | 7.94E-28 | LZ_CD9 | SUMO3 |
| 1.68E-23 | -0.3323802 | 0.344 | 0.46 | 3.03E-19 | LZ_CD9 | CD47 |
| 4.27E-56 | -0.333244443 | 0.066 | 0.208 | 7.70E-52 | LZ_CD9 | FANCI |
| 1.07E-59 | -0.333636414 | 0.022 | 0.144 | 1.93E-55 | LZ_CD9 | NCAPG2 |
| 2.82E-36 | -0.333978381 | 0.394 | 0.53 | 5.08E-32 | LZ_CD9 | ANP32E |
| 1.00E-14 | -0.334243633 | 0.067 | 0.12 | 1.80E-10 | LZ_CD9 | FCER2 |
| 1.92E-27 | -0.334734584 | 0.197 | 0.312 | 3.47E-23 | LZ_CD9 | ANXA6 |
| 5.19E-50 | -0.335183003 | 0.014 | 0.114 | 9.36E-46 | LZ_CD9 | ECT2 |
| 1.80E-30 | -0.335420073 | 0.228 | 0.355 | 3.24E-26 | LZ_CD9 | DRAP1 |
| 6.04E-25 | -0.335907586 | 0.261 | 0.373 | 1.09E-20 | LZ_CD9 | NOP56 |
| 2.51E-12 | -0.336208402 | 0.144 | 0.205 | 4.53E-08 | LZ_CD9 | HOPX |
| 7.47E-61 | -0.336255979 | 0.004 | 0.114 | 1.35E-56 | LZ_CD9 | E2F8 |
| 1.41E-26 | -0.336542567 | 0.184 | 0.294 | 2.53E-22 | LZ_CD9 | HELLS |
| 5.96E-26 | -0.336702639 | 0.344 | 0.448 | 1.07E-21 | LZ_CD9 | LSM4 |
| 2.31E-23 | -0.337637575 | 0.06 | 0.133 | 4.16E-19 | LZ_CD9 | CD1C |
| 3.01E-39 | -0.337749238 | 0.139 | 0.27 | 5.42E-35 | LZ_CD9 | AHCY |
| 1.90E-34 | -0.337880738 | 0.076 | 0.178 | 3.43E-30 | LZ_CD9 | CKAP5 |
| 1.61E-16 | -0.338676532 | 0.052 | 0.107 | 2.90E-12 | LZ_CD9 | PLAC8 |
| 1.84E-25 | -0.339595359 | 0.076 | 0.16 | 3.32E-21 | LZ_CD9 | EMP3 |
| 4.12E-39 | -0.340160845 | 0.139 | 0.27 | 7.42E-35 | LZ_CD9 | VDAC3 |
| 2.09E-23 | -0.340849692 | 0.214 | 0.314 | 3.77E-19 | LZ_CD9 | PDCD5 |
| 1.27E-38 | -0.340953071 | 0.943 | 0.936 | 2.29E-34 | LZ_CD9 | HMGB1 |
| 9.00E-20 | -0.341088345 | 0.06 | 0.123 | 1.62E-15 | LZ_CD9 | SLC2A3 |
| 1.52E-40 | -0.341369521 | 0.045 | 0.146 | 2.73E-36 | LZ_CD9 | SYNGR1 |
| 5.14E-22 | -0.341691274 | 0.286 | 0.388 | 9.25E-18 | LZ_CD9 | SRI |
| 4.89E-64 | -0.341835164 | 0.066 | 0.222 | 8.81E-60 | LZ_CD9 | MIS18A |
| 2.91E-31 | -0.342667244 | 0.363 | 0.484 | 5.24E-27 | LZ_CD9 | ILF2 |
| 9.20E-58 | -0.345417497 | 0.055 | 0.194 | 1.66E-53 | LZ_CD9 | CHEK1 |
| 6.42E-30 | -0.34566779 | 0.302 | 0.431 | 1.16E-25 | LZ_CD9 | SMS |
| 4.57E-27 | -0.346696535 | 0.034 | 0.104 | 8.24E-23 | LZ_CD9 | SLAMF1 |
| 1.53E-39 | -0.348963412 | 0.737 | 0.764 | 2.76E-35 | LZ_CD9 | HMGA1 |
| 1.44E-52 | -0.349360376 | 0.005 | 0.101 | 2.59E-48 | LZ_CD9 | KIF18B |
| 2.80E-52 | -0.350037331 | 0.007 | 0.105 | 5.05E-48 | LZ_CD9 | KIF20A |
| 4.55E-45 | -0.352342885 | 0.134 | 0.281 | 8.21E-41 | LZ_CD9 | MCM6 |
| 2.45E-41 | -0.352687607 | 0.747 | 0.847 | 4.42E-37 | LZ_CD9 | CD53 |
| 9.60E-14 | -0.352937764 | 0.093 | 0.148 | 1.73E-09 | LZ_CD9 | CAMK1 |
| 1.26E-57 | -0.353862368 | 0.02 | 0.136 | 2.28E-53 | LZ_CD9 | KNSTRN |
| 2.74E-33 | -0.354761819 | 0.284 | 0.423 | 4.94E-29 | LZ_CD9 | TFDP1 |
| 2.34E-28 | -0.355741592 | 0.3 | 0.415 | 4.22E-24 | LZ_CD9 | CACYBP |
| 4.12E-16 | -0.356903442 | 0.174 | 0.248 | 7.43E-12 | LZ_CD9 | ICAM3 |
| 3.36E-58 | -0.356936199 | 0.036 | 0.164 | 6.06E-54 | LZ_CD9 | SPDL1 |
| 4.76E-51 | -0.360017313 | 0.019 | 0.124 | 8.58E-47 | LZ_CD9 | E2F2 |
| 7.41E-22 | -0.360037249 | 0.249 | 0.348 | 1.34E-17 | LZ_CD9 | LYL1 |
| 1.27E-20 | -0.361660155 | 0.099 | 0.175 | 2.28E-16 | LZ_CD9 | LGALS9 |
| 2.19E-31 | -0.363707258 | 0.219 | 0.342 | 3.94E-27 | LZ_CD9 | HSPA9 |
| 1.59E-73 | -0.364380809 | 0.031 | 0.183 | 2.86E-69 | LZ_CD9 | RAD51 |
| 6.92E-54 | -0.364693492 | 0.034 | 0.155 | 1.25E-49 | LZ_CD9 | SLC43A3 |
| 2.71E-23 | -0.366246152 | 0.265 | 0.36 | 4.88E-19 | LZ_CD9 | FDPS |
| 4.44E-34 | -0.368078716 | 0.279 | 0.413 | 8.00E-30 | LZ_CD9 | CCDC167 |
| 1.41E-50 | -0.368636591 | 0.086 | 0.224 | 2.54E-46 | LZ_CD9 | POP7 |
| 3.08E-20 | -0.368860919 | 0.408 | 0.491 | 5.54E-16 | LZ_CD9 | CHCHD10 |
| 1.23E-47 | -0.369792564 | 0.119 | 0.264 | 2.21E-43 | LZ_CD9 | EXOSC8 |
| 1.64E-66 | -0.371783046 | 0.022 | 0.156 | 2.95E-62 | LZ_CD9 | CDC45 |
| 3.28E-27 | -0.372512334 | 0.625 | 0.654 | 5.91E-23 | LZ_CD9 | DEK |
| 8.28E-110 | -0.373597811 | 0.985 | 0.986 | 1.49E-105 | LZ_CD9 | PFN1 |
| 3.34E-34 | -0.374128334 | 0.282 | 0.415 | 6.03E-30 | LZ_CD9 | SSRP1 |
| 1.89E-32 | -0.37484278 | 0.251 | 0.387 | 3.40E-28 | LZ_CD9 | PTPN7 |
| 2.21E-54 | -0.375583246 | 0.039 | 0.161 | 3.99E-50 | LZ_CD9 | SVIP |
| 5.04E-66 | -0.376942609 | 0.017 | 0.146 | 9.09E-62 | LZ_CD9 | CDC6 |
| 4.88E-57 | -0.3773501 | 0.032 | 0.156 | 8.80E-53 | LZ_CD9 | CDCA2 |
| 5.32E-39 | -0.378305085 | 0.192 | 0.331 | 9.59E-35 | LZ_CD9 | POLR3K |
| 1.41E-35 | -0.379763601 | 0.252 | 0.382 | 2.54E-31 | LZ_CD9 | EIF4A3 |
| 9.53E-31 | -0.381232147 | 0.447 | 0.568 | 1.72E-26 | LZ_CD9 | PDIA3 |
| 2.29E-44 | -0.382553819 | 0.101 | 0.231 | 4.13E-40 | LZ_CD9 | ATAD2 |
| 2.00E-64 | -0.38313383 | 0.925 | 0.962 | 3.61E-60 | LZ_CD9 | HLA-B |
| 4.42E-35 | -0.383377036 | 0.246 | 0.378 | 7.97E-31 | LZ_CD9 | PSMC3 |
| 6.49E-46 | -0.3854848 | 0.072 | 0.194 | 1.17E-41 | LZ_CD9 | LYAR |
| 2.05E-32 | -0.385560086 | 0.317 | 0.44 | 3.69E-28 | LZ_CD9 | YWHAH |
| 7.27E-59 | -0.388048577 | 0.009 | 0.12 | 1.31E-54 | LZ_CD9 | NEK2 |
| 9.31E-22 | -0.391613723 | 0.394 | 0.505 | 1.68E-17 | LZ_CD9 | PAG1 |
| 3.12E-28 | -0.392889407 | 0.697 | 0.766 | 5.63E-24 | LZ_CD9 | LRMP |
| 2.52E-20 | -0.393730884 | 0.235 | 0.33 | 4.54E-16 | LZ_CD9 | SUB1 |
| 1.42E-66 | -0.394580225 | 0.022 | 0.155 | 2.56E-62 | LZ_CD9 | C21orf58 |
| 1.82E-44 | -0.395269971 | 0.205 | 0.361 | 3.28E-40 | LZ_CD9 | HAT1 |
| 4.50E-34 | -0.395476776 | 0.156 | 0.275 | 8.10E-30 | LZ_CD9 | ALYREF |
| 4.34E-58 | -0.395625219 | 0.113 | 0.275 | 7.82E-54 | LZ_CD9 | MAD2L2 |
| 1.20E-48 | -0.396830319 | 0.076 | 0.206 | 2.17E-44 | LZ_CD9 | CCDC18 |
| 9.13E-24 | -0.39742652 | 0.387 | 0.478 | 1.65E-19 | LZ_CD9 | PRMT1 |
| 6.71E-22 | -0.398618556 | 0.282 | 0.388 | 1.21E-17 | LZ_CD9 | PIP4K2A |
| 2.97E-38 | -0.398751114 | 0.207 | 0.346 | 5.35E-34 | LZ_CD9 | EZH2 |
| 1.01E-56 | -0.399083469 | 0.047 | 0.177 | 1.81E-52 | LZ_CD9 | ARHGAP11A |
| 3.28E-65 | -0.399727156 | 0.035 | 0.175 | 5.90E-61 | LZ_CD9 | CEP55 |
| 5.42E-33 | -0.400911496 | 0.581 | 0.642 | 9.77E-29 | LZ_CD9 | SNRPG |
| 1.67E-52 | -0.401560002 | 0.826 | 0.847 | 3.01E-48 | LZ_CD9 | HSP90AA1 |
| 1.25E-24 | -0.401947773 | 0.104 | 0.191 | 2.26E-20 | LZ_CD9 | PHACTR1 |
| 7.80E-59 | -0.402433468 | 0.105 | 0.264 | 1.41E-54 | LZ_CD9 | CENPU |
| 2.24E-51 | -0.403856214 | 0.067 | 0.195 | 4.04E-47 | LZ_CD9 | ACAT2 |
| 5.63E-60 | -0.403932812 | 0.082 | 0.236 | 1.02E-55 | LZ_CD9 | RFC3 |
| 6.14E-63 | -0.405293514 | 0.013 | 0.133 | 1.11E-58 | LZ_CD9 | TTK |
| 4.01E-66 | -0.405470511 | 0.022 | 0.154 | 7.23E-62 | LZ_CD9 | SPAG5 |
| 4.58E-68 | -0.405552255 | 0.041 | 0.188 | 8.25E-64 | LZ_CD9 | TUBG1 |
| 2.14E-66 | -0.405750723 | 0.057 | 0.212 | 3.85E-62 | LZ_CD9 | CCDC34 |
| 2.67E-32 | -0.405967228 | 0.366 | 0.486 | 4.81E-28 | LZ_CD9 | GPX4 |
| 1.89E-16 | -0.411029123 | 0.474 | 0.525 | 3.41E-12 | LZ_CD9 | HSPD1 |
| 5.07E-26 | -0.411248547 | 0.431 | 0.512 | 9.13E-22 | LZ_CD9 | PGAM1 |
| 7.31E-28 | -0.414749964 | 0.114 | 0.212 | 1.32E-23 | LZ_CD9 | CIITA |
| 1.34E-37 | -0.416701399 | 0.211 | 0.345 | 2.41E-33 | LZ_CD9 | GLRX |
| 3.25E-41 | -0.417473131 | 0.257 | 0.403 | 5.85E-37 | LZ_CD9 | HSPB11 |
| 3.47E-52 | -0.41842839 | 0.094 | 0.236 | 6.25E-48 | LZ_CD9 | DBF4 |
| 2.43E-29 | -0.419954808 | 0.138 | 0.241 | 4.38E-25 | LZ_CD9 | SRM |
| 2.84E-34 | -0.419990624 | 0.255 | 0.385 | 5.11E-30 | LZ_CD9 | DHFR |
| 1.85E-23 | -0.423148698 | 0.062 | 0.134 | 3.34E-19 | LZ_CD9 | SEMA7A |
| 4.00E-15 | -0.423330336 | 0.976 | 0.984 | 7.20E-11 | LZ_CD9 | MALAT1 |
| 1.00E-49 | -0.426026247 | 0.109 | 0.252 | 1.81E-45 | LZ_CD9 | TMEM109 |
| 1.28E-25 | -0.426186041 | 0.068 | 0.146 | 2.31E-21 | LZ_CD9 | ACP5 |
| 1.35E-67 | -0.427816006 | 0.028 | 0.167 | 2.44E-63 | LZ_CD9 | SPC24 |
| 2.86E-26 | -0.4314208 | 0.204 | 0.307 | 5.15E-22 | LZ_CD9 | CRIP1 |
| 8.18E-45 | -0.432491291 | 0.165 | 0.308 | 1.47E-40 | LZ_CD9 | BID |
| 8.86E-30 | -0.433477833 | 0.426 | 0.549 | 1.60E-25 | LZ_CD9 | SYNGR2 |
| 2.60E-62 | -0.437390849 | 0.095 | 0.256 | 4.69E-58 | LZ_CD9 | PHF19 |
| 1.89E-27 | -0.438747991 | 0.392 | 0.483 | 3.40E-23 | LZ_CD9 | ATP5G1 |
| 3.70E-82 | -0.441641519 | 0.007 | 0.152 | 6.67E-78 | LZ_CD9 | MELK |
| 4.61E-66 | -0.442092682 | 0.099 | 0.268 | 8.30E-62 | LZ_CD9 | YEATS4 |
| 9.09E-42 | -0.442806104 | 0.198 | 0.344 | 1.64E-37 | LZ_CD9 | SMC1A |
| 4.04E-49 | -0.446416152 | 0.127 | 0.271 | 7.28E-45 | LZ_CD9 | MANF |
| 5.92E-69 | -0.446558774 | 0.038 | 0.185 | 1.07E-64 | LZ_CD9 | RACGAP1 |
| 1.48E-15 | -0.446733025 | 0.471 | 0.524 | 2.66E-11 | LZ_CD9 | HSPE1 |
| 1.44E-81 | -0.447011577 | 0.006 | 0.149 | 2.60E-77 | LZ_CD9 | ESCO2 |
| 1.37E-15 | -0.447495274 | 0.815 | 0.791 | 2.48E-11 | LZ_CD9 | HSP90AB1 |
| 2.66E-42 | -0.448456514 | 0.693 | 0.797 | 4.80E-38 | LZ_CD9 | HLA-E |
| 1.64E-76 | -0.448715352 | 0.793 | 0.83 | 2.96E-72 | LZ_CD9 | PKM |
| 8.70E-77 | -0.45116776 | 0.057 | 0.227 | 1.57E-72 | LZ_CD9 | CENPK |
| 1.23E-67 | -0.451421162 | 0.09 | 0.259 | 2.21E-63 | LZ_CD9 | RNASEH2A |
| 2.94E-72 | -0.451437674 | 0.068 | 0.234 | 5.30E-68 | LZ_CD9 | LRR1 |
| 4.77E-45 | -0.452152916 | 0.224 | 0.375 | 8.59E-41 | LZ_CD9 | MCM3 |
| 7.88E-50 | -0.454865048 | 0.787 | 0.866 | 1.42E-45 | LZ_CD9 | HLA-C |
| 2.74E-70 | -0.455914062 | 0.02 | 0.156 | 4.94E-66 | LZ_CD9 | UHRF1 |
| 1.07E-40 | -0.45812459 | 0.135 | 0.264 | 1.93E-36 | LZ_CD9 | SNX22 |
| 6.55E-25 | -0.458803087 | 0.351 | 0.454 | 1.18E-20 | LZ_CD9 | HSPA5 |
| 3.49E-64 | -0.458991603 | 0.141 | 0.318 | 6.28E-60 | LZ_CD9 | BCL2L12 |
| 3.38E-42 | -0.459238129 | 0.063 | 0.173 | 6.08E-38 | LZ_CD9 | SAMD1 |
| 2.59E-33 | -0.46005151 | 0.674 | 0.691 | 4.66E-29 | LZ_CD9 | HMGB2 |
| 1.14E-59 | -0.461508253 | 0.047 | 0.183 | 2.05E-55 | LZ_CD9 | NPM3 |
| 5.33E-47 | -0.462236964 | 0.456 | 0.6 | 9.61E-43 | LZ_CD9 | PSMB9 |
| 1.20E-67 | -0.464038829 | 0.091 | 0.26 | 2.16E-63 | LZ_CD9 | CDCA4 |
| 2.74E-27 | -0.464335661 | 0.626 | 0.662 | 4.93E-23 | LZ_CD9 | STMN1 |
| 5.12E-29 | -0.466721857 | 0.099 | 0.193 | 9.22E-25 | LZ_CD9 | PARP14 |
| 6.62E-47 | -0.468178055 | 0.038 | 0.147 | 1.19E-42 | LZ_CD9 | LY6E |
| 2.04E-77 | -0.469408856 | 0.739 | 0.805 | 3.67E-73 | LZ_CD9 | ALDOA |
| 8.29E-26 | -0.470948025 | 0.252 | 0.366 | 1.49E-21 | LZ_CD9 | PLEK |
| 1.28E-52 | -0.473684725 | 0.117 | 0.271 | 2.31E-48 | LZ_CD9 | MCM4 |
| 1.20E-78 | -0.476263377 | 0.061 | 0.235 | 2.17E-74 | LZ_CD9 | ORC6 |
| 1.95E-54 | -0.476378466 | 0.104 | 0.251 | 3.52E-50 | LZ_CD9 | DNPH1 |
| 7.45E-65 | -0.478290486 | 0.102 | 0.269 | 1.34E-60 | LZ_CD9 | SHCBP1 |
| 1.19E-48 | -0.478658534 | 0.505 | 0.609 | 2.14E-44 | LZ_CD9 | H2AFY |
| 7.56E-41 | -0.48025177 | 0.122 | 0.249 | 1.36E-36 | LZ_CD9 | HLA-F |
| 1.27E-63 | -0.480287332 | 0.134 | 0.307 | 2.29E-59 | LZ_CD9 | VRK1 |
| 5.28E-47 | -0.481706644 | 0.156 | 0.301 | 9.52E-43 | LZ_CD9 | PAICS |
| 1.94E-68 | -0.482203454 | 0.058 | 0.215 | 3.50E-64 | LZ_CD9 | BRCA1 |
| 3.56E-42 | -0.483116193 | 0.149 | 0.284 | 6.41E-38 | LZ_CD9 | HSP90B1 |
| 4.78E-58 | -0.483443267 | 0.106 | 0.26 | 8.61E-54 | LZ_CD9 | DNAJC9 |
| 7.58E-65 | -0.483693991 | 0.096 | 0.263 | 1.37E-60 | LZ_CD9 | NDC1 |
| 8.90E-88 | -0.485201142 | 0.028 | 0.199 | 1.60E-83 | LZ_CD9 | RAD51AP1 |
| 3.63E-72 | -0.487506705 | 0.072 | 0.239 | 6.54E-68 | LZ_CD9 | RFC4 |
| 9.34E-78 | -0.489003618 | 0.012 | 0.155 | 1.68E-73 | LZ_CD9 | CKAP2L |
| 4.48E-50 | -0.493054372 | 0.252 | 0.409 | 8.07E-46 | LZ_CD9 | SNRNP25 |
| 1.09E-104 | -0.495690231 | 0.981 | 0.994 | 1.96E-100 | LZ_CD9 | HLA-DRA |
| 5.06E-65 | -0.497222689 | 0.769 | 0.868 | 9.12E-61 | LZ_CD9 | MARCKSL1 |
| 1.49E-119 | -0.498642865 | 0.988 | 0.989 | 2.68E-115 | LZ_CD9 | PTMA |
| 2.10E-36 | -0.502416723 | 0.032 | 0.118 | 3.78E-32 | LZ_CD9 | MIR155HG |
| 1.53E-43 | -0.502437612 | 0.399 | 0.528 | 2.75E-39 | LZ_CD9 | NASP |
| 1.00E-56 | -0.502538822 | 0.093 | 0.24 | 1.81E-52 | LZ_CD9 | SDF2L1 |
| 6.30E-16 | -0.504832452 | 0.196 | 0.273 | 1.13E-11 | LZ_CD9 | DUSP2 |
| 8.93E-34 | -0.506799493 | 0.33 | 0.441 | 1.61E-29 | LZ_CD9 | PDIA6 |
| 5.12E-49 | -0.513633998 | 0.185 | 0.339 | 9.22E-45 | LZ_CD9 | TACC3 |
| 4.99E-74 | -0.514637987 | 0.028 | 0.177 | 8.99E-70 | LZ_CD9 | BUB1B |
| 5.89E-67 | -0.514987189 | 0.156 | 0.342 | 1.06E-62 | LZ_CD9 | SAE1 |
| 3.44E-75 | -0.516286574 | 0.089 | 0.27 | 6.20E-71 | LZ_CD9 | LIG1 |
| 8.23E-47 | -0.517428666 | 0.262 | 0.407 | 1.48E-42 | LZ_CD9 | HNRNPAB |
| 7.68E-40 | -0.517992794 | 0.303 | 0.443 | 1.38E-35 | LZ_CD9 | CD72 |
| 8.84E-90 | -0.518480072 | 0.018 | 0.184 | 1.59E-85 | LZ_CD9 | NCAPH |
| 6.13E-40 | -0.518634036 | 0.239 | 0.381 | 1.11E-35 | LZ_CD9 | ITGB2 |
| 3.55E-148 | -0.529798365 | 0.999 | 0.999 | 6.39E-144 | LZ_CD9 | CD74 |
| 2.69E-80 | -0.531145242 | 0.01 | 0.155 | 4.84E-76 | LZ_CD9 | CCNF |
| 1.87E-75 | -0.535127397 | 0.017 | 0.159 | 3.36E-71 | LZ_CD9 | DEPDC1 |
| 2.10E-84 | -0.536237562 | 0.008 | 0.157 | 3.79E-80 | LZ_CD9 | KIF23 |
| 1.14E-71 | -0.543971524 | 0.033 | 0.179 | 2.05E-67 | LZ_CD9 | MXD3 |
| 3.95E-83 | -0.544217461 | 0.089 | 0.28 | 7.11E-79 | LZ_CD9 | CENPW |
| 2.31E-49 | -0.544311643 | 0.289 | 0.448 | 4.16E-45 | LZ_CD9 | ABRACL |
| 3.41E-99 | -0.546024801 | 0.014 | 0.191 | 6.14E-95 | LZ_CD9 | CDCA5 |
| 5.30E-63 | -0.547153092 | 0.196 | 0.373 | 9.56E-59 | LZ_CD9 | IDH2 |
| 6.58E-47 | -0.54808996 | 0.236 | 0.392 | 1.19E-42 | LZ_CD9 | CR2 |
| 5.26E-65 | -0.55592728 | 0.234 | 0.413 | 9.48E-61 | LZ_CD9 | LMNB1 |
| 1.11E-76 | -0.559171694 | 0.13 | 0.321 | 1.99E-72 | LZ_CD9 | GMNN |
| 3.61E-69 | -0.563969862 | 0.742 | 0.778 | 6.51E-65 | LZ_CD9 | ENO1 |
| 1.66E-44 | -0.567504024 | 0.118 | 0.244 | 2.98E-40 | LZ_CD9 | TUBA1C |
| 3.16E-81 | -0.572444177 | 0.12 | 0.316 | 5.70E-77 | LZ_CD9 | DTYMK |
| 4.68E-89 | -0.579448044 | 0.07 | 0.263 | 8.44E-85 | LZ_CD9 | TMEM106C |
| 6.04E-86 | -0.581345992 | 0.054 | 0.234 | 1.09E-81 | LZ_CD9 | FBXO5 |
| 1.79E-34 | -0.581418269 | 0.432 | 0.532 | 3.23E-30 | LZ_CD9 | EIF5A |
| 4.68E-72 | -0.582871041 | 0.077 | 0.244 | 8.43E-68 | LZ_CD9 | FAM111A |
| 7.29E-50 | -0.589571977 | 0.184 | 0.338 | 1.31E-45 | LZ_CD9 | GMDS |
| 1.02E-62 | -0.591236852 | 0.105 | 0.266 | 1.84E-58 | LZ_CD9 | PRC1 |
| 1.65E-18 | -0.595155764 | 0.329 | 0.417 | 2.97E-14 | LZ_CD9 | CD83 |
| 1.76E-55 | -0.595174601 | 0.261 | 0.433 | 3.18E-51 | LZ_CD9 | IER2 |
| 2.10E-43 | -0.596987444 | 0.288 | 0.42 | 3.79E-39 | LZ_CD9 | CALR |
| 2.92E-56 | -0.601951982 | 0.312 | 0.484 | 5.26E-52 | LZ_CD9 | IGHD |
| 1.60E-32 | -0.603903103 | 0.426 | 0.502 | 2.88E-28 | LZ_CD9 | MIF |
| 5.99E-67 | -0.608554137 | 0.184 | 0.37 | 1.08E-62 | LZ_CD9 | BIRC5 |
| 3.11E-95 | -0.608678966 | 0.04 | 0.228 | 5.60E-91 | LZ_CD9 | CLSPN |
| 1.08E-50 | -0.61141594 | 0.268 | 0.423 | 1.95E-46 | LZ_CD9 | TMPO |
| 1.03E-95 | -0.613993586 | 0.845 | 0.922 | 1.85E-91 | LZ_CD9 | HLA-DPB1 |
| 5.76E-22 | -0.620373623 | 0.163 | 0.246 | 1.04E-17 | LZ_CD9 | BCL2A1 |
| 7.26E-79 | -0.625056444 | 0.049 | 0.214 | 1.31E-74 | LZ_CD9 | KIF11 |
| 5.95E-98 | -0.625537615 | 0.932 | 0.962 | 1.07E-93 | LZ_CD9 | MS4A1 |
| 5.85E-42 | -0.629207565 | 0.263 | 0.408 | 1.06E-37 | LZ_CD9 | LMO2 |
| 2.97E-89 | -0.630083238 | 0.547 | 0.673 | 5.36E-85 | LZ_CD9 | SRSF2 |
| 1.91E-24 | -0.630763009 | 0.208 | 0.292 | 3.44E-20 | LZ_CD9 | UBE2S |
| 3.81E-88 | -0.633330972 | 0.027 | 0.194 | 6.87E-84 | LZ_CD9 | BUB1 |
| 3.27E-66 | -0.635225204 | 0.401 | 0.55 | 5.90E-62 | LZ_CD9 | BUB3 |
| 2.38E-67 | -0.636284834 | 0.284 | 0.456 | 4.29E-63 | LZ_CD9 | SIVA1 |
| 2.36E-71 | -0.643859061 | 0.018 | 0.155 | 4.25E-67 | LZ_CD9 | PIF1 |
| 6.28E-55 | -0.645736562 | 0.343 | 0.49 | 1.13E-50 | LZ_CD9 | PSME2 |
| 4.12E-108 | -0.647250628 | 0.741 | 0.855 | 7.42E-104 | LZ_CD9 | HLA-A |
| 2.66E-61 | -0.650237061 | 0.07 | 0.218 | 4.80E-57 | LZ_CD9 | CENPE |
| 3.41E-81 | -0.656780337 | 0.037 | 0.201 | 6.15E-77 | LZ_CD9 | CENPA |
| 6.67E-85 | -0.659860643 | 0.073 | 0.257 | 1.20E-80 | LZ_CD9 | CKAP2 |
| 5.54E-93 | -0.659986173 | 0.053 | 0.24 | 9.98E-89 | LZ_CD9 | UBE2T |
| 8.55E-82 | -0.670951544 | 0.305 | 0.52 | 1.54E-77 | LZ_CD9 | PTPN6 |
| 8.83E-111 | -0.671774993 | 0.021 | 0.218 | 1.59E-106 | LZ_CD9 | NCAPG |
| 1.83E-142 | -0.676165723 | 0.793 | 0.911 | 3.30E-138 | LZ_CD9 | HLA-DPA1 |
| 8.45E-82 | -0.677830234 | 0.649 | 0.792 | 1.52E-77 | LZ_CD9 | HLA-DQA1 |
| 1.22E-49 | -0.680438236 | 0.288 | 0.426 | 2.20E-45 | LZ_CD9 | NME1 |
| 2.76E-110 | -0.685317406 | 0.665 | 0.778 | 4.97E-106 | LZ_CD9 | HSPA8 |
| 2.31E-56 | -0.685583204 | 0.143 | 0.306 | 4.17E-52 | LZ_CD9 | CENPF |
| 3.98E-97 | -0.6883226 | 0.789 | 0.836 | 7.17E-93 | LZ_CD9 | CALM2 |
| 8.01E-79 | -0.688828739 | 0.206 | 0.398 | 1.44E-74 | LZ_CD9 | CENPM |
| 4.65E-74 | -0.691990506 | 0.144 | 0.332 | 8.37E-70 | LZ_CD9 | CDKN3 |
| 1.43E-93 | -0.692781779 | 0.03 | 0.208 | 2.58E-89 | LZ_CD9 | DLGAP5 |
| 5.15E-23 | -0.697871318 | 0.317 | 0.387 | 9.28E-19 | LZ_CD9 | FABP5 |
| 3.68E-96 | -0.698806915 | 0.114 | 0.333 | 6.64E-92 | LZ_CD9 | KIF20B |
| 2.08E-108 | -0.699225417 | 0.007 | 0.191 | 3.76E-104 | LZ_CD9 | HJURP |
| 1.32E-94 | -0.700404346 | 0.05 | 0.241 | 2.38E-90 | LZ_CD9 | CDCA8 |
| 1.15E-119 | -0.702405212 | 0.024 | 0.238 | 2.08E-115 | LZ_CD9 | PBK |
| 1.86E-121 | -0.703785534 | 0.649 | 0.769 | 3.36E-117 | LZ_CD9 | TPI1 |
| 1.74E-111 | -0.705953762 | 0.022 | 0.223 | 3.14E-107 | LZ_CD9 | KIF2C |
| 1.37E-23 | -0.705984714 | 0.15 | 0.236 | 2.47E-19 | LZ_CD9 | HLA-DRB5 |
| 1.00E-74 | -0.707314124 | 0.342 | 0.513 | 1.81E-70 | LZ_CD9 | PA2G4 |
| 1.06E-78 | -0.708604203 | 0.229 | 0.429 | 1.91E-74 | LZ_CD9 | MCM7 |
| 1.89E-110 | -0.71667698 | 0.076 | 0.302 | 3.40E-106 | LZ_CD9 | KIF22 |
| 3.71E-104 | -0.72017855 | 0.062 | 0.275 | 6.68E-100 | LZ_CD9 | NUF2 |
| 2.72E-119 | -0.722733068 | 0.019 | 0.227 | 4.90E-115 | LZ_CD9 | NDC80 |
| 2.48E-60 | -0.723172992 | 0.181 | 0.346 | 4.46E-56 | LZ_CD9 | TXN |
| 1.70E-108 | -0.735814967 | 0.113 | 0.345 | 3.07E-104 | LZ_CD9 | MAD2L1 |
| 9.66E-64 | -0.738165474 | 0.075 | 0.225 | 1.74E-59 | LZ_CD9 | DHRS9 |
| 1.21E-93 | -0.739075745 | 0.142 | 0.356 | 2.18E-89 | LZ_CD9 | RRM1 |
| 2.24E-90 | -0.785372724 | 0.755 | 0.773 | 4.03E-86 | LZ_CD9 | H2AFZ |
| 3.24E-69 | -0.785436781 | 0.138 | 0.315 | 5.83E-65 | LZ_CD9 | CCNB2 |
| 4.22E-89 | -0.802867743 | 0.144 | 0.344 | 7.60E-85 | LZ_CD9 | H2AFX |
| 4.53E-115 | -0.820882136 | 0.057 | 0.278 | 8.16E-111 | LZ_CD9 | CDCA3 |
| 4.54E-138 | -0.821576488 | 0.019 | 0.254 | 8.18E-134 | LZ_CD9 | SPC25 |
| 3.75E-79 | -0.859072159 | 0.404 | 0.546 | 6.75E-75 | LZ_CD9 | RANBP1 |
| 2.85E-129 | -0.861237359 | 0.107 | 0.365 | 5.14E-125 | LZ_CD9 | SMC2 |
| 7.85E-250 | -0.878408222 | 0.864 | 0.967 | 1.42E-245 | LZ_CD9 | HLA-DRB1 |
| 1.59E-152 | -0.885048522 | 0.034 | 0.297 | 2.86E-148 | LZ_CD9 | ASF1B |
| 1.88E-129 | -0.889515391 | 0.078 | 0.328 | 3.39E-125 | LZ_CD9 | FEN1 |
| 1.23E-105 | -0.890451111 | 0.078 | 0.296 | 2.21E-101 | LZ_CD9 | TPX2 |
| 1.13E-83 | -0.893681832 | 0.095 | 0.284 | 2.04E-79 | LZ_CD9 | CDC20 |
| 5.19E-131 | -0.901392127 | 0.535 | 0.752 | 9.36E-127 | LZ_CD9 | HLA-DQB1 |
| 7.04E-116 | -0.917686835 | 0.197 | 0.439 | 1.27E-111 | LZ_CD9 | CKS1B |
| 3.21E-113 | -0.923063086 | 0.028 | 0.233 | 5.78E-109 | LZ_CD9 | AURKA |
| 1.36E-101 | -0.928466293 | 0.054 | 0.255 | 2.45E-97 | LZ_CD9 | HMMR |
| 1.71E-156 | -0.928663154 | 0.029 | 0.292 | 3.08E-152 | LZ_CD9 | CCNA2 |
| 7.46E-93 | -0.938092905 | 0.051 | 0.239 | 1.34E-88 | LZ_CD9 | ASPM |
| 2.49E-157 | -0.94601606 | 0.062 | 0.339 | 4.50E-153 | LZ_CD9 | ZWINT |
| 3.47E-141 | -0.951626131 | 0.026 | 0.266 | 6.26E-137 | LZ_CD9 | GTSE1 |
| 6.80E-114 | -0.962883898 | 0.27 | 0.496 | 1.23E-109 | LZ_CD9 | SMC4 |
| 1.20E-74 | -0.964999771 | 0.352 | 0.526 | 2.16E-70 | LZ_CD9 | ARL6IP1 |
| 3.97E-104 | -0.989127288 | 0.039 | 0.233 | 7.15E-100 | LZ_CD9 | PLK1 |
| 6.97E-139 | -0.989997254 | 0.512 | 0.692 | 1.26E-134 | LZ_CD9 | LDHA |
| 3.29E-99 | -1.002755309 | 0.13 | 0.343 | 5.94E-95 | LZ_CD9 | CCNB1 |
| 5.09E-146 | -1.032314281 | 0.042 | 0.297 | 9.17E-142 | LZ_CD9 | KIFC1 |
| 1.59E-134 | -1.040277258 | 0.121 | 0.38 | 2.87E-130 | LZ_CD9 | TK1 |
| 5.51E-126 | -1.057736513 | 0.171 | 0.419 | 9.92E-122 | LZ_CD9 | PCNA |
| 7.72E-118 | -1.080890675 | 0.463 | 0.608 | 1.39E-113 | LZ_CD9 | DUT |
| 4.28E-129 | -1.139361012 | 0.135 | 0.392 | 7.71E-125 | LZ_CD9 | MKI67 |
| 7.54E-153 | -1.139757439 | 0.085 | 0.357 | 1.36E-148 | LZ_CD9 | TYMS |
| 3.53E-140 | -1.199842737 | 0.288 | 0.529 | 6.36E-136 | LZ_CD9 | CKS2 |
| 4.81E-118 | -1.20646424 | 0.159 | 0.394 | 8.67E-114 | LZ_CD9 | KPNA2 |
| 1.85E-140 | -1.239264035 | 0.195 | 0.451 | 3.33E-136 | LZ_CD9 | NUSAP1 |
| 3.04E-120 | -1.263341772 | 0.235 | 0.455 | 5.48E-116 | LZ_CD9 | TUBB4B |
| 5.77E-179 | -1.267329921 | 0.051 | 0.348 | 1.04E-174 | LZ_CD9 | RRM2 |
| 3.04E-182 | -1.287038974 | 0.065 | 0.368 | 5.48E-178 | LZ_CD9 | AURKB |
| 1.37E-210 | -1.415752729 | 0.63 | 0.744 | 2.47E-206 | LZ_CD9 | TUBB |
| 2.77E-88 | -1.465566297 | 0.109 | 0.295 | 4.99E-84 | LZ_CD9 | UBE2C |
| 3.52E-200 | -1.561326362 | 0.055 | 0.372 | 6.35E-196 | LZ_CD9 | CDK1 |
| 5.23E-145 | -1.564623236 | 0.118 | 0.385 | 9.43E-141 | LZ_CD9 | TOP2A |
| 1.31E-95 | -1.693836541 | 0.421 | 0.542 | 2.35E-91 | LZ_CD9 | TUBA1B |
| 5.92E-123 | -2.624786877 | 0.413 | 0.576 | 1.07E-118 | LZ_CD9 | HIST1H4C |
| 0 | 1.173523601 | 0.765 | 0.693 | 0 | LZ_FCRL3 | SMIM14 |

Table S3. Regulons of each co-expression module in the gene regulatory network.

| Regulon | Cluster |
| --- | --- |
| STAT2 | M2 |
| STAT3 | M2 |
| FOSB | M2 |
| NFE2L2 | M2 |
| ATF3 | M2 |
| MAFA | M2 |
| STAT1 | M2 |
| IRF1 | M2 |
| KLF2 | M2 |
| PML | M2 |
| NFIL3 | M2 |
| IRF9 | M2 |
| ETV6 | M2 |
| BCL3 | M2 |
| BHLHE40 | M2 |
| IRF5 | M2 |
| ELK1 | M2 |
| STAT5A | M2 |
| KDM2B | M2 |
| IRF4 | M2 |
| CHD1 | M2 |
| ARID3A | M2 |
| TCF7 | M2 |
| BRF2 | M2 |
| MYB | M2 |
| FOSL2 | M2 |
| ASCL1 | M2 |
| RFX2 | M2 |
| STAT6 | M2 |
| RELA | M2 |
| NFKB1 | M2 |
| NFKB2 | M2 |
| HIVEP3 | M2 |
| EGR3 | M2 |
| DUSP22 | M2 |
| ZBTB7A | M2 |
| KDM5A | M1 |
| ETS1 | M1 |
| JUND | M1 |
| FOXN3 | M1 |
| TAF7 | M1 |
| RBBP5 | M1 |
| ATF2 | M1 |
| BCL11A | M1 |
| ELK3 | M1 |
| EGR1 | M1 |
| FOXP1 | M1 |
| BACH1 | M1 |
| KLF6 | M1 |
| MAX | M1 |
| KLF4 | M1 |
| ZNF143 | M1 |
| GTF2F1 | M1 |
| GABPA | M1 |
| PBX3 | M1 |
| ZBTB7B | M1 |
| SETDB1 | M1 |
| NR2C2 | M1 |
| SP2 | M1 |
| E2F6 | M1 |
| BRF1 | M1 |
| ETV4 | M1 |
| FOXO3 | M1 |
| NFATC1 | M1 |
| ELF2 | M1 |
| TCF7L2 | M1 |
| CREB1 | M1 |
| TAF1 | M1 |
| SIN3A | M1 |
| SREBF2 | M1 |
| ZMIZ1 | M1 |
| HCFC1 | M1 |
| SP1 | M1 |
| E2F3 | M1 |
| TRIM28 | M1 |
| KLF16 | M1 |
| POLR2A | M1 |
| BCLAF1 | M1 |
| RB1 | M1 |
| EP300 | M1 |
| SRF | M1 |
| RCOR1 | M1 |
| IKZF1 | M1 |
| KLF15 | M1 |
| SP3 | M1 |
| SMARCC2 | M1 |
| SOX11 | M1 |
| ZNF23 | M1 |
| ETV3 | M1 |
| PHF8 | M1 |
| SREBF1 | M1 |
| ELK4 | M1 |
| IKZF3 | M1 |
| TCF12 | M1 |
| ZBTB17 | M1 |
| RFX3 | M1 |
| NHLH1 | M1 |
| GTF3C2 | M1 |
| HOXA2 | M1 |
| MXD1 | M1 |
| ZFX | M1 |
| HOXB7 | M1 |
| HOXA3 | M1 |
| ZNF557 | M1 |
| NPAS2 | M1 |
| FOXP4 | M1 |
| NR2C1 | M1 |
| ATF6 | M1 |
| HINFP | M1 |
| ZNF471 | M1 |
| ETV1 | M1 |
| TBX10 | M1 |
| HIVEP2 | M1 |
| HOXA4 | M1 |
| ZNF606 | M1 |
| MEIS1 | M1 |
| WRNIP1 | M1 |
| ZNF467 | M1 |
| PPARD | M1 |
| NRF1 | M1 |
| IL21 | M1 |
| GPD1 | M1 |
| TEF | M1 |
| DMRTA2 | M1 |
| POU5F1 | M1 |
| ZNF519 | M1 |
| MXI1 | M1 |
| ADNP2 | M1 |
| ARNT2 | M1 |
| GRHL2 | M1 |
| ZNF444 | M1 |
| NFYA | M1 |
| RARG | M1 |
| SETBP1 | M1 |
| TCF4 | M1 |
| ZNF66 | M1 |
| XRCC4 | M1 |
| IRF3 | M1 |
| KLF7 | M1 |
| ELF4 | M1 |
| HOXA10 | M1 |
| SOX9 | M1 |
| SNAI1 | M1 |
| JDP2 | M1 |
| CREB3L2 | M1 |
| ZFHX3 | M1 |
| MAFB | M1 |
| ZFP69B | M1 |
| GLIS3 | M1 |
| HBP1 | M1 |
| GSC | M1 |
| HES6 | M1 |
| NR3C1 | M5 |
| CUX1 | M5 |
| POU2AF1 | M5 |
| MYBL1 | M5 |
| MSI2 | M5 |
| ELF1 | M5 |
| ZEB1 | M5 |
| CTCF | M5 |
| RAD21 | M5 |
| YY1 | M5 |
| EZH2 | M5 |
| TFDP2 | M5 |
| POU2F1 | M5 |
| NFYB | M5 |
| NFYC | M5 |
| PKNOX1 | M5 |
| ATF4 | M5 |
| LHX4 | M5 |
| NR1I2 | M5 |
| THAP1 | M5 |
| E2F2 | M5 |
| BRCA1 | M5 |
| SUZ12 | M5 |
| E2F7 | M5 |
| REL | M6 |
| DDIT3 | M6 |
| CREM | M6 |
| MTA3 | M6 |
| USF1 | M6 |
| ZNF274 | M6 |
| RELB | M6 |
| EGR2 | M6 |
| TEAD3 | M6 |
| IRF2 | M6 |
| FOS | M6 |
| JUN | M6 |
| FLI1 | M6 |
| ETV7 | M6 |
| ETV5 | M6 |
| MEF2A | M6 |
| IRF8 | M6 |
| CREB3 | M6 |
| ZNF524 | M6 |
| ATF6B | M6 |
| KLF3 | M6 |
| XBP1 | M6 |
| JUNB | M6 |
| IRF7 | M6 |
| SPIB | M6 |
| BATF | M4 |
| MYC | M4 |
| GSX2 | M4 |
| ZNF134 | M4 |
| ZNF223 | M4 |
| TP73 | M4 |
| ATF5 | M4 |
| KLF11 | M4 |
| RBM7 | M4 |
| RXRA | M4 |
| MYPOP | M4 |
| MAFG | M4 |
| TP63 | M4 |
| ZNF155 | M4 |
| PAX8 | M4 |
| ZNF823 | M4 |
| ZNF296 | M4 |
| RFXANK | M4 |
| CEBPG | M4 |
| IRF6 | M4 |
| MSC | M4 |
| EOMES | M4 |
| NR2F2 | M4 |
| ELF3 | M4 |
| ATF1 | M4 |
| MESP2 | M4 |
| RXRB | M4 |
| PBX1 | M4 |
| ZNF230 | M4 |
| ZNF580 | M4 |
| ZFP90 | M4 |
| MYF6 | M4 |
| TBP | M4 |
| NFIC | M4 |
| FOXB1 | M4 |
| HOXA6 | M4 |
| TFAP2A | M4 |
| LEF1 | M4 |
| FOXG1 | M4 |
| PPARG | M4 |
| SOX7 | M4 |
| TBX6 | M4 |
| ESRRA | M4 |
| MLX | M4 |
| RBPJ | M4 |
| TFAP4 | M4 |
| E2F4 | M4 |
| POLR3G | M4 |
| HDAC2 | M4 |
| ENO1 | M4 |
| ZNF513 | M3 |
| HOXB2 | M3 |
| HES1 | M3 |
| ASCL2 | M3 |
| RORA | M3 |
| NRL | M3 |
| HES4 | M3 |
| FOXP3 | M3 |
| GCM1 | M3 |
| RARA | M3 |
| RORB | M3 |
| LBX2 | M3 |
| ZNF84 | M3 |
| RFX4 | M3 |
| GTF2B | M3 |
| ZNF165 | M3 |
| USF2 | M3 |
| SPI1 | M3 |
| UBTF | M3 |
| NELFE | M3 |
| FOXP2 | M3 |
| CEBPB | M3 |
| SUPT20H | M3 |
| HMGB1 | M3 |
| POLE4 | M3 |
| ZNF260 | M3 |
| RFX5 | M7 |
| POU3F1 | M7 |
| MAZ | M7 |
| E2F8 | M7 |
| SMARCA4 | M7 |
| MYEF2 | M7 |
| FOXM1 | M7 |
| E2F1 | M7 |
| MYBL2 | M7 |
| TFDP1 | M7 |

Table S4. Proportion of B cell subpopulations in adenoid tissue sorted by Mass Cytometry.

| SubCellType | ANH | AH | TH | logFC_ANH_vs_AH | logFC_ANH_vs_TH | logFC_AH_vs_TH |
| --- | --- | --- | --- | --- | --- | --- |
| Plasma_IgD | 0.261846843 | 0.324378901 | 0.290050711 | -0.30895677 | -0.147581945 | 0.161374825 |
| GC B_CD9 | 0.192077672 | 0.094278546 | 0.123841825 | 1.026688411 | 0.633191193 | -0.393497218 |
| memory B_CD24 | 0.145593219 | 0.13665708 | 0.106712197 | 0.091382954 | 0.448218073 | 0.356835119 |
| Naive B_CD24 | 0.086074847 | 0.072273895 | 0.069826894 | 0.252117051 | 0.301808901 | 0.04969185 |
| Naive B_CD5 | 0.065599805 | 0.067295925 | 0.075392167 | -0.036827623 | -0.20072313 | -0.163895507 |
| memory B_CD27 | 0.073147155 | 0.068688043 | 0.056019264 | 0.090742765 | 0.384878722 | 0.294135957 |
| GC B_CXCR4 | 0.032043043 | 0.044957778 | 0.088144275 | -0.488559568 | -1.459855718 | -0.97129615 |
| memory B_Fas | 0.044896377 | 0.065212336 | 0.051059467 | -0.53854589 | -0.185579448 | 0.352966442 |
| GC B_Fas | 0.025212717 | 0.050618039 | 0.066391225 | -1.005500012 | -1.396840965 | -0.391340953 |
| Naive B_CCR7 | 0.051121214 | 0.050039775 | 0.050168652 | 0.03084681 | 0.027135943 | -0.003710868 |
| Plasma_CCR7 | 0.020926502 | 0.023953616 | 0.020419147 | -0.19491229 | 0.0354086 | 0.23032089 |
| Plasma_CCR4 | 0.001030391 | 0.001254436 | 0.001739873 | -0.283847133 | -0.755789601 | -0.471942467 |
| memory B_CD127 | 0.000430215 | 0.000391629 | 0.000234303 | 0.135570127 | 0.876682863 | 0.741112736 |

Abbreviations: AH, adenoid hypertrophy; ANH, adenoid non-hypertrophy.

Table S5. Differentially expressed genes of CD9^+^ B cell subsets in adenoid hypertrophy and non-hypertrophy.

| p_val | avg_log2FC | pct.1 | pct.2 | p_val_adj | gene |
| --- | --- | --- | --- | --- | --- |
| 5.99E-28 | -1.034786275 | 0.103 | 0.359 | 1.08E-23 | HLA-DRB5 |
| 1.11E-26 | -0.97427034 | 0.042 | 0.228 | 2.00E-22 | LINC01857 |
| 9.99E-24 | -0.282125588 | 0.005 | 0.106 | 1.80E-19 | AC058791.1 |
| 4.26E-23 | -1.028740598 | 0.293 | 0.59 | 7.68E-19 | TSC22D3 |
| 4.27E-23 | -1.108090463 | 0.332 | 0.606 | 7.69E-19 | RACK1 |
| 6.04E-23 | -0.745860287 | 0.039 | 0.202 | 1.09E-18 | SELENOH |
| 4.98E-22 | -0.398067087 | 0.009 | 0.115 | 8.98E-18 | C21orf33 |
| 2.95E-21 | -0.906370953 | 0.261 | 0.535 | 5.32E-17 | JUND |
| 3.15E-21 | -0.641206887 | 0.822 | 0.936 | 5.69E-17 | BTG1 |
| 5.05E-20 | -0.730284216 | 0.284 | 0.577 | 9.10E-16 | KLF6 |
| 2.59E-19 | -0.611976976 | 0.055 | 0.221 | 4.67E-15 | NOP53 |
| 7.80E-19 | 1.371070241 | 0.278 | 0.048 | 1.41E-14 | AC090498.1 |
| 6.97E-18 | -0.536282674 | 0.043 | 0.186 | 1.26E-13 | PKHD1L1 |
| 9.13E-18 | -0.550429335 | 0.895 | 0.965 | 1.65E-13 | HLA-B |
| 1.56E-17 | -0.540733006 | 0.07 | 0.244 | 2.81E-13 | MCUB |
| 2.45E-17 | -0.626307117 | 0.037 | 0.17 | 4.42E-13 | IGHG2 |
| 1.20E-16 | -0.534495091 | 0.066 | 0.231 | 2.16E-12 | ELOC |
| 1.35E-16 | 0.839362784 | 0.707 | 0.478 | 2.43E-12 | ZFAS1 |
| 4.03E-16 | -0.475604364 | 0.278 | 0.548 | 7.26E-12 | RGS1 |
| 4.04E-16 | -0.295125703 | 0.016 | 0.112 | 7.28E-12 | NDUFAF8 |
| 4.46E-16 | -0.565192471 | 0.087 | 0.266 | 8.03E-12 | JPT1 |
| 5.55E-16 | -0.253481466 | 0.028 | 0.144 | 1.00E-11 | UBE2M |
| 6.63E-16 | -0.591151826 | 0.364 | 0.651 | 1.19E-11 | P2RX5 |
| 7.18E-16 | -0.574938963 | 0.075 | 0.24 | 1.29E-11 | COPS9 |
| 9.84E-16 | -0.893485963 | 0.461 | 0.657 | 1.77E-11 | HLA-DQB1 |
| 1.41E-15 | -0.601712281 | 0.176 | 0.413 | 2.55E-11 | RIPOR2 |
| 1.92E-15 | -0.495775292 | 0.202 | 0.442 | 3.46E-11 | FOS |
| 7.12E-15 | -0.56270141 | 0.116 | 0.308 | 1.28E-10 | SELENOT |
| 1.18E-14 | -0.574042314 | 0.111 | 0.295 | 2.12E-10 | SEM1 |
| 2.98E-14 | 0.442578992 | 0.981 | 0.978 | 5.36E-10 | PFN1 |
| 3.01E-14 | -0.343976931 | 0.028 | 0.135 | 5.43E-10 | METTL26 |
| 3.44E-14 | -0.606267988 | 0.207 | 0.433 | 6.19E-10 | RGS2 |
| 4.98E-14 | -0.507720236 | 0.157 | 0.362 | 8.98E-10 | ARID1B |
| 8.58E-14 | -0.655509617 | 0.107 | 0.272 | 1.55E-09 | ELOB |
| 8.92E-14 | -0.610186822 | 0.181 | 0.397 | 1.61E-09 | APH1A |
| 1.06E-13 | -0.947908912 | 0.039 | 0.151 | 1.91E-09 | IGHA2 |
| 1.09E-13 | -0.46137771 | 0.106 | 0.285 | 1.96E-09 | SELENOF |
| 1.21E-13 | -0.419621616 | 0.085 | 0.244 | 2.19E-09 | OTUD1 |
| 1.34E-13 | -0.27361642 | 0.032 | 0.141 | 2.42E-09 | BUD23 |
| 2.20E-13 | -0.488865961 | 0.295 | 0.554 | 3.96E-09 | TCF3 |
| 2.70E-13 | -0.513066939 | 0.162 | 0.365 | 4.87E-09 | DUSP1 |
| 3.79E-13 | -0.472004959 | 0.041 | 0.157 | 6.84E-09 | FAM241A |
| 8.47E-13 | -0.347276076 | 0.044 | 0.163 | 1.53E-08 | SELENOK |
| 9.91E-13 | -0.546386633 | 0.588 | 0.792 | 1.79E-08 | GNAS |
| 1.58E-12 | -0.493610366 | 0.39 | 0.641 | 2.84E-08 | CDV3 |
| 1.71E-12 | -0.302297287 | 0.03 | 0.131 | 3.07E-08 | HIKESHI |
| 2.44E-12 | -0.532446441 | 0.506 | 0.744 | 4.39E-08 | ZFP36L1 |
| 2.45E-12 | -0.412274615 | 0.091 | 0.247 | 4.41E-08 | ARHGAP17 |
| 2.49E-12 | -0.290769752 | 0.996 | 1 | 4.48E-08 | ACTG1 |
| 3.65E-12 | -0.561253643 | 0.117 | 0.285 | 6.57E-08 | PPP1R15A |
| 4.50E-12 | -0.426887956 | 0.032 | 0.131 | 8.12E-08 | WASHC3 |
| 4.85E-12 | -0.278328824 | 0.025 | 0.115 | 8.75E-08 | ATP2B1-AS1 |
| 6.04E-12 | -0.541556569 | 0.159 | 0.353 | 1.09E-07 | CAPNS1 |
| 6.35E-12 | -0.4714103 | 0.116 | 0.285 | 1.14E-07 | WASHC4 |
| 7.81E-12 | -0.411795321 | 0.127 | 0.298 | 1.41E-07 | RAB11FIP1 |
| 8.09E-12 | -0.354854966 | 0.048 | 0.163 | 1.46E-07 | SMIM26 |
| 1.04E-11 | -0.511486981 | 0.092 | 0.237 | 1.87E-07 | MAP3K2 |
| 1.19E-11 | -0.332421853 | 0.048 | 0.163 | 2.15E-07 | AC084033.3 |
| 1.28E-11 | -0.509929981 | 0.176 | 0.369 | 2.31E-07 | IFITM2 |
| 1.30E-11 | 0.50583301 | 0.988 | 0.987 | 2.35E-07 | PTMA |
| 1.91E-11 | 1.077086413 | 0.461 | 0.327 | 3.44E-07 | MIF |
| 2.16E-11 | -0.627548746 | 0.08 | 0.215 | 3.90E-07 | CD69 |
| 2.18E-11 | -0.319377051 | 0.022 | 0.106 | 3.93E-07 | MRPL58 |
| 2.91E-11 | -0.43447181 | 0.073 | 0.205 | 5.24E-07 | KIAA2013 |
| 3.27E-11 | -0.400782588 | 0.069 | 0.199 | 5.90E-07 | STMP1 |
| 3.67E-11 | -0.41382537 | 0.038 | 0.138 | 6.62E-07 | ZNF787 |
| 4.27E-11 | -0.380968855 | 0.123 | 0.288 | 7.69E-07 | FAM102A |
| 4.74E-11 | -0.437986964 | 0.113 | 0.269 | 8.54E-07 | VMP1 |
| 4.90E-11 | -0.373419998 | 0.068 | 0.196 | 8.83E-07 | DMAC1 |
| 5.28E-11 | -0.389566131 | 0.08 | 0.215 | 9.52E-07 | ABHD3 |
| 8.04E-11 | -0.531622433 | 0.137 | 0.301 | 1.45E-06 | GSN |
| 2.37E-10 | -0.432022921 | 0.112 | 0.263 | 4.27E-06 | RTRAF |
| 2.68E-10 | -0.284792526 | 0.097 | 0.24 | 4.83E-06 | PPP6R1 |
| 4.14E-10 | -0.373221302 | 0.191 | 0.378 | 7.46E-06 | TRIR |
| 4.53E-10 | -0.379887866 | 0.144 | 0.314 | 8.16E-06 | ARID5B |
| 4.98E-10 | -0.41540796 | 0.211 | 0.401 | 8.97E-06 | EBLN3P |
| 5.38E-10 | -0.506848703 | 0.13 | 0.285 | 9.69E-06 | PLEKHG1 |
| 6.65E-10 | -0.310207263 | 0.114 | 0.266 | 1.20E-05 | TSPAN33 |
| 7.82E-10 | -0.288768239 | 0.03 | 0.115 | 1.41E-05 | NEPRO |
| 7.88E-10 | -0.457359721 | 0.333 | 0.558 | 1.42E-05 | DTX1 |
| 8.07E-10 | -0.456123587 | 0.121 | 0.269 | 1.45E-05 | HIP1R |
| 8.23E-10 | -0.49463341 | 0.127 | 0.272 | 1.48E-05 | DUSP2 |
| 8.31E-10 | -0.584369829 | 0.725 | 0.872 | 1.50E-05 | CXCR4 |
| 8.45E-10 | -0.489592386 | 0.166 | 0.333 | 1.52E-05 | SGPP1 |
| 8.66E-10 | -0.388340759 | 0.418 | 0.657 | 1.56E-05 | LYN |
| 1.19E-09 | -0.427444316 | 0.117 | 0.263 | 2.15E-05 | EIF3B |
| 1.26E-09 | 0.508335325 | 0.757 | 0.606 | 2.26E-05 | GAPDH |
| 1.46E-09 | -0.295339045 | 0.041 | 0.135 | 2.63E-05 | MAP2K6 |
| 1.62E-09 | -0.496181843 | 0.25 | 0.446 | 2.93E-05 | HSPA5 |
| 1.69E-09 | -0.437467322 | 0.394 | 0.612 | 3.04E-05 | RSRP1 |
| 1.92E-09 | -0.415167239 | 0.04 | 0.131 | 3.45E-05 | ICE1 |
| 2.21E-09 | -0.435350682 | 0.738 | 0.862 | 3.98E-05 | HLA-DPA1 |
| 2.40E-09 | -0.301071655 | 0.105 | 0.247 | 4.32E-05 | NSD3 |
| 2.45E-09 | -0.451887911 | 0.23 | 0.417 | 4.41E-05 | TBC1D1 |
| 2.56E-09 | -0.337061578 | 0.119 | 0.266 | 4.62E-05 | PKN1 |
| 2.68E-09 | -0.397340679 | 0.079 | 0.199 | 4.83E-05 | ZNF91 |
| 2.81E-09 | 0.363456706 | 0.966 | 0.962 | 5.06E-05 | FAU |
| 2.89E-09 | -0.364752096 | 0.258 | 0.455 | 5.21E-05 | SASH3 |
| 3.28E-09 | -0.274981747 | 0.076 | 0.199 | 5.91E-05 | SINHCAF |
| 3.38E-09 | -0.446810854 | 0.446 | 0.657 | 6.09E-05 | SF1 |
| 3.38E-09 | -0.449451758 | 0.224 | 0.404 | 6.09E-05 | JUNB |
| 3.57E-09 | -0.824014826 | 0.305 | 0.481 | 6.44E-05 | TXNIP |
| 3.59E-09 | -0.432350803 | 0.06 | 0.167 | 6.48E-05 | FAM30A |
| 4.32E-09 | -0.314899607 | 0.071 | 0.186 | 7.78E-05 | MGA |
| 4.36E-09 | -0.368118548 | 0.059 | 0.163 | 7.85E-05 | MAP4K5 |
| 4.48E-09 | -0.365608473 | 0.088 | 0.212 | 8.08E-05 | KCMF1 |
| 4.55E-09 | -0.36864979 | 0.091 | 0.218 | 8.21E-05 | DCAF6 |
| 4.69E-09 | -0.307649709 | 0.057 | 0.16 | 8.45E-05 | RYBP |
| 4.78E-09 | -0.423425276 | 0.08 | 0.199 | 8.61E-05 | RUFY1 |
| 4.86E-09 | -0.436609318 | 0.107 | 0.244 | 8.77E-05 | CLPTM1L |
| 5.66E-09 | -0.581269941 | 0.31 | 0.497 | 0.000102072 | ST6GAL1 |
| 5.93E-09 | -0.357285866 | 0.166 | 0.33 | 0.000106915 | STAT6 |
| 6.14E-09 | -0.379232354 | 0.325 | 0.545 | 0.00011062 | GNB1 |
| 6.21E-09 | -0.382093044 | 0.184 | 0.359 | 0.000111874 | BCL2L11 |
| 6.34E-09 | -0.504705363 | 0.294 | 0.506 | 0.000114285 | ZFP36L2 |
| 6.42E-09 | 0.356345779 | 0.984 | 0.978 | 0.000115737 | SERF2 |
| 6.61E-09 | -0.39829594 | 0.098 | 0.224 | 0.000119178 | JUN |
| 7.84E-09 | -0.430867507 | 0.038 | 0.122 | 0.000141294 | PRKAR2B |
| 8.06E-09 | -0.393681917 | 0.476 | 0.712 | 0.000145324 | PLEKHA2 |
| 8.10E-09 | -0.342464472 | 0.24 | 0.426 | 0.000145999 | CD72 |
| 8.34E-09 | 0.378626528 | 0.902 | 0.891 | 0.000150324 | BTF3 |
| 1.08E-08 | -0.326881728 | 0.093 | 0.218 | 0.000194505 | SLC25A38 |
| 1.21E-08 | -0.424911175 | 0.227 | 0.407 | 0.000218729 | ZFP36 |
| 1.23E-08 | -0.31137534 | 0.189 | 0.365 | 0.000220914 | GRK2 |
| 1.25E-08 | -0.342622514 | 0.255 | 0.446 | 0.000225379 | EHD1 |
| 1.37E-08 | -0.49749225 | 0.165 | 0.317 | 0.000246783 | TP53INP1 |
| 1.48E-08 | -0.360908126 | 0.116 | 0.25 | 0.000266005 | LINC00926 |
| 1.49E-08 | -0.290411633 | 0.146 | 0.298 | 0.000267777 | MAP2K2 |
| 1.71E-08 | -0.323713259 | 0.09 | 0.212 | 0.000307466 | CCNB1IP1 |
| 1.74E-08 | -0.479599017 | 0.136 | 0.276 | 0.000313086 | LMO2 |
| 1.92E-08 | -0.400009187 | 0.074 | 0.183 | 0.000345293 | RAB11FIP4 |
| 1.93E-08 | -0.318851594 | 0.098 | 0.224 | 0.000347463 | DDHD1 |
| 2.12E-08 | -0.283231412 | 0.088 | 0.205 | 0.000382237 | INTS10 |
| 2.20E-08 | -0.295728393 | 0.039 | 0.122 | 0.000397319 | AC012236.1 |
| 2.30E-08 | -0.474335665 | 0.089 | 0.205 | 0.000414729 | RNF19B |
| 2.40E-08 | -0.379810612 | 0.478 | 0.67 | 0.000432827 | GNAI2 |
| 2.43E-08 | 0.623906234 | 0.666 | 0.583 | 0.000438255 | TMA7 |
| 2.43E-08 | -0.313518587 | 0.072 | 0.179 | 0.000438407 | ARHGEF6 |
| 2.55E-08 | -0.340690664 | 0.825 | 0.92 | 0.000460355 | CCNI |
| 2.61E-08 | -0.316601181 | 0.113 | 0.247 | 0.00047002 | ZNF75A |
| 2.62E-08 | 0.847738414 | 0.467 | 0.362 | 0.000471863 | SNHG25 |
| 2.66E-08 | -0.528843906 | 0.118 | 0.247 | 0.000479365 | RC3H2 |
| 3.17E-08 | -0.423178233 | 0.181 | 0.343 | 0.0005711 | TGOLN2 |
| 3.32E-08 | -0.354543555 | 0.13 | 0.269 | 0.000597917 | ADNP |
| 3.41E-08 | -0.357561511 | 0.157 | 0.311 | 0.000613736 | ANXA6 |
| 3.45E-08 | -0.476520583 | 0.125 | 0.256 | 0.000621597 | KANK2 |
| 3.61E-08 | -0.345073006 | 0.156 | 0.308 | 0.0006497 | SAFB |
| 3.86E-08 | -0.320810152 | 0.074 | 0.183 | 0.00069649 | R3HDM1 |
| 3.97E-08 | -0.60109847 | 0.168 | 0.311 | 0.000715134 | MGAT1 |
| 4.00E-08 | -0.42742539 | 0.143 | 0.285 | 0.000721741 | ADD1 |
| 4.04E-08 | -0.506007585 | 0.312 | 0.5 | 0.000728792 | PRKCB |
| 4.16E-08 | -0.414526194 | 0.464 | 0.667 | 0.000750225 | NCOA3 |
| 4.58E-08 | -0.370555276 | 0.974 | 0.981 | 0.000825348 | HLA-DRA |
| 4.61E-08 | -0.487984408 | 0.146 | 0.288 | 0.000829942 | VPS4B |
| 5.03E-08 | -0.323489522 | 0.621 | 0.798 | 0.000906903 | TPM3 |
| 5.33E-08 | -0.461536157 | 0.31 | 0.51 | 0.000961408 | LPXN |
| 5.47E-08 | -0.358865281 | 0.293 | 0.497 | 0.000985856 | WAC |
| 5.72E-08 | -0.364586268 | 0.331 | 0.526 | 0.001030477 | YY1 |
| 6.10E-08 | -0.380549461 | 0.142 | 0.282 | 0.001099015 | NRDC |
| 6.23E-08 | 0.675050153 | 0.579 | 0.513 | 0.001122215 | SNRPE |
| 6.75E-08 | -0.371113652 | 0.376 | 0.58 | 0.001216965 | 1-Mar |
| 6.86E-08 | -0.34894239 | 0.243 | 0.413 | 0.001236275 | SOCS1 |
| 7.33E-08 | 0.412999509 | 0.863 | 0.785 | 0.001321535 | COX4I1 |
| 7.59E-08 | -0.281171042 | 0.152 | 0.304 | 0.001367332 | PHF11 |
| 7.64E-08 | -0.327826894 | 0.069 | 0.17 | 0.001376636 | TAF3 |
| 7.73E-08 | -0.413880658 | 0.227 | 0.391 | 0.001393802 | BOD1L1 |
| 8.52E-08 | -0.270443068 | 0.043 | 0.125 | 0.001536214 | MEPCE |
| 9.09E-08 | -0.402964963 | 0.702 | 0.885 | 0.00163802 | POU2AF1 |
| 9.36E-08 | -0.312332066 | 0.237 | 0.417 | 0.001686139 | DEGS1 |
| 9.84E-08 | 0.349760764 | 1 | 1 | 0.00177297 | TMSB4X |
| 1.02E-07 | -0.269967343 | 0.105 | 0.228 | 0.00183174 | SUDS3 |
| 1.03E-07 | -0.375791156 | 0.384 | 0.583 | 0.001853004 | CXXC5 |
| 1.11E-07 | -0.368880559 | 0.457 | 0.676 | 0.001993378 | ORAI2 |
| 1.12E-07 | -0.33895011 | 0.504 | 0.718 | 0.002011717 | TNFRSF13C |
| 1.12E-07 | -0.363123585 | 0.257 | 0.439 | 0.002013354 | C17orf62 |
| 1.23E-07 | 0.718909805 | 0.729 | 0.638 | 0.002218329 | HMGN2 |
| 1.43E-07 | -0.280468373 | 0.052 | 0.141 | 0.002576477 | BRD9 |
| 1.45E-07 | -0.289989652 | 0.128 | 0.26 | 0.002607557 | SYTL1 |
| 1.48E-07 | -0.33568153 | 0.108 | 0.231 | 0.002668565 | LCOR |
| 1.51E-07 | -0.328069195 | 0.73 | 0.885 | 0.002723719 | LCP1 |
| 1.52E-07 | -0.478502626 | 0.507 | 0.683 | 0.002738819 | TAGLN2 |
| 1.72E-07 | -0.356155086 | 0.088 | 0.196 | 0.003107241 | MOAP1 |
| 1.73E-07 | -0.519610867 | 0.549 | 0.724 | 0.003110508 | HERPUD1 |
| 1.80E-07 | -0.340686867 | 0.106 | 0.224 | 0.003247222 | NFATC2 |
| 1.81E-07 | -0.267851894 | 0.062 | 0.157 | 0.003259979 | SAMSN1 |
| 1.87E-07 | -0.299146318 | 0.132 | 0.266 | 0.003368854 | TRANK1 |
| 1.93E-07 | -0.535276757 | 0.493 | 0.683 | 0.003486344 | IL4R |
| 1.96E-07 | -0.256107743 | 0.079 | 0.183 | 0.003526278 | GLTP |
| 1.96E-07 | -0.280621975 | 0.096 | 0.212 | 0.003531361 | PDLIM2 |
| 2.05E-07 | -0.340039912 | 0.075 | 0.176 | 0.00370132 | METRNL |
| 2.12E-07 | 0.392889511 | 0.857 | 0.856 | 0.003823588 | COX7C |
| 2.12E-07 | -0.277977892 | 0.088 | 0.199 | 0.003829081 | FNIP1 |
| 2.16E-07 | -0.431346786 | 0.124 | 0.247 | 0.003896224 | MIR3681HG |
| 2.17E-07 | -0.300732933 | 0.12 | 0.247 | 0.003906531 | SEC31A |
| 2.20E-07 | -0.41306363 | 0.25 | 0.417 | 0.003971633 | NFKBIA |
| 2.35E-07 | -0.273171257 | 0.175 | 0.327 | 0.004237004 | SPTAN1 |
| 2.45E-07 | -0.313725066 | 0.202 | 0.359 | 0.004414842 | EIF1AY |
| 2.55E-07 | -0.298422168 | 0.178 | 0.333 | 0.004598938 | RBM23 |
| 2.60E-07 | -0.383553806 | 0.287 | 0.465 | 0.004694722 | BCL6 |
| 2.61E-07 | -0.27758465 | 0.195 | 0.356 | 0.004708028 | MTERF4 |
| 2.62E-07 | -0.398838241 | 0.043 | 0.122 | 0.00472499 | C11orf68 |
| 2.83E-07 | -0.261605109 | 0.062 | 0.154 | 0.005100164 | CHFR |
| 2.93E-07 | -0.339986331 | 0.48 | 0.683 | 0.005279147 | GRB2 |
| 3.09E-07 | -0.322944982 | 0.239 | 0.407 | 0.005575713 | PMAIP1 |
| 3.15E-07 | -0.272666175 | 0.071 | 0.17 | 0.005682245 | TLK2 |
| 3.17E-07 | -0.302444522 | 0.389 | 0.596 | 0.005705451 | TAF7 |
| 3.18E-07 | -0.342817733 | 0.089 | 0.196 | 0.005736384 | MAVS |
| 3.19E-07 | -0.302833973 | 0.066 | 0.16 | 0.005743876 | SPTLC2 |
| 3.21E-07 | -0.364821689 | 0.174 | 0.321 | 0.005790614 | SNRNP200 |
| 3.37E-07 | -0.339129382 | 0.127 | 0.253 | 0.006078495 | RBCK1 |
| 3.42E-07 | 0.572247365 | 0.934 | 0.936 | 0.006169316 | HMGB1 |
| 3.49E-07 | -0.284764713 | 0.072 | 0.17 | 0.006288415 | ZFX |
| 3.51E-07 | -0.400430631 | 0.084 | 0.186 | 0.006322916 | MOB1B |
| 3.68E-07 | -0.322119625 | 0.218 | 0.375 | 0.006623514 | MBD2 |
| 3.76E-07 | -0.385882958 | 0.14 | 0.266 | 0.006781514 | RABGAP1 |
| 3.99E-07 | 0.348101519 | 0.953 | 0.942 | 0.007188364 | ATP5L |
| 4.14E-07 | -0.315329469 | 0.14 | 0.276 | 0.007459496 | NIP7 |
| 4.17E-07 | -0.332369291 | 0.229 | 0.391 | 0.007510315 | EPC1 |
| 4.32E-07 | 0.412691659 | 0.812 | 0.798 | 0.007792977 | COX6A1 |
| 4.32E-07 | 0.483726332 | 0.792 | 0.782 | 0.007794315 | NDUFA4 |
| 4.43E-07 | -0.291276514 | 0.354 | 0.558 | 0.007983367 | KPNB1 |
| 4.53E-07 | -0.347413057 | 0.044 | 0.122 | 0.00816095 | GTDC1 |
| 4.89E-07 | 0.327320473 | 0.952 | 0.958 | 0.008818426 | NPM1 |
| 4.91E-07 | -0.270581941 | 0.077 | 0.176 | 0.008839927 | CD84 |
| 5.22E-07 | -0.26612902 | 0.108 | 0.224 | 0.009402418 | FMNL1 |
| 5.27E-07 | 0.64789127 | 0.569 | 0.471 | 0.009494008 | UQCRH |
| 5.31E-07 | -0.286650659 | 0.068 | 0.16 | 0.009567729 | GPALPP1 |
| 5.49E-07 | -0.366941496 | 0.35 | 0.545 | 0.009888352 | BANK1 |
| 5.62E-07 | -0.299862618 | 0.13 | 0.256 | 0.010124368 | HECTD1 |
| 5.83E-07 | -0.3105957 | 0.121 | 0.244 | 0.010509383 | SFSWAP |
| 5.88E-07 | -0.34457673 | 0.502 | 0.689 | 0.010594849 | DDX17 |
| 5.89E-07 | -0.280310677 | 0.056 | 0.141 | 0.010607494 | THEMIS2 |
| 6.00E-07 | -0.287100159 | 0.061 | 0.151 | 0.010809936 | TMED5 |
| 6.17E-07 | -0.271210277 | 0.248 | 0.429 | 0.011126345 | CDC42SE2 |
| 6.41E-07 | -0.369034652 | 0.513 | 0.686 | 0.011558401 | FAM107B |
| 6.44E-07 | -0.410997235 | 0.228 | 0.381 | 0.011610275 | SEMA4A |
| 6.46E-07 | -0.269400859 | 0.1 | 0.212 | 0.011644612 | KBTBD8 |
| 6.57E-07 | -0.332876387 | 0.1 | 0.212 | 0.011835237 | SYS1 |
| 7.12E-07 | -0.354024935 | 0.102 | 0.212 | 0.012823827 | ITPR2 |
| 7.30E-07 | 0.285514683 | 0.968 | 0.981 | 0.013148381 | PPIA |
| 7.39E-07 | -0.262099395 | 0.442 | 0.663 | 0.013321704 | WIPF1 |
| 8.17E-07 | -0.420338027 | 0.171 | 0.304 | 0.01471651 | TCERG1 |
| 8.30E-07 | -0.264418715 | 0.168 | 0.314 | 0.014965405 | TRIM28 |
| 8.54E-07 | -0.254659889 | 0.036 | 0.106 | 0.015385542 | FRAT2 |
| 9.10E-07 | -0.282435519 | 0.043 | 0.119 | 0.016405728 | RETREG2 |
| 9.22E-07 | -0.412144797 | 0.271 | 0.436 | 0.016621597 | TACC1 |
| 9.36E-07 | -0.295625343 | 0.07 | 0.163 | 0.016866015 | DPP8 |
| 9.65E-07 | 0.46509117 | 0.64 | 0.532 | 0.017385476 | UBA52 |
| 9.91E-07 | -0.324441489 | 0.234 | 0.394 | 0.017853415 | DDX3X |
| 9.94E-07 | -0.299815082 | 0.128 | 0.25 | 0.017912083 | ZNF791 |
| 9.98E-07 | -0.388433969 | 0.225 | 0.378 | 0.017992684 | CD83 |
| 1.00E-06 | -0.409221122 | 0.147 | 0.269 | 0.018043558 | FGD6 |
| 1.00E-06 | -0.330603874 | 0.174 | 0.314 | 0.018085123 | ITGB2 |
| 1.01E-06 | -0.268308843 | 0.155 | 0.292 | 0.018290181 | STX16 |
| 1.04E-06 | -0.333883179 | 0.137 | 0.263 | 0.018811177 | BPNT1 |
| 1.06E-06 | -0.401161766 | 0.128 | 0.247 | 0.019178307 | YWHAG |
| 1.13E-06 | 0.826224899 | 0.33 | 0.237 | 0.020284541 | MRPL23 |
| 1.13E-06 | -0.270069491 | 0.039 | 0.109 | 0.020288636 | PLEC |
| 1.15E-06 | -0.316244529 | 0.23 | 0.391 | 0.020642474 | FAM120A |
| 1.18E-06 | -0.28432795 | 0.173 | 0.314 | 0.021218969 | LARP1 |
| 1.18E-06 | -0.307041266 | 0.059 | 0.144 | 0.021236314 | ZMAT3 |
| 1.20E-06 | -0.254553243 | 0.096 | 0.202 | 0.021665227 | FAM13B |
| 1.22E-06 | -0.378753347 | 0.102 | 0.208 | 0.021965206 | ATP8A1 |
| 1.22E-06 | -0.286210315 | 0.039 | 0.109 | 0.02202664 | TRAPPC11 |
| 1.26E-06 | -0.31412218 | 0.102 | 0.208 | 0.022626709 | SERTAD2 |
| 1.30E-06 | -0.280552162 | 0.083 | 0.183 | 0.023402417 | STX12 |
| 1.30E-06 | -0.268601295 | 0.173 | 0.314 | 0.023451677 | TMPO |
| 1.36E-06 | -0.347688784 | 0.166 | 0.301 | 0.024426103 | FAM129C |
| 1.36E-06 | -0.327152856 | 0.522 | 0.721 | 0.024561214 | CD81 |
| 1.37E-06 | -0.300977196 | 0.189 | 0.333 | 0.024694526 | FOXO1 |
| 1.44E-06 | -0.259814311 | 0.159 | 0.295 | 0.026020302 | RUBCNL |
| 1.52E-06 | -0.289135793 | 0.314 | 0.494 | 0.027314995 | HLA-DOB |
| 1.55E-06 | -0.254053728 | 0.149 | 0.285 | 0.027854516 | ZCCHC11 |
| 1.57E-06 | 0.624382159 | 0.588 | 0.545 | 0.028353163 | ATP5I |
| 1.62E-06 | -0.284211012 | 0.23 | 0.388 | 0.029106621 | CUL3 |
| 1.67E-06 | -0.291091405 | 0.089 | 0.189 | 0.030021228 | TMX4 |
| 1.69E-06 | -0.350983121 | 0.082 | 0.176 | 0.030369525 | KIAA2026 |
| 1.69E-06 | -0.360206458 | 0.418 | 0.599 | 0.030449306 | PNN |
| 1.71E-06 | -0.290571367 | 0.14 | 0.266 | 0.030749257 | CAB39 |
| 1.71E-06 | -0.312619222 | 0.158 | 0.288 | 0.030854205 | SMG1 |
| 1.72E-06 | -0.263648191 | 0.157 | 0.288 | 0.030915204 | ARHGEF7 |
| 1.72E-06 | -0.256354236 | 0.138 | 0.263 | 0.030960663 | GSDMD |
| 1.73E-06 | -0.370350365 | 0.404 | 0.577 | 0.031137764 | SMAP2 |
| 1.75E-06 | -0.4007192 | 0.243 | 0.397 | 0.031611028 | HP1BP3 |
| 1.79E-06 | -0.277283533 | 0.043 | 0.115 | 0.032322534 | GABARAPL1 |
| 1.80E-06 | -0.339299783 | 0.25 | 0.417 | 0.032408604 | CLINT1 |
| 1.87E-06 | 0.255037065 | 0.961 | 0.968 | 0.033741544 | HNRNPA1 |
| 2.01E-06 | -0.318370891 | 0.125 | 0.244 | 0.036221552 | GNB4 |
| 2.06E-06 | -0.32874112 | 0.228 | 0.378 | 0.037102637 | WSB1 |
| 2.09E-06 | -0.374922422 | 0.06 | 0.141 | 0.037614212 | ELOA |
| 2.12E-06 | 0.711591071 | 0.494 | 0.423 | 0.038258835 | DUT |
| 2.18E-06 | 0.471223335 | 0.7 | 0.647 | 0.039324293 | C14orf2 |
| 2.25E-06 | -0.33725964 | 0.462 | 0.667 | 0.040576795 | SEC14L1 |
| 2.30E-06 | -0.354333388 | 0.405 | 0.583 | 0.041532319 | ETS1 |
| 2.36E-06 | -0.281963396 | 0.757 | 0.885 | 0.042549254 | DDX5 |
| 2.40E-06 | -0.288284124 | 0.073 | 0.163 | 0.043193072 | CSNK1G2 |
| 2.59E-06 | -0.377676674 | 0.072 | 0.16 | 0.04676676 | ARFGEF2 |
| 2.61E-06 | -0.363313085 | 0.185 | 0.321 | 0.046999732 | CTDSP2 |
| 2.61E-06 | -0.291933011 | 0.265 | 0.429 | 0.04708643 | IKZF1 |
| 2.75E-06 | -0.382459518 | 0.114 | 0.218 | 0.049497521 | IFNGR1 |
| 2.86E-06 | -0.25718729 | 0.104 | 0.208 | 0.051598106 | SOS1 |
| 3.10E-06 | -0.27128655 | 0.052 | 0.128 | 0.055874846 | CRYBG1 |
| 3.15E-06 | -0.264852773 | 0.168 | 0.304 | 0.056792155 | GIT2 |
| 3.20E-06 | -0.32814752 | 0.084 | 0.179 | 0.057587688 | GSK3A |
| 3.32E-06 | -0.417975821 | 0.21 | 0.353 | 0.059776535 | CNPPD1 |
| 3.35E-06 | -0.336492305 | 0.361 | 0.542 | 0.060328143 | CCNL1 |
| 3.42E-06 | -0.318329209 | 0.2 | 0.349 | 0.061721329 | EIF4E2 |
| 3.50E-06 | -0.54659407 | 0.183 | 0.304 | 0.063009075 | EML6 |
| 3.52E-06 | -0.281844775 | 0.331 | 0.51 | 0.063487795 | TLR10 |
| 3.66E-06 | -0.395044715 | 0.102 | 0.205 | 0.065937498 | ABLIM1 |
| 3.70E-06 | -0.301560695 | 0.125 | 0.237 | 0.066751605 | VPS13C |
| 3.72E-06 | -0.310800221 | 0.088 | 0.183 | 0.066977247 | MCFD2 |
| 3.79E-06 | -0.334091949 | 0.17 | 0.301 | 0.068341442 | CNOT6L |
| 3.80E-06 | 0.434666728 | 0.792 | 0.801 | 0.068532382 | COX6C |
| 3.86E-06 | -0.277875551 | 0.062 | 0.144 | 0.069518595 | SECISBP2L |
| 3.92E-06 | -0.369093499 | 0.297 | 0.458 | 0.070610573 | NKTR |
| 4.00E-06 | -0.262397879 | 0.502 | 0.74 | 0.072038704 | SEPT9 |
| 4.07E-06 | -0.31440585 | 0.132 | 0.247 | 0.073276645 | ZBTB38 |
| 4.23E-06 | -0.25163692 | 0.136 | 0.256 | 0.076228177 | MBP |
| 4.29E-06 | 0.416957135 | 0.813 | 0.782 | 0.077373203 | EEF1B2 |
| 4.36E-06 | -0.286692157 | 0.191 | 0.33 | 0.078533118 | PHF3 |
| 4.51E-06 | -0.409913442 | 0.346 | 0.519 | 0.081238339 | ATP2A3 |
| 4.52E-06 | -0.303217939 | 0.366 | 0.545 | 0.08139752 | RBM38 |
| 4.52E-06 | -0.319889986 | 0.621 | 0.788 | 0.081488208 | LRMP |
| 4.56E-06 | 0.441929205 | 0.666 | 0.564 | 0.082168941 | H3F3A |
| 4.62E-06 | -0.256003165 | 0.066 | 0.151 | 0.083266137 | BCOR |
| 4.69E-06 | -0.260028209 | 0.238 | 0.404 | 0.084487456 | NCKAP1L |
| 4.69E-06 | -0.300307325 | 0.085 | 0.179 | 0.084508474 | ID2 |
| 4.85E-06 | -0.29534603 | 0.183 | 0.317 | 0.087434775 | MIDN |
| 4.87E-06 | -0.292869519 | 0.275 | 0.439 | 0.087816801 | BTK |
| 5.04E-06 | -0.346284413 | 0.484 | 0.654 | 0.090821652 | REL |
| 5.11E-06 | -0.25545788 | 0.92 | 0.958 | 0.092051293 | CD37 |
| 5.64E-06 | -0.258127435 | 0.152 | 0.276 | 0.101623912 | ZNF101 |
| 5.81E-06 | -0.291478768 | 0.561 | 0.718 | 0.104694409 | NEIL1 |
| 5.93E-06 | -0.30835857 | 0.698 | 0.808 | 0.106904364 | GDI2 |
| 5.98E-06 | 0.453349885 | 0.737 | 0.67 | 0.107685366 | FTL |
| 5.98E-06 | -0.280199877 | 0.042 | 0.109 | 0.107840698 | LTN1 |
| 6.07E-06 | -0.430457884 | 0.353 | 0.516 | 0.109325551 | ARID4B |
| 6.14E-06 | -0.317431416 | 0.196 | 0.33 | 0.110591752 | IST1 |
| 6.18E-06 | -0.29347784 | 0.324 | 0.497 | 0.111287502 | SYVN1 |
| 6.36E-06 | -0.26280407 | 0.049 | 0.122 | 0.114599425 | GSAP |
| 6.60E-06 | 0.376296354 | 0.79 | 0.788 | 0.11888737 | RAN |
| 6.61E-06 | -0.390211123 | 0.235 | 0.375 | 0.119113138 | STK40 |
| 6.66E-06 | -0.269607682 | 0.99 | 1 | 0.119953593 | TPT1 |
| 6.79E-06 | -0.252153348 | 0.846 | 0.933 | 0.122419095 | YWHAB |
| 7.01E-06 | -0.287621184 | 0.49 | 0.708 | 0.126324721 | DAZAP2 |
| 7.32E-06 | -0.284901444 | 0.11 | 0.212 | 0.131902987 | TRIM44 |
| 7.43E-06 | -0.345827377 | 0.14 | 0.253 | 0.133924655 | PARN |
| 7.67E-06 | -0.257519731 | 0.18 | 0.311 | 0.138284473 | FAM214A |
| 7.85E-06 | -0.318377569 | 0.27 | 0.417 | 0.141536992 | MSI2 |
| 7.96E-06 | -0.272638432 | 0.34 | 0.516 | 0.143410526 | CD82 |
| 8.41E-06 | -0.280684574 | 0.2 | 0.333 | 0.151553673 | RAPGEF1 |
| 8.56E-06 | 0.368456987 | 0.821 | 0.792 | 0.154307964 | CHCHD2 |
| 8.87E-06 | -0.309307094 | 0.108 | 0.208 | 0.1598178 | NUP153 |
| 8.87E-06 | -0.265669503 | 0.251 | 0.407 | 0.159920797 | RHOG |
| 9.11E-06 | -0.366083678 | 0.246 | 0.397 | 0.164166936 | PTPN6 |
| 9.40E-06 | -0.354057625 | 0.391 | 0.567 | 0.169479825 | PTPN18 |
| 9.57E-06 | -0.355177419 | 0.079 | 0.163 | 0.172422724 | CDC25B |
| 9.83E-06 | -0.323792301 | 0.472 | 0.66 | 0.177111227 | SYK |
| 1.00E-05 | -0.30433035 | 0.202 | 0.34 | 0.180663901 | STAT3 |
| 1.01E-05 | -0.318772238 | 0.281 | 0.429 | 0.182800712 | TAGAP |
| 1.02E-05 | 0.41940569 | 0.754 | 0.734 | 0.184069944 | SNRPD2 |
| 1.03E-05 | -0.31386578 | 0.58 | 0.737 | 0.184752841 | HLA-DMB |
| 1.03E-05 | -0.380190581 | 0.085 | 0.173 | 0.186278438 | PCIF1 |
| 1.04E-05 | -0.301432811 | 0.381 | 0.561 | 0.187398609 | AES |
| 1.08E-05 | -0.273617518 | 0.174 | 0.301 | 0.193863711 | KLHL5 |
| 1.11E-05 | -0.339713326 | 0.124 | 0.228 | 0.199758333 | SEMA4D |
| 1.14E-05 | -0.273600889 | 0.443 | 0.638 | 0.205788054 | ATF4 |
| 1.16E-05 | -0.252254102 | 0.565 | 0.766 | 0.208833636 | GGA2 |
| 1.18E-05 | -0.283736361 | 0.323 | 0.49 | 0.213009488 | PGLS |
| 1.18E-05 | -0.269193924 | 0.048 | 0.115 | 0.213443067 | INPPL1 |
| 1.21E-05 | -0.276286804 | 0.421 | 0.593 | 0.218005438 | BLOC1S6 |
| 1.21E-05 | -0.311089791 | 0.195 | 0.321 | 0.218195245 | CUX1 |
| 1.25E-05 | -0.326256666 | 0.064 | 0.141 | 0.224445571 | AL512631.1 |
| 1.28E-05 | -0.269737378 | 0.127 | 0.234 | 0.229806381 | ATXN7L3B |
| 1.31E-05 | -0.330281049 | 0.77 | 0.865 | 0.23556774 | BASP1 |
| 1.37E-05 | -0.262354977 | 0.211 | 0.353 | 0.24648793 | ADAR |
| 1.45E-05 | 0.384401428 | 0.764 | 0.747 | 0.260918617 | COX6B1 |
| 1.47E-05 | -0.363440392 | 0.125 | 0.231 | 0.264068073 | MAPKAPK2 |
| 1.54E-05 | 0.328695885 | 0.909 | 0.881 | 0.276665024 | TMSB10 |
| 1.54E-05 | -0.301378644 | 0.074 | 0.154 | 0.277810707 | PPTC7 |
| 1.56E-05 | -0.253185534 | 0.358 | 0.538 | 0.281603006 | MCL1 |
| 1.63E-05 | -0.33581494 | 0.083 | 0.17 | 0.294010128 | DYNLL2 |
| 1.67E-05 | -0.612054117 | 0.576 | 0.718 | 0.301525571 | IGHM |
| 1.69E-05 | -0.267929458 | 0.269 | 0.426 | 0.305226204 | PIK3AP1 |
| 1.73E-05 | -0.301550862 | 0.075 | 0.157 | 0.311992168 | ARHGAP1 |
| 1.75E-05 | -0.250963049 | 0.052 | 0.122 | 0.31605663 | GSK3B |
| 1.81E-05 | -0.284816883 | 0.642 | 0.801 | 0.326367516 | HLA-E |
| 1.88E-05 | -0.30798787 | 0.075 | 0.157 | 0.3389289 | GLCCI1 |
| 1.92E-05 | -0.368608034 | 0.289 | 0.446 | 0.345658347 | SMARCA4 |
| 2.07E-05 | -0.267634707 | 0.106 | 0.202 | 0.373091263 | NOL11 |
| 2.13E-05 | -0.378655217 | 0.284 | 0.426 | 0.384079202 | CD180 |
| 2.20E-05 | -0.307119542 | 0.339 | 0.497 | 0.396151484 | SKAP2 |
| 2.20E-05 | -0.306348493 | 0.42 | 0.593 | 0.396792384 | CPNE5 |
| 2.29E-05 | -0.335425894 | 0.38 | 0.558 | 0.412414879 | DCAF12 |
| 2.40E-05 | -0.312108942 | 0.582 | 0.776 | 0.43192888 | SRSF5 |
| 2.42E-05 | -0.261079113 | 0.075 | 0.157 | 0.436967593 | SCAF8 |
| 2.45E-05 | -0.295201731 | 0.097 | 0.186 | 0.442317834 | TRIM25 |
| 2.55E-05 | -0.306237532 | 0.143 | 0.25 | 0.460069249 | ZFR |
| 2.65E-05 | -0.25833379 | 0.172 | 0.295 | 0.477427255 | SUSD6 |
| 2.68E-05 | -0.323997265 | 0.075 | 0.154 | 0.483082722 | TMEM205 |
| 2.80E-05 | -0.276597439 | 0.076 | 0.157 | 0.504983256 | DCTN1 |
| 2.81E-05 | -0.255574492 | 0.159 | 0.276 | 0.507113139 | HELZ |
| 2.90E-05 | -0.255217342 | 0.136 | 0.24 | 0.523023553 | EIF4G1 |
| 2.94E-05 | -0.293109762 | 0.249 | 0.391 | 0.530642475 | SLC15A4 |
| 2.96E-05 | -0.359365242 | 0.086 | 0.17 | 0.53287402 | SLC1A4 |
| 3.01E-05 | -0.350505517 | 0.15 | 0.26 | 0.54324153 | OAS1 |
| 3.14E-05 | -0.287830538 | 0.082 | 0.163 | 0.565694158 | DIS3 |
| 3.18E-05 | 0.550397374 | 0.559 | 0.522 | 0.572612237 | NDUFB1 |
| 3.22E-05 | 0.853650151 | 0.314 | 0.234 | 0.580357087 | FABP5 |
| 3.24E-05 | -0.293151384 | 0.572 | 0.753 | 0.583110806 | BCL7A |
| 3.39E-05 | -0.263011148 | 0.042 | 0.103 | 0.610620677 | ENDOD1 |
| 3.45E-05 | 0.443031618 | 0.125 | 0.045 | 0.621949733 | PITPNA-AS1 |
| 3.46E-05 | -0.364194582 | 0.439 | 0.603 | 0.622854684 | BTG2 |
| 3.48E-05 | -0.307197388 | 0.177 | 0.295 | 0.627396021 | LRP10 |
| 3.69E-05 | -0.254100146 | 0.223 | 0.362 | 0.664462047 | PHIP |
| 3.76E-05 | -0.266484629 | 0.309 | 0.462 | 0.677378071 | SP100 |
| 3.77E-05 | -0.348360546 | 0.08 | 0.16 | 0.679834476 | MARK2 |
| 3.90E-05 | -0.277437372 | 0.068 | 0.144 | 0.702277032 | KAT6B |
| 4.00E-05 | -0.277647162 | 0.9 | 0.971 | 0.720515698 | EZR |
| 4.04E-05 | -0.263872652 | 0.114 | 0.205 | 0.72822444 | ELMO1 |
| 4.06E-05 | -0.361137864 | 0.212 | 0.333 | 0.731713129 | RASSF6 |
| 4.20E-05 | -0.265210389 | 0.175 | 0.295 | 0.756693146 | SPCS3 |
| 4.25E-05 | -0.271781707 | 0.109 | 0.202 | 0.766661981 | NFATC2IP |
| 4.33E-05 | 0.742911869 | 0.72 | 0.747 | 0.779554902 | H2AFZ |
| 4.49E-05 | -0.301730364 | 0.703 | 0.84 | 0.808845021 | RAC2 |
| 4.77E-05 | 0.407230992 | 0.975 | 0.981 | 0.859929685 | MALAT1 |
| 5.15E-05 | -0.282100253 | 0.391 | 0.548 | 0.92764936 | PRRC2C |
| 5.25E-05 | -0.277048086 | 0.144 | 0.25 | 0.946900552 | HTR3A |
| 5.31E-05 | -0.299440121 | 0.139 | 0.24 | 0.95749561 | RNF41 |
| 5.34E-05 | -0.412012033 | 0.092 | 0.173 | 0.963048821 | NORAD |
| 5.49E-05 | -0.267367816 | 0.317 | 0.462 | 0.988747917 | AKAP13 |
| 5.53E-05 | -0.442159713 | 0.069 | 0.141 | 0.996962632 | RETREG3 |
| 5.64E-05 | -0.26952964 | 0.138 | 0.244 | 1 | TMEM154 |
| 5.89E-05 | -0.301029072 | 0.072 | 0.147 | 1 | AHCYL1 |
| 5.93E-05 | -0.33997228 | 0.411 | 0.574 | 1 | RAD21 |
| 5.98E-05 | -0.308462128 | 0.129 | 0.231 | 1 | ANKRD17 |
| 6.06E-05 | -0.397442229 | 0.119 | 0.212 | 1 | PTMS |
| 6.30E-05 | -0.265020908 | 0.068 | 0.141 | 1 | CCNT1 |
| 6.32E-05 | -0.311448105 | 0.155 | 0.263 | 1 | ZNF24 |
| 6.40E-05 | 0.4677561 | 0.586 | 0.567 | 1 | C6orf48 |
| 6.47E-05 | -0.321188855 | 0.093 | 0.176 | 1 | DCAF11 |
| 6.71E-05 | 0.444174192 | 0.65 | 0.651 | 1 | ATPIF1 |
| 6.92E-05 | 0.30660011 | 0.764 | 0.731 | 1 | HINT1 |
| 6.95E-05 | -0.32812415 | 0.497 | 0.651 | 1 | HMCES |
| 7.22E-05 | -0.285082849 | 0.058 | 0.125 | 1 | NXPE3 |
| 7.71E-05 | -0.265715258 | 0.732 | 0.856 | 1 | PTPRC |
| 7.87E-05 | -0.31220095 | 0.171 | 0.282 | 1 | PEX5 |
| 7.96E-05 | -0.279961783 | 0.166 | 0.272 | 1 | MICU2 |
| 8.13E-05 | 0.499515774 | 0.579 | 0.545 | 1 | MRPL51 |
| 8.16E-05 | 0.431255794 | 0.675 | 0.679 | 1 | SKP1 |
| 8.32E-05 | -0.381967307 | 0.261 | 0.391 | 1 | KHSRP |
| 8.38E-05 | -0.260651202 | 0.391 | 0.571 | 1 | ZNF207 |
| 8.61E-05 | -0.252501156 | 0.145 | 0.25 | 1 | KLC1 |
| 8.66E-05 | -0.309660099 | 0.245 | 0.378 | 1 | DOCK8 |
| 8.68E-05 | -0.26052076 | 0.123 | 0.221 | 1 | FEZ2 |
| 8.77E-05 | -0.415815597 | 0.298 | 0.436 | 1 | TERF2 |
| 9.30E-05 | -0.27761261 | 0.243 | 0.369 | 1 | RALGPS2 |
| 9.39E-05 | -0.323481757 | 0.181 | 0.292 | 1 | CCNG2 |
| 9.50E-05 | -0.28858812 | 0.285 | 0.426 | 1 | RNGTT |
| 9.53E-05 | -0.310624496 | 0.183 | 0.301 | 1 | CREB1 |
| 0.000103177 | -0.318120749 | 0.377 | 0.545 | 1 | GGNBP2 |
| 0.000104173 | -0.256237709 | 0.186 | 0.301 | 1 | PTBP3 |
| 0.000106276 | -0.292025729 | 0.079 | 0.154 | 1 | RRP1B |
| 0.000108981 | -0.306832488 | 0.196 | 0.308 | 1 | ST14 |
| 0.000114123 | -0.260426969 | 0.243 | 0.369 | 1 | DNAJC10 |
| 0.000116578 | -0.345980526 | 0.575 | 0.74 | 1 | DAAM1 |
| 0.000118846 | 0.310349648 | 0.825 | 0.792 | 1 | SRP14 |
| 0.000120227 | -0.264742397 | 0.276 | 0.423 | 1 | VOPP1 |
| 0.000120368 | -0.316369612 | 0.3 | 0.452 | 1 | STIM2 |
| 0.000124124 | -0.252210931 | 0.075 | 0.147 | 1 | GIGYF2 |
| 0.000128723 | -0.288366197 | 0.145 | 0.24 | 1 | FLOT2 |
| 0.000128915 | -0.251181393 | 0.196 | 0.311 | 1 | AFTPH |
| 0.000133274 | 0.527364002 | 0.352 | 0.256 | 1 | SNHG7 |
| 0.000134837 | -0.276722793 | 0.111 | 0.199 | 1 | RB1CC1 |
| 0.00013493 | -0.25382254 | 0.193 | 0.311 | 1 | DR1 |
| 0.000136852 | -0.268689024 | 0.221 | 0.34 | 1 | EDEM1 |
| 0.000137657 | -0.259324803 | 0.168 | 0.276 | 1 | OGT |
| 0.000138983 | 0.485605348 | 0.582 | 0.574 | 1 | COX5A |
| 0.000141094 | -0.349279385 | 0.13 | 0.218 | 1 | TRAK1 |
| 0.000141259 | -0.25142451 | 0.581 | 0.715 | 1 | HLA-DQA1 |
| 0.00014407 | -0.258610524 | 0.157 | 0.263 | 1 | PPP4R3B |
| 0.00014716 | -0.260883378 | 0.37 | 0.526 | 1 | PDIA3 |
| 0.000156859 | -0.327584711 | 0.107 | 0.192 | 1 | KDM5C |
| 0.000157228 | -0.271569327 | 0.17 | 0.279 | 1 | SAFB2 |
| 0.000157819 | -0.278977828 | 0.23 | 0.349 | 1 | GNA13 |
| 0.000200986 | -0.310210726 | 0.554 | 0.718 | 1 | BIRC3 |
| 0.00020813 | -0.299622808 | 0.416 | 0.564 | 1 | IKZF3 |
| 0.000214587 | -0.389448858 | 0.208 | 0.314 | 1 | FCRL3 |
| 0.00022327 | -0.273926604 | 0.632 | 0.766 | 1 | POU2F2 |
| 0.000223957 | 1.082790286 | 0.43 | 0.385 | 1 | TUBA1B |
| 0.000225751 | -0.375753356 | 0.13 | 0.215 | 1 | FAM53B |
| 0.000234273 | 0.414324051 | 0.257 | 0.404 | 1 | IGHG3 |
| 0.000237702 | -0.285492616 | 0.272 | 0.397 | 1 | ADAM19 |
| 0.00023803 | -0.2773671 | 0.142 | 0.234 | 1 | SMARCD2 |
| 0.000239532 | -0.259448162 | 0.1 | 0.179 | 1 | ZBTB20 |
| 0.000246056 | -0.264738331 | 0.625 | 0.766 | 1 | MEF2C |
| 0.000271881 | 0.606958837 | 0.625 | 0.625 | 1 | TUBB |
| 0.000273602 | -0.281765438 | 0.265 | 0.391 | 1 | ARHGAP30 |
| 0.000276327 | 0.431861085 | 0.634 | 0.638 | 1 | ATP5O |
| 0.000277778 | -0.3334389 | 0.122 | 0.208 | 1 | DHTKD1 |
| 0.000279004 | -0.265286662 | 0.25 | 0.375 | 1 | CLTC |
| 0.000280019 | -0.252183555 | 0.13 | 0.218 | 1 | EIF4EBP2 |
| 0.000314964 | -0.378571901 | 0.11 | 0.189 | 1 | CLPTM1 |
| 0.000319709 | -0.35829077 | 0.123 | 0.205 | 1 | LMO4 |
| 0.000326292 | -0.284159727 | 0.18 | 0.282 | 1 | PHF6 |
| 0.000329441 | -0.293515262 | 0.149 | 0.24 | 1 | RGS16 |
| 0.000347788 | -0.408917644 | 0.125 | 0.205 | 1 | CERK |
| 0.000357597 | -0.330380569 | 0.081 | 0.151 | 1 | ZNF652 |
| 0.000373936 | 0.318364914 | 0.731 | 0.753 | 1 | ATP5G3 |
| 0.000390363 | -0.27699125 | 0.348 | 0.481 | 1 | CD38 |
| 0.000414974 | -0.269901643 | 0.108 | 0.186 | 1 | LPCAT1 |
| 0.000429682 | -0.326678776 | 0.101 | 0.176 | 1 | PIK3CD |
| 0.000456319 | -0.260668151 | 0.071 | 0.135 | 1 | ZMIZ2 |
| 0.000458014 | 0.462449285 | 0.544 | 0.522 | 1 | NDUFA11 |
| 0.000480473 | -0.256402666 | 0.152 | 0.244 | 1 | KCTD20 |
| 0.000485565 | 0.384850786 | 0.643 | 0.587 | 1 | RGS13 |
| 0.000489543 | -0.458849514 | 0.147 | 0.231 | 1 | ALDH5A1 |
| 0.000493747 | 0.484558152 | 0.496 | 0.455 | 1 | C19orf53 |
| 0.000498093 | -0.251251071 | 0.062 | 0.122 | 1 | TRIOBP |
| 0.000504614 | 0.367289785 | 0.667 | 0.644 | 1 | TBCA |
| 0.000511115 | -0.266674809 | 0.116 | 0.196 | 1 | CEP135 |
| 0.000529667 | -0.258648995 | 0.06 | 0.119 | 1 | BRCC3 |
| 0.000535554 | 0.441819677 | 0.582 | 0.545 | 1 | SRSF9 |
| 0.000545088 | 0.385210483 | 0.621 | 0.516 | 1 | EEF1D |
| 0.000556292 | -0.284965417 | 0.42 | 0.577 | 1 | MGEA5 |
| 0.000598861 | 0.461913507 | 0.537 | 0.471 | 1 | UBL5 |
| 0.00076316 | 0.309421462 | 0.688 | 0.667 | 1 | COX8A |
| 0.000768673 | 0.251900346 | 0.641 | 0.599 | 1 | TPI1 |
| 0.000781779 | -0.261845441 | 0.334 | 0.465 | 1 | ATP2B1 |
| 0.000793461 | 0.304979457 | 0.711 | 0.734 | 1 | ATP5J2 |
| 0.000807931 | 0.323145059 | 0.779 | 0.766 | 1 | PFDN5 |
| 0.00082614 | -0.294398588 | 0.071 | 0.131 | 1 | SLC35B4 |
| 0.000831229 | 0.323780904 | 0.82 | 0.808 | 1 | YBX1 |
| 0.000831379 | 0.305405905 | 0.638 | 0.625 | 1 | SOD1 |
| 0.000837847 | -0.250652228 | 0.207 | 0.308 | 1 | PSMB8-AS1 |
| 0.000838475 | 0.320750521 | 0.643 | 0.535 | 1 | FTH1 |
| 0.000845024 | 0.50444696 | 0.481 | 0.458 | 1 | NAA38 |
| 0.000900092 | -0.262397571 | 0.138 | 0.221 | 1 | PEA15 |
| 0.000924079 | -0.251184376 | 0.067 | 0.125 | 1 | EEA1 |
| 0.001038809 | -0.250675184 | 0.102 | 0.173 | 1 | LOXL2 |
| 0.00104671 | -0.297393355 | 0.07 | 0.128 | 1 | ANGEL2 |
| 0.001061856 | 0.481212866 | 0.263 | 0.186 | 1 | RGCC |
| 0.001137381 | -0.279973754 | 0.095 | 0.16 | 1 | PLXNB2 |
| 0.001166158 | 0.492706873 | 0.104 | 0.048 | 1 | UCHL1 |
| 0.001168451 | -0.317654918 | 0.054 | 0.106 | 1 | ZNF37A |
| 0.001283821 | -0.254935899 | 0.094 | 0.16 | 1 | GLUL |
| 0.001353224 | -0.252208311 | 0.077 | 0.138 | 1 | MSL1 |
| 0.001405975 | 0.364528431 | 0.612 | 0.587 | 1 | SEC61G |
| 0.001418362 | 0.422481385 | 0.543 | 0.538 | 1 | SNRPF |
| 0.00146857 | -0.28801126 | 0.067 | 0.122 | 1 | SLA |
| 0.001510537 | 0.620522659 | 0.398 | 0.365 | 1 | PRELID1 |
| 0.00154929 | -0.29405196 | 0.238 | 0.333 | 1 | DNASE1 |
| 0.001570942 | 0.325327739 | 0.759 | 0.763 | 1 | CALM2 |
| 0.001588265 | 0.328558084 | 0.683 | 0.635 | 1 | DBI |
| 0.001710678 | -0.501878041 | 0.102 | 0.163 | 1 | BMP7 |
| 0.001776521 | -0.290220518 | 0.374 | 0.506 | 1 | GCSAM |
| 0.002030866 | -0.265419175 | 0.123 | 0.196 | 1 | CSNK1E |
| 0.002095849 | -0.263183606 | 0.294 | 0.407 | 1 | NR3C1 |
| 0.00209932 | -0.267768264 | 0.389 | 0.519 | 1 | ODC1 |
| 0.002301269 | -0.463908819 | 0.229 | 0.317 | 1 | TRBC2 |
| 0.002476085 | 0.476938662 | 0.442 | 0.391 | 1 | CD27 |
| 0.002507912 | 0.355830542 | 0.659 | 0.676 | 1 | OST4 |
| 0.002508011 | 0.543239483 | 0.366 | 0.327 | 1 | ATP5G1 |
| 0.002617369 | -0.368852251 | 0.071 | 0.125 | 1 | DCAF16 |
| 0.002647831 | 0.313384778 | 0.712 | 0.679 | 1 | DYNLL1 |
| 0.00273038 | -0.274355332 | 0.097 | 0.157 | 1 | ATP5EP2 |
| 0.002769745 | -0.270990444 | 0.19 | 0.276 | 1 | VIM |
| 0.0028104 | 0.404983318 | 0.578 | 0.567 | 1 | SNRPD1 |
| 0.002862617 | 0.345786062 | 0.648 | 0.657 | 1 | RBX1 |
| 0.003232219 | 0.379456118 | 0.67 | 0.708 | 1 | COX7A2 |
| 0.003350657 | -0.258469329 | 0.359 | 0.462 | 1 | IGHD |
| 0.003350733 | 0.917165788 | 0.397 | 0.333 | 1 | IGLC3 |
| 0.003511902 | 0.29681529 | 0.737 | 0.699 | 1 | EIF3E |
| 0.003521617 | 0.364337532 | 0.637 | 0.599 | 1 | STMN1 |
| 0.003594998 | 0.690975795 | 0.4 | 0.388 | 1 | RANBP1 |
| 0.003726457 | 0.36319752 | 0.583 | 0.622 | 1 | ROMO1 |
| 0.003750061 | -0.298170804 | 0.156 | 0.224 | 1 | TUG1 |
| 0.003764202 | 0.626475942 | 0.287 | 0.237 | 1 | CKS2 |
| 0.0040471 | 0.606806783 | 0.144 | 0.09 | 1 | TK1 |
| 0.004363831 | -0.274604709 | 0.396 | 0.526 | 1 | PAX5 |
| 0.004410644 | 0.391461757 | 0.526 | 0.522 | 1 | POLR1D |
| 0.004582118 | 0.416125369 | 0.498 | 0.478 | 1 | HIGD2A |
| 0.004668286 | 0.558622469 | 0.435 | 0.404 | 1 | RPA3 |
| 0.005429034 | 0.362429936 | 0.651 | 0.644 | 1 | PRDX1 |
| 0.005444891 | -0.269656447 | 0.63 | 0.737 | 1 | CALM1 |
| 0.00606326 | 0.346556775 | 0.614 | 0.619 | 1 | LSM7 |
| 0.006695783 | 0.270606297 | 0.723 | 0.737 | 1 | ENO1 |
| 0.006716015 | 0.391854761 | 0.51 | 0.49 | 1 | PSMA2 |
| 0.006873827 | 0.331882401 | 0.653 | 0.66 | 1 | H2AFV |
| 0.007067745 | 0.389178508 | 0.639 | 0.635 | 1 | PSMA7 |
| 0.00722229 | 0.311561773 | 0.669 | 0.708 | 1 | ERH |
| 0.007369183 | 0.462761547 | 0.49 | 0.471 | 1 | UQCR10 |
| 0.00760427 | 0.418611622 | 0.452 | 0.433 | 1 | PRDX3 |
| 0.008317356 | 0.443416235 | 0.334 | 0.288 | 1 | ACY3 |
| 0.008992211 | 0.329956671 | 0.522 | 0.497 | 1 | NCF1 |
| 0.009269436 | 0.322197878 | 0.698 | 0.699 | 1 | SUMO2 |
| 0.009627009 | 0.402234437 | 0.58 | 0.574 | 1 | MZT2B |
| 0.009685853 | -0.304839362 | 0.093 | 0.144 | 1 | PPP1R9B |
| 0.009735278 | 0.352273771 | 0.616 | 0.606 | 1 | ATP5J |

Table S6. Proportion of CD4^+^ T cell subpopulations in adenoid tissue sorted by Mass Cytometry.

| SubCellType | ANH | AH | TH | logFC_ANH_vs_AH | logFC_ANH_vs_TH | logFC_AH_vs_TH |
| --- | --- | --- | --- | --- | --- | --- |
| Tfh_PD-1 | 0.300339477 | 0.386332608 | 0.421762915 | -0.363249335 | -0.48983813 | -0.126588794 |
| CD4.T_CD127 | 0.374985367 | 0.254457748 | 0.22527567 | 0.559408182 | 0.735142788 | 0.175734606 |
| CD4.T_CCR7 | 0.141734844 | 0.195338292 | 0.170993102 | -0.462780318 | -0.270743653 | 0.192036665 |
| Treg_IL2RA | 0.1270632 | 0.113357108 | 0.126756808 | 0.1646714 | 0.003483023 | -0.161188377 |
| Th17_CCR6 | 0.049009534 | 0.040750639 | 0.045870534 | 0.26623974 | 0.095494732 | -0.170745008 |
| Th2_CXCR3 | 0.006867578 | 0.009763605 | 0.009340971 | -0.507612526 | -0.443771173 | 0.063841352 |

Abbreviations: AH, adenoid hypertrophy; ANH, adenoid non-hypertrophy.

Table S7. Primer sequences for RT-qPCR.

|  | Sequence (5' -> 3') |
| --- | --- |
| HSP90B1 | |
| Forward Primer | CCAGTTTGGTGTCGGTTTCTAT |
| Reverse Primer | CTGGGTATCGTTGTTGTGTTTTG |
| XBP1 | |
| Forward Primer | CCCTCCAGAACATCTCCCCAT |
| Reverse Primer | ACATGACTGGGTCCAAGTTGT |
| DERL3 | |
| Forward Primer | GGCGTCCTTATGACCCTGC |
| Reverse Primer | AGGTCCACGAGGATGGAGTT |
| HSPA5 | |
| Forward Primer | CACGGTCTTTGACGCCAAG |
| Reverse Primer | CCAAATAAGCCTCAGCGGTTT |
| PDIA4 | |
| Forward Primer | CACGCTTGTGTTGACCAAAGA |
| Reverse Primer | AATTGGAGGAGAACGCTTGCT |
| HERPUD1 | |
| Forward Primer | ATGGAGTCCGAGACCGAAC |
| Reverse Primer | TTGGTGATCCAACAACAGCTT |
| BIK | |
| Forward Primer | GACCTGGACCCTATGGAGGAC |
| Reverse Primer | CCTCAGTCTGGTCGTAGATGA |
| LMAN1 | |
| Forward Primer | AGTGTAGGAGATCGAGAGCTAAG |
| Reverse Primer | AGTTCTTGTTGAGTAATCTGCCC |
| FOXP1 | |
| Forward Primer | TGGCATCTCATAAACCATCAGC |
| Reverse Primer | GGTCCACTCATCTTCGTCTCAG |
| GAPDH | |
| Forward Primer | CTGGGCTACACTGAGCACC |
| Reverse Primer | AAGTGGTCGTTGAGGGCAATG |

Table S8. shRNA sequence information.

| Gene | Strand | Sequences (5' -> 3') |
| --- | --- | --- |
| FOXP1 | sense | TGCGAAGATTTCCAATCATTT |
|  | antisense | AAATGATTGGAAATCTTCGCA |
| TIGIT | sense | GTCGCTGACCGTGAACGATAC |
|  | antisense | GTATCGTTCACGGTCAGCGAC |
| LTA | sense | GCATCTACTTCGTCTACTCCC |
|  | antisense | GGGAGTAGACGAAGTAGATGC |
| CD6 | sense | GCTCCTGGCGGTTCAACAACT |
|  | antisense | AGTTGTTGAACCGCCAGGAGC |
| PRR3 | sense | GAAGACAAATCCGACCGCCCT |
|  | antisense | AGGGCGGTCGGATTTGTCTTC |
| CD2 | sense | GGACTTGTATCAACACAACCC |
|  | antisense | GGGTTGTGTTGATACAAGTCC |
| ALCAM | sense | GCCGACTTGACGTACCTCAGA |
|  | antisense | TCTGAGGTACGTCAAGTCGGC |
| ICAM3 | sense | GAATGCGACAGTCATGAACCA |
|  | antisense | TGGTTCATGACTGTCGCATTC |
| CD58 | sense | GCCATCGAGGACTTATAATGT |
|  | antisense | ACATTATAAGTCCTCGATGGC |
| TNF | sense | GTCAGATCATCTTCTCGAACC |
|  | antisense | GGTTCGAGAAGATGATCTGAC |
| ALB2 | sense | GATGTATGGCTGACAAGACGC |
|  | antisense | GCGTCTTGTCAGCCATACATC |
| TNFRSF1B | sense | ACCAGATCTGTAACGTGGTGG |
|  | antisense | CCACCACGTTACAGATCTGG |
